# Evolutionary History and Distribution Analysis of Rhamnosyltransferases in the Fungal Kingdom

**DOI:** 10.3390/jof11070524

**Published:** 2025-07-15

**Authors:** Joaquín O. Chávez-Santiago, Luz A. López-Ramírez, Luis A. Pérez-García, Iván Martínez-Duncker, Bernardo Franco, Israel E. Padilla-Guerrero, Vianey Olmedo-Monfil, J. Félix Gutiérrez-Corona, Gustavo A. Niño-Vega, Jorge H. Ramírez-Prado, Héctor M. Mora-Montes

**Affiliations:** 1 Departamento de Biología, División de Ciencias Naturales y Exactas, Universidad de Guanajuato, Guanajuato 36050, Guanajuato, Mexico; jo.chavezsantiago@ugto.mx (J.O.C.-S.); adrianalr@ugto.mx (L.A.L.-R.); bfranco@ugto.mx (B.F.); ie.padillaguerrero@ugto.mx (I.E.P.-G.); vg.olmedo@ugto.mx (V.O.-M.); felixg@ugto.mx (J.F.G.-C.); gustavo.nino@ugto.mx (G.A.N.-V.); 2 Facultad de Estudios Profesionales Zona Huasteca, Universidad Autónoma de San Luis Potosí, Romualdo del Campo 501, Fracc. Rafael Curiel, Ciudad Valles 79060, San Luis Potosí, Mexico; luisantonio.perez@uaslp.mx; 3 Laboratorio de Glicobiología Humana y Diagnóstico Molecular, Centro de Investigación en Dinámica Celular, Instituto de Investigación en Ciencias Básicas y Aplicadas, Universidad Autónoma del Estado de Morelos, Cuernavaca 62209, Morelos, Mexico; duncker@uaem.mx; 4 Unidad de Biotecnología, Centro de Investigación Científica de Yucatán, A. C., Calle 43 No. 130, Col. Chuburná de Hidalgo, Mérida 97205, Yucatán, Mexico

**Keywords:** rhamnosyltransferases, evolutionary history, fungal kingdom, protein glycosylation, hidden Markov models

## Abstract

Rhamnose is a natural sugar found in glycoproteins and structural polysaccharides of plants, fungi, and bacteria. Its incorporation into glycoconjugates is mediated by rhamnosyltransferases (RHTs), key enzymes for biomolecular stability and function. While rhamnose biosynthesis has been studied in certain fungal genera, the evolutionary history and distribution of RHTs across the fungal kingdom remain largely unknown. In this study, 351 fungal species were found to encode putative RHTs. Phylogenetic and structural analyses revealed conserved patterns and similarities with previously characterized RHTs. Molecular docking predicted a high affinity of these proteins for UDP-L-rhamnose, and in silico mutagenesis identified key residues potentially involved in substrate binding. Carbohydrate profiling confirmed the presence of rhamnose in the cell walls of multiple fungi, including *Aspergillus*, *Madurella*, *Metarhizium*, and *Trichoderma* species. Enzymatic assays further supported rhamnose transfer activity. These findings provide the first comprehensive in silico characterization of fungal RHTs, uncovering conserved sequence motifs despite overall diversity, which may be linked to functional adaptation in different fungal lineages.

## 1. Introduction

The kingdom Fungi originated approximately 500 to 1000 million years ago and is one of the most extensive eukaryotic lineages, comprising an estimated 1.5 to 5 million species [1,2]. Fungi inhabit almost all ecosystems and exhibit a wide variety of life cycles, morphologies, and metabolic strategies. They interact with other organisms by developing mutualistic, parasitic, and commensal relationships in both terrestrial and aquatic environments [2]. Their cellular structures range from unicellular forms to complex filamentous networks that can give rise to macroscopic structures [3,4]. Fungi have abilities that allow them to thrive in different environments, colonize plant and animal cells, and contribute to the nutrient cycle in both terrestrial and aquatic environments [4].

The fungal cell wall is responsible for safeguarding cellular integrity, working as a barrier to withstand the various conditions to which these organisms are subjected. Its characteristics can vary depending on the species, but it is generally enriched with polysaccharides that can vary in composition and structural organization [5,6]. Among the most important cell wall structural components are glycoproteins, chitin, β-1,3-glucans, and β-1,6-glucans [5,7,8]. Glycoproteins are assembled through the action of glycosyltransferases (GTs), enzymes found in animals, protists, plants, bacteria, and fungi. These enzymes play key roles in biological processes by transferring sugar moieties from activated nucleotide sugar donors to specific acceptor molecules, such as carbohydrates, lipids, and proteins. GTs are involved in cell wall biosynthesis and remodeling, as well as in the glycosylation of a wide range of metabolites [9,10]. UDP-glycosyltransferases (UGTs) belong to the GT1 family, possess GT-B folds, and are among the most studied GTs [11]. These enzymes catalyze the transfer of glycosyl moieties from nucleotide-activated sugars, such as UDP-glucose, UDP-galactose, UDP-xylose, and UDP-rhamnose, to glycosyl acceptor molecules, which include polysaccharides, proteins, lipids, and secondary metabolites [12,13,14]. Among these sugars, UDP-rhamnose has been studied to a lesser extent, despite being relevant for the cellular viability of some fungi. This is the case for genera such as *Sporothrix* and *Scedosporium*, where rhamnose-based glycoconjugates are structural cell wall components [13,14,15]. In *Paracoccidioides brasiliensis*, rhamnose is part of the glucuronoxylomannan-like glycans, a heteropolysaccharide essential for its virulence [16]. UGTs responsible for transferring rhamnose moieties from UDP-rhamnose are known as rhamnosyltransferases (RHTs), and biochemical analyses revealed the existence of two UDP-rhamnose-dependent rhamnosyltransferases in *S. schenckii* [8].

Although UGT protein sequences from different species do not exhibit high identity, UGT structures possess GT-B folds that show high conservation [9]. The GT-B fold comprises two independent Rossman-type β/α/β domains, consisting of an N-terminal domain and a C-terminal domain, which are positioned face-to-face and connected by an interdomain cleft. These domains are responsible for recognizing and binding UDP-sugar donors with their respective acceptors [10,17].

In the kingdom Plantae, the presence of RHTs has been confirmed, for example, in *Arabidopsis thaliana*, whose cell wall contains pectin, specifically rhamnogalacturonan I (RG-I). The enzyme RRT1, belonging to the GT106 glycosyltransferase family, participates in RG-I synthesis by transferring rhamnose from UDP-β-L-rhamnose, playing a key role in the formation of the plant cell wall [18]. In the case of the kingdom Fungi, the available information is insufficient to detail the rhamnosyltransferases’ evolutionary history. In this study, we aimed to explore the evolutionary history and distribution of putative fungal RHTs through a combined in silico and in vitro approach. To identify these hypothetical genes, we used hidden Markov model (HMM) profiles, which identified the main motifs found in these sequences, and compared them to analyze their distribution across different taxonomic groups within the kingdom Fungi. Additionally, we performed molecular docking assays to predict the binding affinity between the potential RHTs and UDP-rhamnose. To functionally validate these findings, we performed carbohydrate composition analyses and enzymatic assays in selected fungal species, allowing us to test whether the predicted RHT-like sequences correlate with measurable enzymatic activity. This integrated approach provided new insights into the potential roles and evolutionary patterns of RHTs in the kingdom Fungi.

## 2. Materials and Methods

### 2.1. Hardware and Software Environment Used

All bioinformatic analyses were conducted on an HP ENVY x360 with AMD Ryzen 3 2300u 2.00 GHz, 8 GB RAM, 256 GB SSD, an external ADATA HV620S 1 TB hard drive, and Windows Subsystem for Linux (WSL), a compatibility layer developed by Microsoft to run Linux binaries (in ELF format) natively on Windows 10. The analyses were performed on publicly accessible servers: NCBI databases [19], PFAM database [20], and AlphaFold2 hosted on Google Colab [21].

### 2.2. Downloading the NCBI Database

The non-redundant (NR) BLAST database was downloaded on 9 October 2023, directly from the NCBI platform. The command used for downloading was: sudo wget -p/mnt/adata https://ftp.ncbi.nlm.nih.gov/blast/db/.

### 2.3. Construction of Hidden Markov Models of Rhamnosyltransferases and HMMER Searches

Protein sequences of rhamnosyltransferases from *Sporothrix schenckii* (Genebank accession code given in brackets) SPSK_05538 (XP_016583713.1) and SPSK_01110 (XP_016584143.1) were downloaded directly from the NCBI database [8]. Additionally, a tblastn search was conducted within the genomes of *S. brasiliensis* (GCF_000820605.1), *S. globosa* (GCA_001630435.1), *S. dimorphospora* (GCA_021397985.1), *S. pallida* (GCA_021396235.1), *S. luriei* (GCA_021398005.1), *S. humícola* (GCA_021396245.1), *S. mexicana* (GCA_021396375.1), *S. phasma* (GCA_016097075.2), *S. variecibatus* (GCA_016097105.2), *S. inflata* (GCA_021396225.1), *S. euskadiensis* (GCA_019925375.1), *S. pseudoabietina* (GCA_019925295.1), *S. curviconia* (GCA_016097085.2), *S. brunneoviolacea* (GCA_021396205.1), *S.* cf. *nigrograna* (GCA_019925305.1), *S. protearum* (GCA_016097115.2), and *Niveomyces insectorum* (GCA_001636815.1).

Following this, a “blastdbcmd” command was used within WSL to retrieve nucleotide sequences from the analyzed species based on accession numbers and ranges obtained from tblastn searches [22] as follows:

blastdbcmd -db database_name -entry sequence_accession_number -range tblastn_range_obtained -out new_filename -outfmt output_format

Once amino acid sequences were obtained, multiple sequence alignment (MSA) was performed using the MAFFT algorithm (v. 7) [23].

For the Rht1 protein (SPSK_05538, XP_016583713.1), eight sequences were discarded due to high divergence. The remaining eight sequences (*S. schenckii*, *S. brasiliensis*, *S. globosa*, *S. mexicana*, *S. humícola*, *S. dimorphospora*, *S. inflata*, and *S. pallida*) were realigned using the MAFFT algorithm, and the resulting alignment was saved in STOCKHOLM format (SPSK_05538_MAFFT.sto). Similarly, for the Rht2 protein (SPSK_01110, XP_016584143.1), eight highly divergent sequences were discarded, and the remaining eight sequences (*S. schenckii*, *S. brasiliensis*, *S. globosa*, *S. mexicana*, *S. humícola*, *S. dimorphospora*, *S. inflata*, and *S. variecibatus*) were realigned and saved in STOCKHOLM format (SPSK_01110_MAFFT.sto).

In addition to the RHT sequences, orthologous sequences of the *RmlD* gene (which is involved in rhamnose biosynthesis), SPSK_06451 (XP_016591762.1), from genus *Sporothrix*, were also retrieved. A total of 13 sequences were obtained from the following species: *S. schenckii* (XP_016591762.1), *S. brasiliensis* (XP_040615952.1), *S. globosa* (LVYW01000006.1), *S. luriei* (WNLO01000074.1), *S. dimorphospora* (WOUA01000046.1), *S. inflata* (WNYF01000047.1), *S. pallida* (WNYG01000035.1), *S. humicola* (WNYE01000047.1), *S. mexicana* (WNYC01000019.1), *S. euskadiensis* (JADHKQ010000009.1), *S. pseudoabietina* (JADHKS010000001.1), *S. protearum* (JADMNH010000019.1), and *N. insectorum* (OAA58952.1). The 13 sequences were aligned using the MAFFT algorithm and saved in STOCKHOLM format (SPSK_06451_MAFFT.sto).

Subsequently, HMM profiles were generated from the MSAs of Rht1, Rht2, and RmlD using the “hmmbuild” command from locally installed HMMER (v. 3.3.2) on WSL [24].

hmmbuild SPSK_05538.hmm SPSK_05538_MAFFT.sto

hmmbuild SPSK_01110.hmm SPSK_01110_MAFFT.sto

hmmbuild SPSK_06451.hmm SPSK_06451_MAFFT.sto

Later, searches were conducted using the HMM profiles of Rht1, Rht2, and RmlD, using the “hmmsearch” command from HMMER, against the protein database of the kingdom Fungi obtained from NCBI (fungi_prot_db.fa).

Hmmsearch SPSK_05538.hmm fungi_prot_db.fa > SPSK_05538_MAFFT.hmmsearch

Hmmsearch SPSK_01110.hmm fungi_prot_db.fa > SPSK_01110_MAFFT.hmmsearch

Hmmsearch SPSK_06451.hmm fungi_prot_db.fa > SPSK_06451_MAFFT.hmmsearch

Finally, to determine the possible domains present in the retrieved sequences, an E-value cutoff of E < 1 × 10^−20^ was established [25]. These sequences were saved in a multi-sequence FASTA file format.

### 2.4. Distribution of Potential RHTs in the Kingdom Fungi

The distribution analysis of motifs in the putative RHTs was performed using the MEME Suite [26]. Protein sequences obtained from the searches conducted with HMMER for Rht1 and Rht2 were employed. The “Classic mode” configuration was used with “Any number of repetitions” in the site distribution, and the goal was to identify exactly five motifs. Advanced options maintained default conditions, adjusting only the motif size to search, with a minimum of “6” and a maximum of “20”.

To infer phylogenetic relationships of putative RHT proteins, sequence alignments were generated using MAFFT [23], and phylogenetic trees were constructed with PhyML 3.0 [27] using the parameters “-model BIC -Starting tress BioNJ -Fast likehood-based methods aLRT SH-like”.

### 2.5. Analysis of Three-Dimensional Structures of Putative RHTs

The sequences previously selected in the conserved motif analysis were used. Initially, a search was conducted in the Uniprot database to obtain the files containing a predicted three-dimensional model of the proteins in “Protein Data Bank” (.PDB) format [28]. Sequences whose three-dimensional structure was not found in the Uniprot database were modeled from their amino acid sequence using Alphafold 2 [21]. These files were subsequently analyzed using the PyMOL software (version 3.0) [29], which allows for the visualization of the three-dimensional structures and alignment between multiple structures.

RHTs putative sequences were analyzed using CB-Dock (version 2.0) and PyRx (version 1.1) [30,31], where interactions between ligands and proteins were analyzed. Additionally, Discovery Studio was also used for the 2D visualization of the protein–ligand docking complex structure [32]. This approach enabled the prediction of binding affinities between the putative RHTs and UDP-L-rhamnose (CID: 192751), UDP-glucose (CID: 8629), GDP-mannose (CID: 135398627), and dolichol phosphate mannose (SID: 5646075, all obtained from PubChem [33] database in SDF file format.

Docking analyses were performed using PyRx software, where ligands were processed in Open Babel for energy minimization through charge addition and optimization with the universal force field [34]. The binding energy values of the docked ligand–protein complexes were recorded in kcal/mol.

### 2.6. Site-Directed in Silico Mutagenesis

The ligand docker of CHARMM-GUI (https://www.charmm-gui.org; accessed on 28 November 2024) [35] was used to generate in silico mutants of selected putative RHTs. Default parameters were maintained in the *PDB Manipulation Options* section, except for *Mutation*, where the target amino acid was selected for modification. In the *Grid Generation* section, *Blind Docking* was selected to ensure that the search space fully encompassed the substrate-binding site. For solvation, an orthorhombic TIP3P water box was used with a padding of 10 Å around the protein, and the system was neutralized with KCl ions at a physiological concentration of 0.15 M. The docking environment was set at pH 7.0, and CHARMM36m force fields were applied for energy parameterization. UDP-L-rhamnose assessed which amino acids contributed to binding affinity after the mutation. Docking analyses were performed using the *AutoDock Vina* package (version 1.1.2).

### 2.7. Strains and Culture Conditions

Conidia were obtained from *Aspergillus niger* FGSC A732, *Madurella mycetomatis* (Laveran) Brumpt (ATCC 64942), *Metarhizium anisopliae* Xi-18-2, *M. brunneum* EC25, *M. guizhouense* HA11-2 (environmental isolate) [36], *Trichoderma atroviride* IMI 206,040 (ATCC 32173), *T. harzianum* T35, *T. reesei* RUTC30 (ATCC 56765), and *T. virens* Tv 29.8. Yeast-like cells were obtained from *S. schenckii* 1099-18 (ATCC MYA 4821), *Candida albicans* SC5314 (ATCC MYA-2876), and *Saccharomyces cerevisiae* BY4741 (ATCC 4040002).

All strains were cultured in YPD medium (1% yeast extract, 2% gelatin peptone, and 3% glucose). Cultures were incubated at 28 °C with shaking at 120 rpm, except for *S. schenckii* and *M. mycetomatis*, which were incubated at 37 °C under the same shaking conditions. *C. albicans* and *S. cerevisiae* were grown for 1 d; *Metarhizium, Madurella*, *Sporothrix,* and *Trichoderma* species for 4 d; and *A. niger* for 10 d.

### 2.8. Analysis of Cell Wall Composition

Conidial and yeast-like cells were pelleted, washed three times with deionized water, and disrupted using a Braun homogenizer (Braun Biotech International GmbH, Melsungen, Germany), as previously described [37,38]. The resulting cell walls were washed by centrifuging and resuspended six times in deionized water. Further purification was performed by serial incubations with SDS, β-mercaptoethanol, 1 mM EDTA (pH 7.5), and 50 mM Tris-HCl buffer to remove intracellular contaminants. As previously reported, samples were hydrolyzed with 2 M trifluoroacetic acid [38,39].

The acid-hydrolyzed cell wall samples were analyzed by high-performance anion-exchange chromatography with pulsed amperometric detection (HPAEC-PAD) using a Dionex system (Thermo Fisher Scientific, Waltham, MA, USA) under separation conditions like those previously described [37].

### 2.9. Enzyme Activity

Rhamnosyltransferase activity was analyzed using the supernatant obtained from cell disruption with a Braun homogenizer. For the enzyme assays, 200 ng of α-1,6-mannobiose (Dextra Laboratories, Reading, UK) was used as the rhamnose acceptor, and 500 µM of UDP-L-rhamnose (Chemily Glycoscience, Peachtree Corners, GA, USA) as the donor substrate. Three experimental conditions were established as follows: (i) a complete reaction containing the enzyme, the acceptor, and UDP-L-rhamnose; (ii) a no-acceptor control, in which the reaction was performed only with the enzyme and UDP-L-rhamnose; and (iii) a heat-inactivated enzyme control, in which the complete reaction mixture was subjected to thermal treatment at 50 °C for 60 min to inactivate the enzyme. Reactions were carried out in a final volume of 100 µL using a potassium phosphate buffer solution (50 mM, pH 7.0), preincubated at 37 °C for 2 min [8]. Reaction products were analyzed by HPAEC-PAD using a Dionex system (Thermo Fisher Scientific) equipped with a CarboPac PA-1 column. Separation conditions were like those previously described for the analysis of cell wall composition [37].

### 2.10. Statistical Analysis

Statistical analyses were performed using GraphPad Prism 6 software. In vitro experimental data were assessed for normality using the Shapiro–Wilk test. Since data showed a normal distribution, results were analyzed with the Student’s *t* test. Statistical significance was set at *p* < 0.05. All data were represented with mean and standard deviation.

## 3. Results

### 3.1. HMM Construction of RHTs and Searches with HMMER

After constructing the HMM profiles for Rht1 and Rht2, searches were performed using the fungal protein database from NCBI. A total of 302 genera were identified for Rht1 and 180 genera for Rht2.

To support the presence of a complete rhamnose biosynthetic pathway, a search for orthologs of the enzyme RmlD was conducted. RmlD is a UDP-4-keto-6-deoxyglucose-3,5-epimerase/-4-reductase responsible for the final step in UDP-L-rhamnose synthesis in *S. schenckii* [35]. Based on this, an HMM profile was constructed, identifying RmlD orthologs in 638 fungal genera. The comparison of RmlD and Rht1 revealed 263 shared genera, accounting for 87.09% of the 302 initial genera for Rht1. Likewise, 172 shared genera were found for Rht2 and RmlD, representing 95.56% of the 180 initially identified genera, suggesting a conserved rhamnose biosynthetic context.

For Rht1, 720 species were identified across 263 genera, while 438 species belonging to 172 genera were obtained for Rht2. Table 1 shows a representative subset of species containing putative RHTs identified through our HMMER-based searches. The full dataset, including all accession numbers, is available in Supplementary Table S1. Among the most frequently represented genera for both Rht1 and Rht2 were *Fusarium*, *Colletotrichum*, *Aspergillus*, *Trichoderma*, and *Claviceps*.

### 3.2. Phylogenetic Distribution of Putative RHTs

The phylogenetic analysis of the putative RHTs sequences was performed using the PhyML 3.0 platform, employing the maximum likelihood method with branch support based on the aLRT SH-like approximation. The resulting phylogenetic trees were compared to the taxonomic classification of the analyzed fungal species to identify congruent patterns that support the evolutionary relationships of the candidate RHTs. Several clades with high evolutionary relatedness were identified in both analyses and were highlighted using colored boxes to facilitate interpretation.

**Figure 1:**
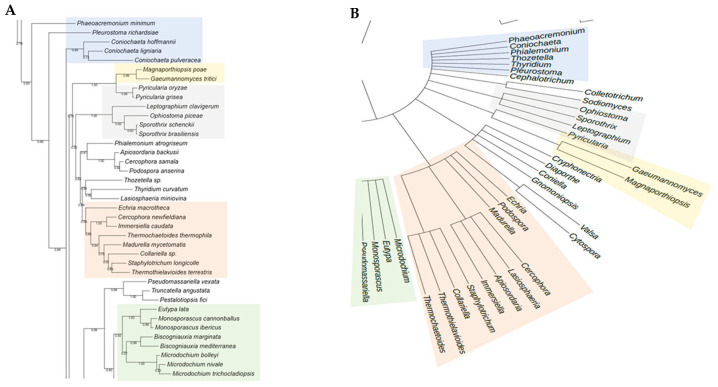
Comparison between the phylogenetic distribution of Rht1 (**A**) and the corresponding taxonomic tree (**B**). Areas highlighted with the same color indicate similarities in the grouping of genera across both trees.

For Rht1, similarities were observed between the phylogeny and taxonomic classification. For instance, species of the genus *Sporothrix* are grouped into a clade alongside representatives of the genera *Ophiostoma* and *Pycularia* (Blue Box), which is consistent with previous studies indicating a phylogenetic relationship between *Sporothrix* and *Ophiostoma*, both members of the Ophiostomataceae family. Nearby, *Magnaporthiopsis* and *Gaeumannomyces* (Yellow Box) also clustered, consistent with their shared membership in the order Magnaporthales. In another clade (Green Box), the genera *Eutypa*, *Biscogniauxia*, *Monosporascus*, and *Microdochium* exhibited similar distribution patterns in both trees. The placement of *Echiria*, *Immersiella*, *Thermothielavioides*, and *Madurella* within the phylogenetic tree (Orange Box) indicated that these genera generally cluster within the same clade, despite minor internal rearrangements. Additionally, the genera *Phaeoacremonium* and *Coniochaeta* showed consistent groupings in the phylogenetic and taxonomic trees, suggesting congruence between their genetic evolution and taxonomic classification. A comparative view of the Rht1 phylogenetic (A) and taxonomic (B) trees is presented in Figure 1.

**Figure 2:**
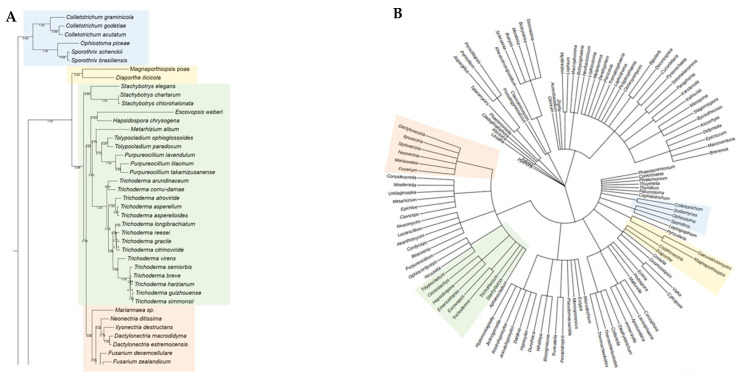
Comparison between the phylogenetic distribution of Rht2 (**A**) and the corresponding taxonomic tree (**B**). Areas highlighted with the same color indicate similarities in the grouping of genera across both trees.

For Rht2, numerous correspondences were observed between phylogeny and taxonomy across various groups. A clear conservation was noted in the clade comprising *Sporothrix*, *Ophiostoma*, and *Colletotrichum* (Blue Box), which clustered similarly in both trees. Likewise, *Magnaporthiopsis* and *Diaporthe* (Yellow Box) retained a consistent organization, in agreement with their classification within the class Sordariomycetes. In the Green Box, *Purpureocillium*, *Tolypocladium*, and *Trichoderma* were grouped similarly in both analyses. Similarly, in the Orange Box, the genera *Neonectria*, *Fusarium*, and *Dactylonectria* formed the same clade in both trees. Overall, the comparison of phylogenetic and taxonomic trees for Rht2 revealed a high degree of concordance, suggesting that the evolutionary history of these sequences aligns with the current taxonomic framework. A side-by-side view of the Rht2 phylogenetic (A) and taxonomic (B) trees is shown in Figure 2.

### 3.3. Identification and Distribution of Conserved Motifs in Putative RHTs

**Figure 3:**
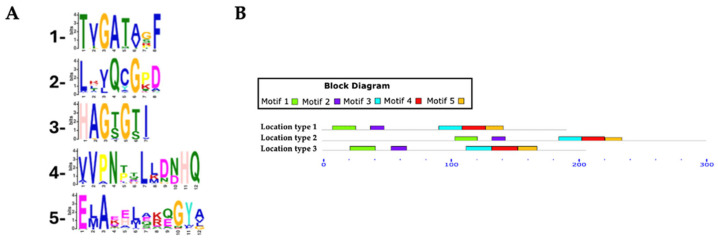
Conserved motifs in Rht1 putative sequences. (**A**) Sequence logos of conserved motifs. (**B**) Distribution patterns of motifs in putative Rht1 sequences according to the MAST map.

The identification and distribution of conserved motifs in putative RHTs were analyzed using MEME Suite, based on protein sequences obtained from HMMER searches (Supplementary Table S2). For the Rht1 motif analysis, 351 protein sequences were analyzed, with lengths ranging from 159 to 720 amino acids and an average length of 233 amino acids. Five conserved motifs were identified in putative Rht1 sequences. Motif 1, TXGATXXF (where “X” represents any amino acid), was detected in 99.4% of the sequences. Motif 2, LXXQXGXX, was found in 100%, while motif 3, HAGXGXI, appeared in 95.4% of sequences. Motif 4, XVPNXXLXXXHQ, was also present in 100%, and motif 5, EXAXXXXXXGYX, in 94.5%. Motif logos are shown in Figure 3A.

Among the identified motifs, motif 1 (TXGATXXF) was the most consistently conserved across all genera analyzed, including *Aspergillus*, *Fusarium*, *Colletotrichum*, *Claviceps*, *Trichoderma*, and *Botrytis*. Motif 3 (HAGXGXI) appeared in all *Fusarium* (54), *Claviceps* (13), and *Diaporthe* (6) sequences, as well as in 7 of 8 *Metarhizium*, 14 of 15 *Trichoderma*, and 44 of 50 *Colletotrichum* species.

Most predicted motifs were found to start between amino acid positions 1 to 20 (*location type 1*), with 311 sequences initiating in this region, representing 88.60% of the total. In contrast, fewer sequences started between positions 50 to 520 (*location type 2*, 7.12%), and only 15 sequences were located between positions 21 to 50 (*location type 3*, 4.27%).

In terms of motif conservation, some sequences lacked specific motifs: 1 sequence did not include motif 1, another lacked motif 3, a 3rd lacked motif 4, and 52 sequences did not contain motif 5.

The three motif location types are shown in Figure 3B, while a detailed list of predicted motifs and their respective starting positions across all putative Rht1 sequences can be found in Supplementary Materials (Table S2 and Figure S1).

**Figure 4:**
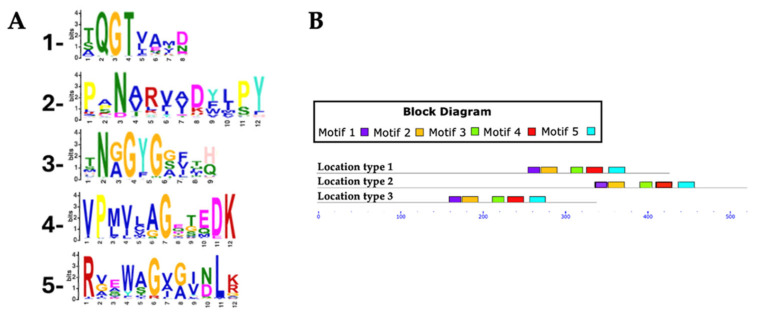
Conserved motifs in Rht2 putative sequences. (**A**) Sequence logos of conserved motifs. (**B**) Distribution patterns of motifs in putative Rht2 sequences according to the MAST map.

For Rht2 motif analysis, a total of 351 protein sequences were analyzed, with lengths ranging from 320 to 695 amino acids and an average length of 458 amino acids. Five conserved motifs were identified in putative Rht2 sequences. Motif 1, XQGTXXXX, was detected in 97.1% of the sequences. Motif 2, XXNXXXXXXXPY, was found in 96%, while motif 3, XNXGYGXXXX, appeared in all sequences. Motif 4, VPXXXXGXXXDK, was present in 97.7%, and motif 5, RXXXXGXXXXLX, in 92.3%. Motif logos are shown in Figure 4A.

Among the motifs identified for Rht2, motif 1 (XQGTXXXX) was the most conserved, being present in all analyzed species of *Diaporthe* (6), *Daldinia* (8), *Botrytis* (10), *Claviceps* (13), *Trichoderma* (15), and *Fusarium* (54), as well as in 7 out of 8 of *Metarhizium* species, 18 of 19 of *Aspergillus* species, and 49 of 50 of *Colletotrichum* species. Meanwhile, motif 3 (XNXGYGXXXX) was found in all protein sequences from *Metarhizium* (8), *Botrytis* (10), *Trichoderma* (15), and *Fusarium* (54), as well as in 12 out of 13 *Claviceps* species, 17 out of 19 *Aspergillus* species, and 46 out of 50 *Colletotrichum* species.

Most predicted motifs started between amino acid positions 240 to 320 (*location type 1*), with 333 sequences falling within this region, representing 94.87% of the total. In smaller proportions, 15 sequences were identified between positions 321 to 441 (*location type 2*, 4.27%), and only 3 sequences between positions 155 to 239 (*location type 3*, 0.85%).

Regarding the conserved motifs, several sequences were found to lack specific motifs: three sequences did not contain motif 1, two sequences lacked motif 3, another two sequences lacked motif 4, and six sequences did not present motif 5.

The different location types mentioned are illustrated in Figure 4B, while the predicted motifs and their respective positions across all Rht2 putative sequences are provided in Supplementary Materials (Table S3 and Figure S2).

### 3.4. Structural Analysis of Predicted RHT Proteins

**Figure 5:**
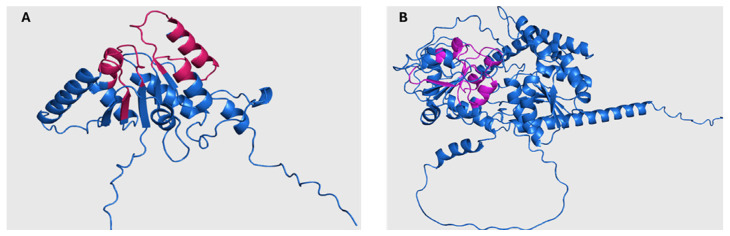
Three-dimensional structure of Rht1 and Rht2 from *S. schenckii.* In (**A**), the predicted structure of Rht1 with conserved motifs is highlighted in magenta. In (**B**), the predicted structure of Rht2 is shown with conserved motifs highlighted in purple.

To explore the structural features of putative RHTs proteins, selected sequences from the conserved motif analysis were used to obtain or model 3D structures in PDB format. When unavailable in the UniProt database, 3D structures were generated using ColabFold v1.5.5: AlphaFold2. Previously reported 3D models of *S. schenckii* Rht1 and Rht2 were used as structural references (Figure 5) [8].

Rht1 of *S. schenckii* displayed a typical Rossmann-like fold (β/α/β motif), consistent with GT-B glycosyltransferases. Rht2, also from *S. schenckii*, showed two Rossmann folds, reinforcing its classification within the GT-B structural family. Using PyMOL, a total of 23 pairwise alignments were performed for each RHT, including species selected based on HMM results and representing both high and low sequence similarity levels. The RMSD values were used to assess structural similarity, with values < 1 considered acceptable [40,41]. These were compared with BLAST-based identity and positive percentages (Table 2 and Table 3).

For Rht1, *S. brasiliensis* showed the highest structural similarity (RMSD = 0.14) and 98% positives, consistent with its close phylogenetic relationship to *S. schenckii*. Other species such as *Fonseca erecta* and *Cordyceps militaris* maintained low RMSD values (1.192 and 1.38, respectively) despite having lower positives (54–56%), indicating structural conservation beyond sequence similarity.

For Rht2, a similar trend was observed. For instance, *Colletotrichum graminicola* (48% identity) showed an RMSD of 0.581, suggesting a conserved fold. On the other hand, the organism with the highest RMSD value analyzed was *Lophiotrema nucula*, with a value of 2.101. However, it is important to note that this organism shares only 33% identity with the model Rht2.

**Figure 6:**
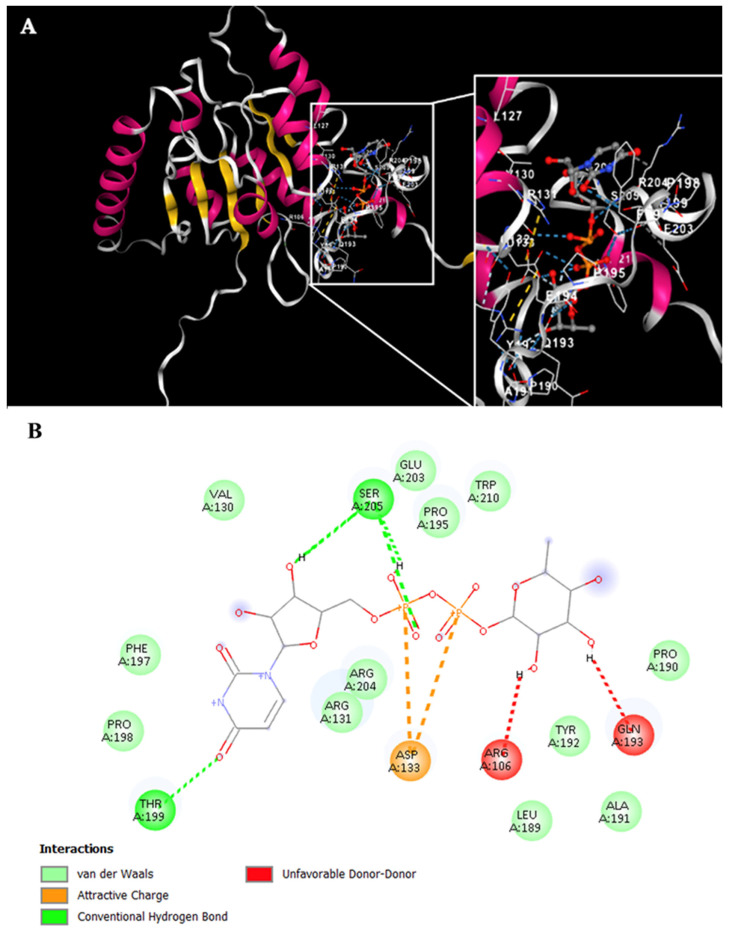
Potential interactions of Rht1 from *S. schenckii* with UDP-L-rhamnose. (**A**) Three-dimensional visualization of the protein–ligand docking complex structure. The white box shows a close-up view of the interaction sites between the protein and the ligand. (**B**) Two-dimensional representation of the same docking complex, showing detailed interactions between the ligand and amino acid residues.

Simultaneously, the Rht1 and Rht2 proteins were analyzed using the CB-Dock tool to investigate their potential interactions with UDP-L-rhamnose. Figure 6A displays the docking results for Rht1 from *S. schenckii*, revealing high-affinity predictions.

Notably, residues near motif 3, HAGSGSI, exhibited a binding interaction between UDP-L-rhamnose and the amino acid residues V130, R131, and D133. Additionally, residues Y192, Q193, F197, P198, T199, E203, R204, and S205 also showed interactions with the molecule (Figure 6B).

**Figure 7:**
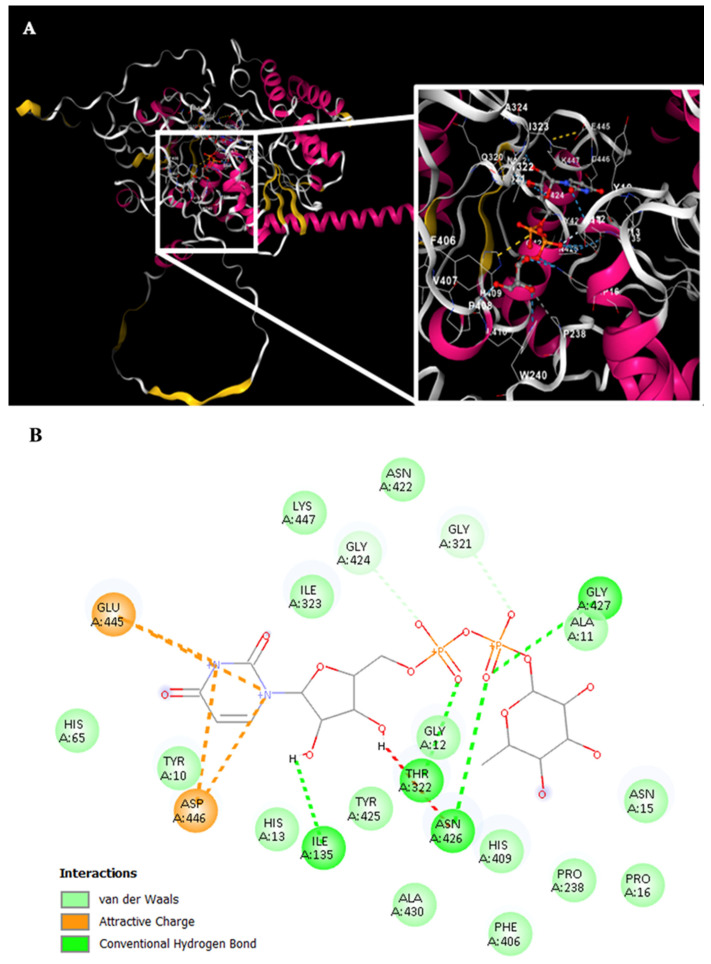
Potential interactions of Rht2 from *S. schenckii* with UDP-L-rhamnose. (**A**) Three-dimensional visualization of the protein–ligand docking complex structure. The white box shows a close-up view of the interaction sites between the protein and the ligand. (**B**) Two-dimensional representation of the same docking complex, showing detailed interactions between the ligand and amino acid residues.

For Rht2 (Figure 7A), the sugar-binding sites included amino acids G321, T322, and I323, corresponding to motif 1 TXGTIA. Additionally, residues N422, G424, Y425, N426, G427, and A430 showed similarity to motif 3 TNAGYNGVXA. Finally, the residues E445, D446, and K447 matched the last four amino acids of motif 4 VPXXXXGXXXDK. Additional interactions with the molecule were observed for residues Y10, A11, G12, H13, N15, P16, I135, P238, F406, and H409 (Figure 7B).

### 3.5. Molecular Docking Analysis of Putative RHTs

Putative Rht1 and Rht2 proteins identified previously were analyzed using Vina Wizard (PyRx–Python Prescription 0.8) to evaluate their affinity for different sugar donors: UDP-L-rhamnose, UDP-glucose, GDP-mannose, and dolichol-phosphate-mannose (Dol-P-mannose). This approach aimed to confirm the specificity of rhamnosyltransferases for UDP-L-rhamnose. Binding affinity, expressed as binding free energy (kcal/mol), was used to assess interaction strength, with more negative values indicating a stronger affinity [31]. For *S. schenckii* Rht1, UDP-L-rhamnose (−7.6 kcal/mol) and GDP-mannose (−7.4 kcal/mol) showed the highest affinities. In contrast, UDP-glucose (−6.0 kcal/mol) and Dol-P-mannose (−6.8 kcal/mol) showed weaker binding. For Rht2, UDP-L-rhamnose (−9.8 kcal/mol) and UDP-mannose (−9.3 kcal/mol) exhibited the strongest affinities, suggesting a substrate preference for these donors. Given the lower affinities for UDP-glucose and Dol-P-mannose, these substrates were excluded from subsequent analyses to focus on those with higher biological relevance.

Table 4 and Table 5 show the molecular docking results for putative Rht1 and Rht2 proteins across various fungal species. In general, most species exhibited a higher binding affinity for UDP-L-rhamnose compared with GDP-mannose, suggesting a preferential interaction with this sugar donor. For instance, *T. reesei* showed a strong preference for UDP-L-rhamnose (−8.9 kcal/mol) over GDP-mannose (−7.9 kcal/mol) in the Rht1 analysis. A similar trend was observed among the putative Rht2 proteins, where *M. anisopliae* showed the highest affinity for UDP-L-rhamnose (−9.7 kcal/mol) relative to GDP-mannose (−8.7 kcal/mol). Likewise, *M. guizhouense* and *T. harzianum* exhibited greater affinities for UDP-L-rhamnose (−9.3 and −9.5 kcal/mol, respectively) than for GDP-mannose (−9.0 and −8.8 kcal/mol, respectively).

### 3.6. In Silico Site-Directed Mutagenesis

Site-directed mutagenesis analyses were performed using the CHARMM-GUI platform [35] to assess the impact of specific amino acid substitutions on the binding affinity of predicted RHTs toward UDP-L-rhamnose.

Initial docking controls with *S. schenckii* Rht1 showed the highest affinity for UDP-L-rhamnose (−8.7 kcal/mol) compared with UDP-glucose (−8.5 kcal/mol) and GDP-mannose (−8.3 kcal/mol), confirming substrate specificity.

Subsequent in silico substitutions in Rht1 identified Y192 as critical for substrate interaction. Its replacement with serine (Y192S) led to a notable reduction in affinity, especially for UDP-L-rhamnose (−8.3 kcal/mol), indicating a change of 0.5 kcal/mol.

This approach was extended to other putative Rht1 proteins. In *Beauveria bassiana*, mutation W115A reduced affinity from −9.1 to −7.9 kcal/mol, while W114A in *Fusarium oxysporum* caused a change of 1.1 kcal/mol (from −8.9 to −7.8 kcal/mol), reducing affinity. Conversely, *Madurella mycetomatis* with mutation W208A showed a minimal change (−8.2 to −8.1 kcal/mol), suggesting a limited role for this residue. In *Fonsecaea pedrosoi*, L121S decreased the binding affinity by 0.9 kcal/mol (from −8.6 to −7.7 kcal/mol), potentially indicating a stabilizing role in the ligand interaction.

Docking controls for the Rht2 protein revealed the highest binding affinity for UDP-L-rhamnose (−9.3 kcal/mol), followed by GDP-mannose (−8.9 kcal/mol) and UDP-glucose (−8.2 kcal/mol). Substitution mutations were performed, and the most significant effects were observed with the double mutation H13S/D446A in *S. schenckii*, which reduced the binding affinity to UDP-L-rhamnose to −8.0 kcal/mol, representing a change of 1.3 kcal/mol. A moderate reduction was also observed for GDP-mannose (−8.4 kcal/mol, change of 0.5 kcal/mol), while affinity for UDP-glucose increased to −8.9 kcal/mol (change of 0.7 kcal/mol).

Among all evaluated species, *S. schenckii* exhibited the greatest reduction in ligand binding mutation, suggesting that H13 and D446 play critical roles in substrate interaction. Additionally, *Ophiostoma piceae* and *Xylona heveae* showed the highest wild-type affinities (−10.0 kcal/mol), with changes of 0.8 and 0.7 kcal/mol, respectively, upon mutation. In contrast, *T. reesei* lacked an orthologous residue at the position corresponding to H13 in *S. schenckii*, so alternative residues involved in UDP-L-rhamnose binding were targeted. Mutants H302S and P221A showed minimal changes in affinity, with only a 0.1 kcal/mol change (from −8.9 to −8.8 kcal/mol), suggesting these residues are not key to substrate interaction.

In *Macrophomina phaseolina*, the H19S/D378A mutation resulted in a 0.5 kcal/mol reduction in binding affinity (from −9.5 to −9.0 kcal/mol), while in *Magnaporthiopsis poae* (H31S/D383A) the decrease was smaller (0.2 kcal/mol), indicating greater tolerance to substitutions at these positions.

These findings highlight the importance of specific residues in maintaining the stability of protein–ligand interactions and provide valuable insights for the future structural and functional optimization of these enzymes. Structural similarity between wild-type and mutant proteins was assessed using RMSD values to confirm that the observed affinity changes were attributable to residue substitution rather than major conformational alterations. Results are detailed in Table 6 and Table 7.

### 3.7. Cell Wall Carbohydrate Composition in Species with Putative RHTs

To assess the presence of rhamnose in some species with putative Rht1 and Rht2 proteins, the carbohydrate composition of the cell wall was analyzed. Quantified carbohydrates included rhamnose, glucosamine, glucose, mannose, and galactose. Values were normalized to represent relative percentages, adding to 100%.

**Figure 8:**
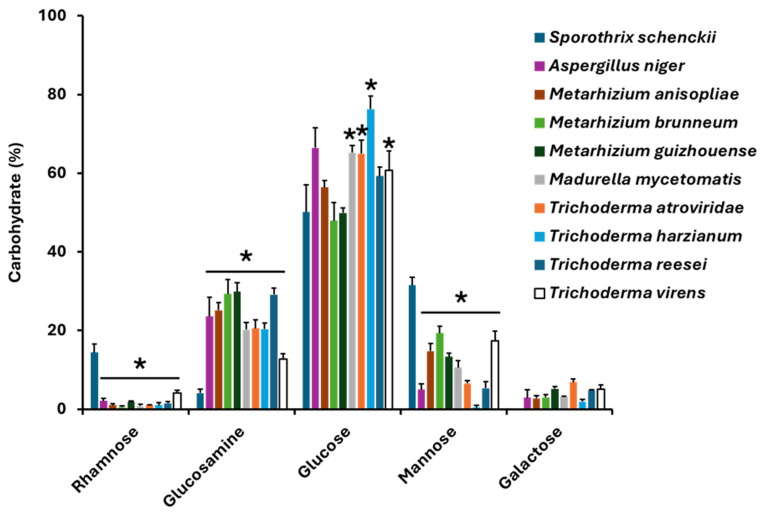
Cell wall carbohydrate composition in species with putative RHTs. Error bars represent the mean ± SD from three biological replicates per condition. * *p* < 0.05 when compared with *Sporothrix schenckii*.

Glucose was the predominant sugar, ranging from 47.91% to 76.33%, with *M. guizhouense* showing the highest content. When compared with the *S. schenckii* cell wall, *M. mycetomatis*. *T. atroviridae*, *T. harzianum*, and *T. virens* showed higher glucose levels (Figure 8). Mannose exhibited high variability (0.46% to 31.5%), being most abundant in *S. schenckii* (Figure 8). On the contrary, this fungal species showed the lowest level of glucosamine, and no galactose was detected (Figure 8). Notably, rhamnose—a sugar previously reported only in *S. schenckii*—was detected in all analyzed species, reaching up to 4.07% in *T. virens*. Full results are presented in Figure 8.

### 3.8. Enzymatic Analysis of Putative RHTs

To determine whether the predicted species exhibited any RHT activity, we measured the enzyme activity in cell homogenates, with α-1,6-mannobiose as the rhamnose acceptor and UDP-L-rhamnose as the donor. Reaction products were analyzed by HPAEC-PAD, and results were expressed as trisaccharide min^−1^ per mg protein^−1^.

The highest enzymatic activity was observed in *S. schenckii* (123.63 ± 18.46 trisaccharide min^−1^ mg protein^−1^), consistent with the prior knowledge of its ability to utilize UDP-L-rhamnose. In contrast, negative controls *Candida albicans* and *Saccharomyces cerevisiae* exhibited near-zero activity, confirming the absence of RHT activity in these species. Statistical analysis revealed no significant difference between them, further reinforcing the lack of detectable rhamnosyltransferase function. Intermediate activity levels were detected in *Aspergillus niger* (59.47 ± 3.91 trisaccharide min^−1^ mg protein^−1^), *Trichoderma virens* (68.03 ± 10.31 trisaccharide min^−1^ mg protein^−1^), and *Trichoderma reesei* (39.20 ± 7.15 trisaccharide min^−1^ mg protein^−1^), suggesting rhamnose transfer function in these organisms. The low activity observed in the no acceptor condition supports the enzymatic specificity, and residual values with inactivated protein confirm the association with RHT processes. The enzymatic analysis results are summarized in Table 8.

## 4. Discussion

Using HMM profiles, we identified putative Rht1 and Rht2 sequences from the fungal portion of the NCBI NR database. This approach is effective for detecting distant orthologs, though its success depends on the completeness and annotation quality of genomic data [42]. The limited representation of certain fungal groups likely reflects the under-sequencing of these taxa.

Our results revealed a broad taxonomic distribution of RHTs, especially in ecologically and biotechnologically relevant fungi. Rht1 was most frequent in *Aspergillus*, *Penicillium*, and *Fusarium*, while Rht2 predominated in *Fusarium* and *Colletotrichum*. These genera are known for pathogenesis and secondary metabolite production, with some species acting as plant pathogens and others as producers of industrial enzymes or mycotoxins [43,44,45,46,47,48,49,50,51,52].

The co-occurrence of Rht1 and Rht2 in 126 genera suggests complementary metabolic functions. Notably, both RHTs were found in endophytic and saprophytic fungi like *Trichoderma*, *Claviceps*, and *Xylaria*, indicating roles beyond pathogenesis [53,54,55,56,57].

From an evolutionary perspective, the presence of Rht1 and Rht2 in diverse taxonomic lineages suggests a functional distribution of these enzymes within the fungal kingdom, particularly in the phylum *Ascomycota*. However, they appear to be absent in other phyla, such as *Basidiomycota*. Previous studies analyzing members of *Basidiomycota* have shown that their cell walls are primarily composed of glucose, mannose, and galactose, with an occasional presence of xylose and fucose but a consistently low or absent content of rhamnose, suggesting ecological and functional differences [58]. These compositional differences likely reflect the following distinct ecological strategies: Basidiomycetes are specialized in lignocellulose degradation [59], whereas Ascomycetes, which often colonize rhamnose-rich plant tissues, may be associated with their direct interactions with plant hosts in survival, saprophytic, mutualistic, or pathogenic contexts [52,60,61,62,63].

The evolutionary origin of RHTs may involve vertical inheritance, horizontal gene transfer, or gene loss in certain lineages. Alternatively, these enzymes may have originated within the fungal kingdom, with Basidiomycota either losing or never utilizing rhamnose and RHTs, instead adapting their cell wall and glycoconjugate structures to other substrates typical of their environment [64].

To explore the evolutionary history and diversification of RHTs, we compared the phylogenetic and taxonomic relationships among fungi with putative Rht1 and Rht2. In the case of Rht1, the clustering of taxonomically distant genera, such as *Pyricularia* and *Sporothrix*, despite their ecological differences, suggests the potential structural or functional conservation of RHTs, possibly related to polysaccharide biosynthesis or host/environmental adaptation [8,65]. Similarly, the grouping of phytopathogens like *Magnaporthiopsis* and *Monosporascus* may reflect a convergent evolution driven by plant-associated lifestyles [66,67]. For Rht2, phylogenetic and taxonomic trees reveal a notable congruence in the clustering of fungal genera. Genera ranging from phytopathogens and entomopathogens to biocontrol agents highlight a potentially broad adaptive role for Rht2, possibly involving the regulation of cell wall architecture and participation in host or environmental interaction mechanisms. For instance, enzymatic activities associated with host invasion or decomposition, such as rhamnose-dependent pectin degradation in *Colletotrichum* [68], and substrate colonization traits in *Trichoderma* and *Purpureocillium*, may reflect conserved Rht2 functions supporting ecological adaptation [52,54]. In addition to these cases, other genera distributed across diverse taxonomic orders were also identified, suggesting possible retention or functional convergence of RHTs across Ascomycota lineages [69,70,71,72,73].

These findings provide valuable insight into the evolutionary relationships among RHT-containing fungi and support the hypothesis that these enzymes share conserved sequence motifs, alongside taxon-specific variations associated with functional diversification within the fungal kingdom. The high degree of conservation observed in motifs such as TXGATXXF, LXXQXG, and HAGXGXI in Rht1, and XQGT and XNXGYG in Rht2, suggests that these sequence elements are critical for catalytic activity and/or substrate recognition. Despite the minor variation in motif starting positions among RHT sequences, the spacing between conserved motifs remained consistent. This variation in initial motif positions may suggest differences in the N-terminal regions of these proteins, potentially due to divergence in sequence length or domain architecture across species. Such conservation in inter-motif distances may reflect evolutionary constraints that maintain the integrity of the active site and support a conserved enzymatic function across species. The absence of motif 5 (Rht1) in a larger number of sequences could indicate functional divergence or a loss of secondary features that are not essential for the primary enzymatic activity. This suggests that the catalytic region is structurally preserved across sequences, reinforcing the idea of a conserved functional domain even amid sequence diversity. Notably, these motifs showed no similarity to previously characterized canonical glycosyltransferases. While GT-A-type glycosyltransferases typically exhibit the conserved Asp-X-Asp (DXD) motif implicated in divalent cation coordination and nucleotide-sugar stabilization, this feature was not observed in the RHT sequences examined. Although conserved motifs like DXD are common in glycosyltransferases, they are not universal or strictly indicative of function, as motif structure and context can vary widely among families, reflecting their functional and structural diversity [74,75,76].

The analysis of Rht1 and Rht2 sequences reveals substantial conservation among phylogenetically related species, suggesting functional preservation across divergent lineages. In the case of Rht1, structural alignments with more distantly related species, such as *Fusarium erecta* and *Cordyceps militaris*, showed moderate sequence identities (54–56%) but retained low RMSD scores. Similarly, for Rht2, the alignment between *C. graminicola* and *S. schenckii* yielded an RMSD of 0.581 despite a low sequence identity. These results suggest that essential structural elements, particularly those involved in substrate binding or catalytic site stabilization, may be conserved even when amino acid sequences differ significantly [75,76,77]. Collectively, these findings are in line with previous reports emphasizing the evolutionary plasticity of glycosyltransferase sequences alongside the conservation of structurally and functionally critical domains [76]. The observed structural conservation among both closely related and taxonomically distant fungal species suggests that RHTs may have undergone evolutionary adaptation to diverse ecological niches while retaining their essential biological roles.

To assess the functional relevance of the observed structural similarities, molecular docking analysis was performed between the putative RHTs and UDP-L-rhamnose. In *S. schenckii*, both Rht1 and Rht2 exhibited the highest affinity for UDP-L-rhamnose, consistent with previous studies reporting enzymatic interaction with this substrate [8]. In contrast, GDP-mannose, UDP-glucose, and Dol-P-mannose showed lower binding affinities, suggesting fewer stable interactions and limited functional relevance. Most putative Rht1 and Rht2 proteins exhibited a consistent preference for UDP-L-rhamnose over GDP-mannose, indicating the specificity of these RHTs for UDP-L-rhamnose as a substrate. Moreover, the co-localization of predicted motifs with substrate-binding sites supports the potential role of these proteins as functional RHTs.

In silico site-directed mutagenesis was conducted to explore structural determinants of substrate specificity in putative RHTs. Targeted amino acid substitutions in Rht1, such as Y192S in *S. schenckii* and W115A in *B. bassiana*, led to measurable reductions in the binding affinity to UDP-L-rhamnose, highlighting the functional importance of these residues. In Rht2, mutations H13S and D446A induced an even greater loss of affinity, suggesting that these residues are particularly critical for substrate stabilization.

Interestingly, the affected residues in fungal RHTs correspond to key catalytic residues previously identified in plant RHTs. For example, H22 and D121 in UGT71G1 (*Medicago truncatula*), H20 and D119 in VvGT1 (*Vitis vinifera*), and H21 and S124 in UGT89C1 (*A. thaliana*) are known to be part of the catalytic site that directly interacts with the UDP -L-rhamnose [78,79]. Similarly, in MrUGT78R1 from *Morella rubra*, mutations at D406 completely abolished RHT activity [80].

The parallels between the plant and fungal enzymes suggest that the catalytic core of RHTs is evolutionarily conserved, with histidine and aspartate residues playing central roles in substrate coordination and catalysis. To confirm the presence of RHTs in the organisms previously identified as harboring putative RHTs, in silico and in vitro approaches were combined. For this purpose, the cell wall carbohydrate composition was characterized, and enzymatic activity assays were performed to evaluate whether the predicted presence of RHT-like sequences correlates with measurable RHTs activity, thus providing functional support for the computational findings. Glucose was identified as the main component of the fungal cell wall, consistent with its well-established structural role in fungi [81]. Mannose content showed high variability among species (0.46–19.3%), with the highest levels observed in *M. brunneum*. Mannose is typically associated with glycoproteins and mannans involved in cell adhesion and environmental interactions [82].

Rhamnose was detected in all species analyzed. The presence of this sugar in the cell walls of fungal species identified as potential RHT carriers supports the proposition that these enzymes may be actively involved in rhamnosylation processes. However, this finding also raises new questions regarding the specific role of rhamnose in the structure and dynamics of the fungal cell wall, as well as the identity of the polysaccharides in which it may be incorporated. Beyond the species analyzed in this study, rhamnose has also been reported in other fungal genera, such as *Rhynchosporium secalis*, *Penicillium chrysogenum*, and in the spore mucilage of *C. graminicola*, which contains glycoproteins with rhamnose [52,83,84]. Additionally, functional genes involved in UDP-L-rhamnose biosynthesis, such as UG4,6-Dh and U4k6dG-ER, have been identified in *Magnaporthe grisea* and *B. cinerea* [85].

These findings support the idea that rhamnosylation is a conserved process in fungal lineages, potentially mediated by the putative RHTs identified in this study. Differences in other sugar components further highlight species-specific variations in cell wall architecture and functionality.

The detection of rhamnose in the cell walls of all analyzed species, along with the observed rhamnose transfer activity using UDP-L-rhamnose as donor and α-1,6-mannobiose as acceptor, provides functional evidence for the role of these enzymes. Notably, *S. schenckii* exhibited the highest transfer rate, consistent with previously reported use of UDP-L-rhamnose [8], while other species such as *A. niger*, *T. virens*, and *T. reesei* showed intermediate RHT activity levels, suggesting the presence of functional RHTs with varying degrees of activity.

In contrast, the absence of RHT activity in *C. albicans* and *S. cerevisiae*, both of which lack rhamnose in their cell walls [86], reinforces the specificity of the enzymatic process and suggests that rhamnose incorporation is restricted to certain fungal lineages. Together, cell wall composition and enzymatic activity analyses support a model in which rhamnose incorporation into the fungal cell wall is mediated by RHTs. The variability in activity levels suggests potential differences in enzyme regulation or precursor availability across species.

## 5. Conclusions

Fungal cell walls contain diverse glycoconjugates, yet the enzymes involved in rhamnose incorporation—such as RHTs—remain poorly characterized. Due to the limited knowledge of their evolutionary origin and distribution, this study aimed to identify and characterize putative RHTs across the fungal kingdom. Our results highlighted the evolutionary and functional relevance of RHTs in fungi by integrating computational, phylogenetic, structural, and biochemical approaches. The application of HMM enabled the sensitive detection of putative RHTs across diverse fungal taxa, revealing a heterogeneous but phylogenetically enriched distribution, particularly in Ascomycota, which suggests an evolutionary trajectory of functional specialization. Conserved sequence motifs identified in putative RHTs, and their proximity to residues predicted to interact with the UDP-L-rhamnose substrate in molecular docking analyses, support the existence of lineage-specific functional adaptations. These findings support a model in which rhamnose incorporation into the fungal cell wall is a conserved but restricted process, mediated by specialized RHTs in fungal lineages. Future research should focus on the experimental validation of these putative RHTs, including biochemical characterization and gene disruption studies, to confirm their roles in cell wall biosynthesis and fungal physiology.

## Figures and Tables

**Table 1:** Selected fungal species and accession numbers for putative RHTs.

Species	Rht1 Accession *	Rht2 Accession *
*Aspergillus niger*	EHA26758.1	GKZ64237.1
*Botrytis cinerea*	XP_001557717.1	EMR81961.1
*Claviceps purpurea*	KAG6139429.1	KAG6132685.1
*Colletotrichum graminicola*	XP_008092350.1	XP_008100482.1
*Cordyceps militaris*	ATY63072.1	XP_006674754.1
*Diaporthe eres*	KAI7784925.1	KAI7784403.1
*Fusarium oxysporum*	RKK72986.1	KAJ4047383.1
*Metarhizium anisopliae*	KJK84664.1	KJK80520.1
*Metarhizium brunneum*	XP_014545532.1	XP_014548452.1
*Metarhizium guizhouense*	KID87172.1	KID92007.1
*Metarhizium robertsii*	XP_007819142.2	XP_007822448.1
*Madurella mycetomatis*	KXX75366.1	KXX79238.1
*Sporothrix brasiliensis*	XP_040616120.1	XP_040617365.1
*Sporothrix schenckii*	XP_016583713.1	XP_016584143.1
*Trichoderma atroviride*	XP_013943227.1	UKZ67550.1
*Trichoderma guizhouense*	OPB40233.1	OPB36448.1
*Trichoderma harzianum*	XP_024772549.1	KKO97596.1
*Trichoderma reesei*	XP_006961478.1	XP_006968899.1
*Trichoderma virens*	XP_013961374.1	XP_013957984.1

* A unique identifier assigned to each biological sequence within the NCBI database.

**Table 2:** Structural alignments between Rht1 from *Sporothrix schenckii* and selected fungal species using PyMOL.

Species	Accession Number	Residues	RMSD	Identities (%)	Positives (%)
*Sporothrix brasiliensis*	XP040616120.1	159	0.14	97	98
*Ophiostoma piceae*	EPE10043.1	162	0.576	64	74
*Podospora anserina*	XP 001903917.1	129	0.65	40	49
*Grosmannia clavigera*	XP_014168710	156	0.677	54	66
*Ascodesmis nigricans*	TGZ82940.1	95	0.678	37	50
*Madurella mycetomatis*	KXX75366.1	155	0.729	45	56
*Thermothelomyces thermophilus*	XP_003664509.1	158	0.73	42	54
*Podospora comata*	VBB80562.1	135	0.75	43	55
*Penicillium digitatum*	XP_014534696.1	92	0.752	37	52
*Thermochaetoides thermophila*	XP_006694985.1	144	0.795	38	50
*Trichoderma reesei*	XP_006961478.1	112	0.795	38	55
*Metarhizium guizhouense*	KID87172.1	124	0.836	38	52
*Aspergillus niger*	EHA26758.1	98	0.84	40	54
*Paracoccidioides brasiliensis*	XP_010757480.1	113	0.872	35	55
*Amniculicola lignicola*	KAF2003674.1	105	0.964	38	53
*Coccidioides immitis*	XP_001246780.1	122	0.978	43	55
*Thermothielavioides terrestris*	SPQ26596.1	156	1.013	41	52
*Macrophomina phaseolina*	EKG10336.1	119	1.068	35	54
*Fonsecaea pedrosoi*	XP_013280197.1	123	1.074	36	55
*Fonsecaea multimorphosa*	XP_016629425.1	127	1.185	36	53
*Fonsecaea erecta*	XP_018698961.1	126	1.192	35	54
*Cordyceps militaris*	ATY63072.1	127	1.38	42	56
*Friedmanniomyces simplex*	TKA63572.1	115	1.468	37	49

Accession Number: Unique identifier assigned to each biological sequence within the NCBI database. Residues: The number of residues involved in the three-dimensional structure of the protein from each analyzed species. RMSD: The root means square deviation, which measures the average difference between the positions of atoms in two aligned structures, indicating the degree of structural similarity. Identity (%): Percentage of exact matches between the amino acid sequences of the aligned proteins. Positives (%): Percentage of amino acid residues in the aligned proteins that are similar (though not necessarily identical), calculated based on sequence comparison.

**Table 3:** Three-dimensional structure alignments between Rht2 from *Sporothrix schenckii* and selected fungal species using PyMOL.

Species	Accession Number	Residues	RMSD	Identity (%)	Positives (%)
*Colletotrichum graminicola*	XP_008100482.1	323	0.581	48	54
*Ophiostoma piceae*	EPE10437.1	375	0.605	57	65
*Calocera cornea*	KZT50863.1	345	0.91	39	48
*Grosmannia clavigera*	XP_014175810.1	252	0.965	26	38
*Hyaloscypha hepaticicola*	PMD21084.1	263	0.972	35	49
*Macrophomina phaseolina*	EKG11414.1	263	1.056	26	41
*Marssonina coronariae*	OWP01936.1	272	1.109	26	41
*Madurella mycetomatis*	KXX79238.1	268	1.115	24	37
*Orbilia oligospora*	KAF3191956.1	288	1.131	33	49
*Pyrenochaeta* sp.	OAL46984.1	237	1.157	22	39
*Lasallia pustulata*	KAA6413478.1	301	1.16	28	43
*Phaeomoniella chlamydospora*	KKY20074.1	275	1.222	26	40
*Phialocephala subalpina*	CZR67232.1	259	1.249	30	47
*Podospora anserina*	XP_001903477.1	218	1.308	32	48
*Arthrobotrys flagrans*	RVD83950.1	297	1.316	33	48
*Zopfia rhizophila*	KAF2191997.1	238	1.361	27	39
*Decorospora gaudefroyi*	KAF1836052.1	248	1.462	28	41
*Thermothielavioides terrestris*	XP_003658264.1	280	1.469	25	37
*Viridothelium virens*	KAF2235942.1	288	1.497	27	39
*Dactylellina haptotyla*	EPS37979.1	289	1.54	32	48
*Mytilinidion resinicola*	XP_033568734.1	273	1.551	34	49
*Cladophialophora carrionii*	XP_008725802.1	275	2.044	26	38
*Lophiotrema nucula*	KAF2113514.1	257	2.101	33	54

Accession Number: Unique identifier assigned to each biological sequence within the NCBI database. Residues: The number of residues involved in the three-dimensional structure of the protein from each analyzed species. RMSD: The root means square deviation, which measures the average difference between the positions of atoms in two aligned structures, indicating the degree of structural similarity. Identity (%): Percentage of exact matches between the amino acid sequences of the aligned proteins. Positives (%): Percentage of amino acid residues in the aligned proteins that are similar (though not necessarily identical), calculated based on sequence comparison.

**Table 4:** Molecular docking analysis between species with putative Rht1 proteins.

Species	Accession Number	Binding Affinity (Kcal/mol)	
		UDP-L-Rhamnose	GDP-Mannose
*Sporothrix schenckii*	XP_016583713.1	−7.6	−7.4
*Aspergillus niger*	EHA26758.1	−7.7	−7.6
*Metarhizium anisopliae*	KJK84664.1	−7.1	−7.1
*Metarhizium guizhouense*	KID87172.1	−8.3	−7.4
*Trichoderma atroviride*	XP_013943227.1	−7.3	−7.2
*Trichoderma reesei*	XP_006961478.1	−8.9	−7.9
*Trichoderma virens*	XP_013961374.1	−7.5	−7.2

Accession Number: Unique identifier assigned to each biological sequence within the NCBI database. The columns “UDP-L-Rhamnose” and “GDP-Mannose” show the binding affinities between each protein and these specific ligands, expressed in kilocalories per mole (kcal/mol).

**Table 5:** Molecular docking analysis between species with putative Rht2 proteins.

Species	Accession Number	Binding Affinity (Kcal/mol)	
		UDP-L-Rhamnose	GDP-Mannose
*Sporothrix schenckii*	XP_016584143.1	−9.8	−9.3
*Aspergillus niger*	GKZ64237.1	−7.7	−7.6
*Metarhizium anisopliae*	KJK80520.1	−9.7	−8.7
*Metarhizium guizhouense*	KID92007.1	−9.3	−9
*Trichoderma atroviride*	UKZ67550.1	−7.3	−7.1
*Trichoderma harzianum*	KKO97596.1	−9.5	−8.8
*Trichoderma reesei*	XP_006968899.1	−8.8	−8.3
*Trichoderma virens*	XP_013957984.1	−8.3	−7.4

Accession Number: Unique identifier assigned to each biological sequence within the NCBI database. The columns “UDP-L-Rhamnose” and “GDP-Mannose” show the binding affinities between each protein and these specific ligands, expressed in kilocalories per mole (kcal/mol).

**Table 6:** In silico site-directed mutagenesis of putative Rht1 proteins.

Species	Mutated Residue	Binding Affinity (Kcal/mol)		RMSD
		WT	PM	
*Sporothrix schenckii*	Y192S	−8.7	−8.3	0.00 (232 a.a)
*Aspergillus niger*	L122A	−7.5	−7.3	0.00 (159 a.a)
*Metarhizium brunneum*	W116A	−9.0	−8.5	0.00 (211 a.a)
*Trichoderma reesei*	W112A	−8.6	−8.0	0.00 (201 a.a)
*Trichoderma atroviride*	W112A	−8.3	−7.9	0.00 (207 a.a)
*Trichoderma virens*	W112A	−8.3	−7.8	0.00 (207 a.a)
*Madurella mycetomatis*	W208A	−8.2	−8.1	0.00 (331 a.a)
*Fonsecaea pedrosoi*	L121S	−8.6	−7.7	0.00 (205 a.a)
*Neonectria ditissima*	W114A	−8.9	−8.4	0.00 (208 a.a)
*Beauveria bassiana*	W115A	−9.1	−7.9	0.00 (209 a.a)
*Fusarium oxysporum*	W114A	−8.9	−7.8	0.00 (208 a.a)

a.a. MUT—mutated amino acids; WT—wild-type protein; PM—mutated protein; RMSD—alignment between the wild-type and the mutated protein.

**Table 7:** In silico site-directed mutagenesis of putative Rht2 proteins.

Species	Mutated Residue	Binding Affinity (Kcal/mol)		RMSD
		WT	PM	
*Sporothrix schenckii*	H13S, D446A	−9.3	−8.0	0.00 (576 a.a)
*Ophiostoma piceae*	G135S, D563A	−10.0	−9.2	0.00 (695 a.a)
*Xylona heveae*	H15S, D386A	−10.0	−9.3	0.00 (461 a.a)
*Magnaporthiopsis poae*	H31S, D383A	−9.5	−9.3	0.00 (461 a.a)
*Botrytis cinerea*	H19S, E379A	−8.7	−8.2	0.00 (452 a.a)
*Macrophomina phaseolina*	H19S, D378A	−9.5	−9.0	0.00 (449 a.a)
*Aspergillus niger*	H29E, T298F	−9.6	−9.3	0.00 (456 a.a)
*Madurella mycetomatis*	L250S	−9.4	−9.2	0.00 (452 a.a)
*Metarhizium brunneum*	P34G, M242G	−9.5	−9.2	0.00 (457 a.a)
*Trichoderma ressei*	H302S, P221A	−8.9	−8.8	0.00 (418 a.a)

a.a. MUT—mutated amino acids; WT—wild-type protein; PM—mutated protein; RMSD—alignment between the wild-type and the mutated protein.

**Table 8:** Enzymatic analysis of putative RHTs.

Species	UDP-Rhamnose	Without Acceptor	UDP-L-Rhamnose and Inactivated Protein
*Sporothrix schenckii*	123.63 ± 18.46	1.40 ± 0.53	0.08 ± 0.03
*Candida albicans*	0.12 ± 0.08 *	0.17 ± 0.06 *	0.02 ± 0.03
*Saccharomyces cerevisiae*	0.17 ± 0.12 *	0.05 ± 0.05 *	0.02 ± 0.03
*Aspergillus niger*	59.47 ± 3.91 * ^†^	0.87 ± 0.25	0.15 ± 0.09
*Madurella mycetomatis*	37.47 ± 6.14 *	0.93 ± 0.42	0.10 ± 0.00
*Metarhizium anisopliae*	35.77 ± 7.07 *	1.27 ± 0.35	0.13 ± 0.12
*Metarhizium brunneum*	28.40 ± 7.37 *	1.37 ± 0.61	0.17 ± 0.06
*Metarhizium guizhouense*	29.23 ± 6.65 *	0.40 ± 0.20 *	0.12 ± 0.08
*Trichoderma atroviride*	30.93 ± 6.81 *	0.80 ± 0.35	0.07 ± 0.06
*Trichoderma harzianum*	31.70 ± 7.88 *	0.63 ± 0.31	0.07 ± 0.12
*Trichoderma reesei*	39.20 ±7.15 *	0.63 ± 0.23	0.08 ± 0.10
*Trichoderma virens*	68.03 ± 10.31 * ^‡^	1.10 ± 0.44	0.10 ± 0.10

Expressed as trisaccharide min^−1^ per mg protein^−1^. Results express the mean ± SD, n = 3. * *p* < 0.05 when compared with *Sporothrix schenckii*. ^† ^*p* < 0.05 when compared with all the analyzed species, except *Trichoderma virens*. ^‡ ^*p* < 0.05 when compared with all the analyzed species, except *Aspergillus niger.*

## Data Availability

The original contributions presented in this study are included in the article/supplementary material. Further inquiries can be directed to the corresponding authors.

## Supplementary Materials

The following supporting information can be downloaded at: https://www.mdpi.com/article/10.3390/jof11070524/s1. Table S1: Genera, species, and accession numbers for putative RHTs. Table S2: Identified motifs in putative Rht1. Figure S1: Motifs location types in putative Rht1 sequences. Table S3: Identified motifs in putative Rht2. Figure S2: Motifs location types in putative Rht2 sequences.
