# Supplementary material for: Evolutionary History and Distribution Analysis of Rhamnosyltransferases in the Fungal Kingdom
Source: J Fungi (Basel). 2025 Jul 15;11(7):524. doi: 10.3390/jof11070524 (PMC12295706; doi:10.3390/jof11070524)
Supplement: Supplementary file 1 [file jof-11-00524-s001.zip › jof-3683894-supplementary.pdf]

Supplementary material

Evolutionary history and distribution analysis of rhamnosyltransferases in the fungal kingdom

Joaquín O. Chávez-Santiago[1], Luz A. López-Ramírez [1], Luis A. Pérez-García[2], Iván Martínez-Duncker [3], Bernardo Franco [1], Israel E. Padilla-Guerrero [1], Vianey Olmedo-Monfil [1], J Félix Gutiérrez-Corona [1], Gustavo A. Niño-Vega [1], Jorge H. Ramírez-Prado [4*] and Héctor M. Mora-Montes [1*]

1. Departamento de Biología, División de Ciencias Naturales y Exactas, Universidad de Guanajuato, Guanajuato, Gto., 36050, Mexico.
2. Facultad de Estudios Profesionales Zona Huasteca, Universidad Autónoma de San Luis Potosí, Romualdo del Campo 501, Fracc. Rafael Curiel, 79060, Ciudad Valles, S.L.P., Mexico.
3. Laboratorio de Glicobiología Humana y Diagnóstico Molecular, Centro de Investigación en Dinámica Celular, Instituto de Investigación en Ciencias Básicas y Aplicadas, Universidad Autónoma del Estado de Morelos, Cuernavaca Mor. 62209, México
4. Unidad de Biotecnología, Centro de Investigación Científica de Yucatán, A. C., Calle 43 No. 130, Col. Chuburná de Hidalgo, Mérida, Yucatán 97205, Mexico

* Both authors equally contributed to this work. Correspondence: jhramirez@cicy.mx (J. H. R.-P.) and hmora@ugto.mx (H. M. M.-M.)

Supplementary Bioinformatics Methodologies

2.1 Downloading the NCBI Database

The non-redundant (NR) BLAST database was downloaded on October 9, 2023, directly from the NCBI platform. This database was stored in 82 compressed files in .tar format, accompanied by 82 .md5 files used to verify the integrity of each .tar file. The .md5 files were downloaded separately to confirm the integrity of each downloaded .tar file before obtaining the complete data package. Subsequently, these files were decompressed and stored on the ADATA external hard drive using the Windows Subsystem for Linux (WSL) environment. The command used for downloading was:

```
sudo wget -p /mnt/adata
https://ftp.ncbi.nlm.nih.gov/blast/db/
```

The integrity of the downloads was verified using the corresponding .md5 file with the following command:

```
md5sum filename.txt
```

After verification, the files were decompressed and stored in a folder named "DBNR" within the WSL environment on the external hard drive.

2.2 Identification and Distribution of conserved Motifs in putative RHTs

A detailed list of the predicted motifs and their respective starting positions across all putative *Rht1* and *Rht2* sequences is provided in Supplementary Materials.

Table S1. Genera, species, and accession numbers for putative RHTs.

Genera	Species	Rht1 accession	Rht2 accession
Akanthomyces	*Akanthomyces lecanii*	OAA77240.1	OAA70212.1
	Akanthomyces muscarius	XP_056049605.1	XP_056054863.1
Alectoria	*Alectoria fallacina*	CAF9938699.1	CAF9943470.1
	Alectoria sarmentosa	CAD6567071.1	CAD6566374.1
Annulohypoxylon	*Annulohypoxylon bovei*	KAI2473205.1	KAI2463407.1
	Annulohypoxylon moriforme	KAI1454471.1	KAI1454171.1
	Annulohypoxylon nitens	KAI0892314.1	KAI0896334.1
	Annulohypoxylon stygium	KAI1446163.1	KAI1445084.1
	Annulohypoxylon truncatum	XP_047856260.1	XP_047849137.1
Aphanocladium	*Aphanocladium album*	KAJ6785788.1	KAJ6789860.1
Apiosordaria	*Apiosordaria backusii*	KAK0701482.1	KAK0718985.1
Ascochyta	*Ascochyta clinopodiicola*	KAJ4351106.1	KAJ4346241.1
	Ascochyta lentis	KAF9700101.1	KAF9695519.1
	Ascochyta rabiei	XP_059492683.1	XP_038797369.2
Aspergillus	*Aspergillus awamori*	GCB26403.1	GCB23152.1
	Aspergillus brasiliensis	OJJ74616.1	GKZ22376.1
	Aspergillus carbonarius	OOF95088.1	OOG00364.1
	Aspergillus carlsbadensis	KAJ0426393.1	KAJ0415219.1
	Aspergillus costaricensis	XP_025534806.1	XP_025540426.1
	Aspergillus eucalypticola	XP_025389724.1	XP_025383912.1
	Aspergillus hancockii	KAF7593079.1	KAF7587272.1
	Aspergillus homomorphus	XP_025547265.1	XP_025552456.1
	Aspergillus ibericus	XP_025575496.1	XP_025578007.1
Aspergillus	*Aspergillus luchuensis*	XP_041539789.1	OJZ81278.1
	Aspergillus mulundensis	XP_026604170.1	XP_026604105.1
	Aspergillus neoniger	XP_025476319.1	XP_025484262.1
	Aspergillus niger	EHA26758.1	GKZ64237.1
	Aspergillus piperis	XP_025512668.1	XP_025513870.1
	Aspergillus sclerotiicarbonarius	PYI12272.1	PYI02360.1
	Aspergillus sclerotioniger	XP_025471984.1	XP_025463962.1
	Aspergillus tubingensis	XP_035356720.1	GLB04893.1
	Aspergillus versicolor	XP_040664242.1	UZP48228.1
	Aspergillus welwitschiae	XP_026629939.1	XP_026624638.1
Aureobasidium	*Aureobasidium melanogenum*	XP_040877796.1	KAG9597102.1
Beauveria	*Beauveria bassiana*	XP_008598136.1	XP_008602005.1
	Beauveria brongniartii	OAA37782.1	OAA39919.1
Bipolaris	*Bipolaris maydis*	XP_014081052.1	XP_014078238.1
	Bipolaris oryzae	XP_007689746.1	XP_007682054.1
Biscogniauxia	*Biscogniauxia marginata*	KAI1502855.1	KAI1498195.1
	Biscogniauxia mediterranea	KAI1491791.1	KAI1491404.1
Boeremia	*Boeremia exigua*	KAJ8115054.1	XP_046000075.1
Botryosphaeria	*Botryosphaeria dothidea*	KAF4303495.1	KAF4305872.1
Botryotinia	*Botryotinia calthae*	TEY39080.1	TEY37428.1
	Botryotinia convoluta	TGO61616.1	TGO51629.1
	Botryotinia globosa	KAF7896435.1	KAF7901251.1
	Botryotinia narcissicola	TGO69167.1	TGO56301.1

Genera	Species	Rht1 accession	Rht2 accession
Botrytis	*Botrytis aclada*	KAF7946254.1	KAF7956861.1
	Botrytis byssoidea	XP_038729320.1	XP_038733006.1
	Botrytis cinerea	XP_001557717.1	EMR81961.1
	Botrytis deweyae	XP_038812155.1	XP_038811764.1
	Botrytis fragariae	XP_037188780.1	XP_037193260.1
	Botrytis galanthina	THV55011.1	THV45154.1
	Botrytis paeoniae	TGO28479.1	TGO20471.1
	Botrytis porri	XP_038770654.1	XP_038768623.1
	Botrytis sinoallii	XP_038758083.1	XP_038760845.1
	Botrytis tulipae	TGO11019.1	TGO09160.1
Byssothecium	*Byssothecium circinans*	KAF1950910.1	KAF1948264.1
Cephalotrichum	*Cephalotrichum gorgonifer*	SPN99558.1	SPO03607.1
Cercophora	*Cercophora newfieldiana*	KAK0644497.1	KAK0638609.1
	Cercophora samala	KAK0666350.1	KAK0667676.1
Cladophialophora	*Cladophialophora carrionii*	XP_008726825.1	XP_008725802.1
	Cladophialophora chaetospira	KAJ9603522.1	KAJ9615145.1
Claussenomyces	*Claussenomyces sp.*	KAI9740875.1	KAI9732059.1
Claviceps	*Claviceps africana*	KAG5920846.1	KAG5919883.1
	Claviceps arundinis	KAG5952815.1	KAG5966788.1
	Claviceps capensis	KAG5921672.1	KAG5921632.1
	Claviceps cyperi	KAG5952799.1	KAG5965626.1
	Claviceps digitariae	KAG5972007.1	KAG5982393.1
	Claviceps humidiphila	KAG6112056.1	KAG6118839.1
	Claviceps lovelessii	KAG5991086.1	KAG5986980.1
Claviceps	*Claviceps maximensis*	KAG6000611.1	KAG6002038.1
	Claviceps monticola	KAG5947883.1	KAG5944898.1
	Claviceps purpurea	KAG6139429.1	KAG6132685.1
	Claviceps pusilla	KAG6000469.1	KAG5989301.1
	Claviceps sorghi	KAG5929518.1	KAG5949601.1
	Claviceps spartinae	KAG5989744.1	KAG5994791.1
Clohesyomyces	*Clohesyomyces aquaticus*	ORX91671.1	ORY16921.1
Clonostachys	*Clonostachys byssicola*	CAG9999494.1	CAG9995431.1
	Clonostachys chloroleuca	CAI6100125.1	CAI6093753.1
	Clonostachys rhizophaga	CAH0016028.1	CAH0019948.1
	Clonostachys rosea	CAG9943511.1	CAG9952845.1
	Clonostachys solani	CAH0053102.1	CAH0038538.1
Collariella	*Collariella sp.*	KAJ4302561.1	KAJ4286560.1
Colletotrichum	*Colletotrichum abscissum*	KAI3548229.1	KAI3530008.1
	Colletotrichum acutatum	KAK1728796.1	KAK1724125.1
	Colletotrichum aenigma	XP_037173265.1	XP_037184972.1
	Colletotrichum asianum	KAF0316491.1	KAF0316378.1
	Colletotrichum camelliae	KAH0426174.1	KAH0442418.1
	Colletotrichum caudatum	KAK2056574.1	KAK2059483.1
	Colletotrichum cereale	KAK1983501.1	KAK1986054.1
	Colletotrichum chlorophyti	OLN82217.1	OLN96240.1
	Colletotrichum chrysophilum	KAK1850901.1	XP_053034241.1
	Colletotrichum eremochloae	KAK2012662.1	KAK2005828.1
	Colletotrichum falcatum	KAK1997804.1	KAK1994865.1

Genera	Species	Rht1 accession	Rht2 accession
Colletotrichum	*Colletotrichum filicis*	KAI3546144.1	KAI3528431.1
	Colletotrichum fioriniae	XP_053047741.1	KAJ0331578.1
	Colletotrichum fructicola	KAF4889272.1	XP_031878303.1
	Colletotrichum gloeosporioides	KAH9228900.1	KAH9225977.1
	Colletotrichum godetiae	KAK1688763.1	KAK1657259.1
	Colletotrichum graminicola	XP_008092350.1	XP_008100482.1
	Colletotrichum higginsianum	XP_018156347.1	GJC91227.1
	Colletotrichum incanum	KZL82890.1	OHW97533.1
	Colletotrichum karsti	XP_038751836.1	XP_038743776.1
	Colletotrichum limetticola	KAK0377708.1	KAK0371775.1
	Colletotrichum liriopes	GJC85203.1	GJC77217.1
	Colletotrichum lupini	KAK1717078.1	KAK1705670.1
	Colletotrichum musicola	KAF6844777.1	KAF6838802.1
	Colletotrichum navitas	KAK1589649.1	KAK1573862.1
	Colletotrichum noveboracense	KAJ0291777.1	KAJ0277668.1
	Colletotrichum nupharicola	KAJ0294167.1	KAJ0337848.1
	Colletotrichum nymphaeae	KXH63825.1	KXH45981.1
	Colletotrichum orbiculare	TDZ14397.1	TDZ14673.1
	Colletotrichum orchidophilum	XP_022472465.1	XP_022475743.1
	Colletotrichum paranaense	KAK1543004.1	KAK1540822.1
	Colletotrichum phormii	KAK1654730.1	KAK1635646.1
	Colletotrichum plurivorum	KAF6839083.1	KAF6829629.1
	Colletotrichum salicis	KXH68169.1	KXH39943.1

Genera	Species	Rht1 accession	Rht2 accession
Colletotrichum	*Colletotrichum scovillei*	XP_035338834.1	XP_035327966.1
	Colletotrichum shisoi	TQN74967.1	TQN67810.1
	Colletotrichum siamense	XP_036489658.1	KAF4814982.1
	Colletotrichum sidae	TEA14001.1	TEA21432.1
	Colletotrichum simmondsii	KXH39862.1	KXH40793.1
	Colletotrichum sojae	KAF6819562.1	KAF6806549.1
	Colletotrichum somersetense	KAK2040239.1	KAK2043394.1
	Colletotrichum sublineola	KAK1967522.1	KDN67295.1
	Colletotrichum tamarilloi	KAK1504719.1	KAK1490332.1
	Colletotrichum tanaceti	KAJ0168474.1	KAJ0162061.1
	Colletotrichum tofieldiae	GKT60742.1	KZL66573.1
	Colletotrichum trifolii	TDZ55051.1	TDZ54034.1
	Colletotrichum tropicale	KAJ3960067.1	KAJ3960792.1
	Colletotrichum truncatum	XP_036584982.1	XP_036576291.1
	Colletotrichum viniferum	KAF4928949.1	KAF4925869.1
	Colletotrichum zoysiae	KAK2027693.1	KAK2035655.1
Coniella	*Coniella lustricola*	PSR99022.1	PSR88517.1
Coniochaeta	*Coniochaeta hoffmannii*	KAJ9161119.1	KAJ9155879.1
	Coniochaeta ligniaria	OIW29085.1	OIW23813.1
	Coniochaeta pulveracea	RKU46626.1	RKU48537.1
Conoideocrella	*Conoideocrella luteorostrata*	KAK2612324.1	KAK2616729.1
Cordyceps	*Cordyceps fumosorosea*	XP_018701293.1	XP_018701796.1
	Cordyceps javanica	TQV92045.1	TQV95450.1

Genera	Species	Rht1 accession	Rht2 accession
Cordyceps	*Cordyceps militaris*	ATY63072.1	XP_006674754.1
Cryphonectria	*Cryphonectria parasitica*	XP_040771798.1	XP_040776216.1
Cucurbitaria	*Cucurbitaria berberidis*	XP_040787765.1	XP_040790731.1
Cytospora	*Cytospora leucostoma*	ROW08189.1	ROW16112.1
Dactylonectria	*Dactylonectria estremocensis*	KAH7149514.1	KAH7159663.1
	Dactylonectria macrodidyma	KAH7148757.1	KAH7143671.1
Daldinia	*Daldinia bambusicola*	KAI1806735.1	KAI1805383.1
	Daldinia caldariorum	XP_047790272.1	XP_047785741.1
	Daldinia childiae	XP_033434965.1	XP_033438607.1
	Daldinia decipiens	XP_049104523.1	XP_049095979.1
	Daldinia eschscholtzii	KAI1473488.1	KAI1475320.1
	Daldinia grandis	KAI0108307.1	KAI0095932.1
	Daldinia loculata	KAI2779756.1	KAI2777984.1
	Daldinia vernicosa	XP_047867275.1	XP_047863139.1
Decorospora	*Decorospora gaudefroyi*	KAF1831972.1	KAF1836052.1
Dendryphion	*Dendryphion nanum*	KAH7135752.1	KAH7135333.1
Diaporthe	*Diaporthe ampelina*	KKY39506.1	KKY35200.1
	Diaporthe amygdali	KAK2615727.1	XP_052998701.1
	Diaporthe batatas	XP_044649453.1	XP_044648057.1
	Diaporthe eres	KAI7784925.1	KAI7784403.1
	Diaporthe helianthi	POS75403.1	POS81165.1
	Diaporthe ilicicola	KAI3397926.1	KAI3399409.1
Didymella	*Didymella heteroderae*	KAF3044419.1	KAF3045961.1
	Didymella pomorum	KAJ4403697.1	KAJ4411464.1

Genera	Species	Rht1 accession	Rht2 accession
Durotheca	*Durotheca rogersii*	XP_051368855.1	XP_051373960.1
Echria	*Echria macrotheca*	KAK1750845.1	KAK1751134.1
Emericellopsis	*Emericellopsis atlantica*	XP_046118537.1	XP_046121130.1
Epichloe	*Epichloe festucae*	QPH04205.1	QPH11511.1
Epicoccum	*Epicoccum nigrum*	KAG9204943.1	OSS46826.1
Escovopsis	*Escovopsis weberi*	KOS20329.1	KOS17718.1
Eutypa	*Eutypa lata*	KAI1250716.1	EMR68577.1
Fusarium	*Fusarium acutatum*	KAF4417706.1	KAF4435765.1
	Fusarium albosuccineum	KAF4453631.1	KAF4470643.1
	Fusarium ambrosium	RSM11772.1	RSL97308.1
	Fusarium austroafricanum	KAF4446928.1	KAF4445897.1
	Fusarium austroamericanum	KAF5236077.1	KAF5233753.1
	Fusarium avenaceum	KAH6968718.1	KIL89965.1
	Fusarium beomiforme	KAF4344697.1	KAF4334837.1
	Fusarium chuoi	KAI1013357.1	KAI1019319.1
	Fusarium coffeatum	XP_031013799.1	XP_031011366.1
	Fusarium coicis	KAF5967328.1	KAF5967796.1
	Fusarium culmorum	PTD03166.1	PTD08619.1
	Fusarium decemcellulare	KAF5000569.1	KAF4990227.1
	Fusarium denticulatum	KAF5669558.1	KAF5676768.1
	Fusarium duplospermum	RSL50528.1	RSL66332.1
	Fusarium equiseti	CAG7560528.1	CAG7554771.1
	Fusarium euwallaceae	RTE71192.1	RTE70560.1
	Fusarium falciforme	XP_053008353.1	KAJ4142701.1

Genera	Species	Rht1 accession	Rht2 accession
Fusarium	*Fusarium flagelliforme*	XP_045987014.1	XP_045981550.1
	Fusarium floridanum	RSL77313.1	RSL81449.1
	Fusarium fujikuroi	KLO94116.1	KLO93232.1
	Fusarium gaditjirri	KAF4952308.1	KAF4947967.1
	Fusarium graminearum	XP_011318866.1	PCD36796.1
	Fusarium graminum	KAF4989143.1	KAF4992880.1
	Fusarium heterosporum	KAF5673296.1	KAF5665361.1
	Fusarium irregulare	KAJ4028622.1	KAJ4002951.1
	Fusarium keratoplasticum	XP_052913386.1	XP_052910685.1
	Fusarium kuroshium	RMJ06590.1	RMJ08909.1
	Fusarium langsethiae	GKU13606.1	KPA39552.1
	Fusarium longipes	RGP64061.1	RGP64175.1
	Fusarium mangiferae	XP_041681908.1	XP_041689374.1
	Fusarium mundagurra	KAF5703820.1	KAF5715773.1
	Fusarium musae	XP_044681754.1	XP_044678177.1
	Fusarium napiforme	KAF5543507.1	KAF5530306.1
	Fusarium odoratissimum	XP_031063514.1	XP_031068097.1
	Fusarium oligoseptatum	RSM10484.1	RSM09688.1
	Fusarium oxysporum	RKK72986.1	KAJ4047383.1
	Fusarium piperis	KAJ4307910.1	KAJ4328312.1
	Fusarium poae	XP_044713521.1	OBS22433.1
	Fusarium proliferatum	KAI1009795.1	KAG4256852.1
	Fusarium pseudoanthophilum	KAF5579641.1	KAF5585516.1
Fusarium	*Fusarium pseudocircinatum*	KAF5593813.1	KAF5606259.1
	Fusarium pseudograminearum	QPC77377.1	QPC79958.1
	Fusarium redolens	XP_046051426.1	XP_046045510.1
	Fusarium sarcochroum	KAF4970797.1	KAF4970128.1
	Fusarium solani	XP_046134246.1	XP_046129193.1
	Fusarium solani-melongenae	UPL01582.1	UPL00732.1
	Fusarium sporotrichioides	RGP74396.1	RGP62770.1
	Fusarium tjaetaba	XP_037201359.1	XP_037210905.1
	Fusarium tricinctum	KAH7262271.1	KAH7263618.1
	Fusarium vanettenii	XP_003051169.1	XP_003048134.1
	Fusarium venenatum	XP_025590567.1	KAG8361315.1
	Fusarium verticillioides	XP_018746363.1	XP_018759284.1
	Fusarium xylarioides	KAG5745984.1	KAG5750058.1
	Fusarium zealandicum	KAF4977827.1	KAF4976478.1
Gaeumannomyces	*Gaeumannomyces tritici*	XP_009216301.1	XP_009229452.1
Glonium	*Glonium stellatum*	OCL07134.1	OCL02246.1
Gnomoniopsis	*Gnomoniopsis smithogilvyi*	KAJ4396492.1	KAJ4397375.1
Hapsidospora	*Hapsidospora chrysogena*	KFH41691.1	KFH48365.1
Hirsutella	*Hirsutella minnesotensis*	KJZ78224.1	KJZ71824.1
	Hirsutella rhossiliensis	XP_044715856.1	XP_044720357.1
Hypomontagnella	*Hypomontagnella monticulosa*	KAI0386569.1	KAI0378476.1
	Hypomontagnella submonticulosa	KAI2638882.1	KAI2620953.1
Hypoxylon	*Hypoxylon cercidicola*	KAI1778259.1	KAI1774420.1

Genera	Species	Rht1 accession	Rht2 accession
Hypoxylon	*Hypoxylon crocopeplum*	KAI1380815.1	KAI1377137.1
	Hypoxylon fragiforme	XP_049114466.1	XP_049117066.1
	Hypoxylon fuscum	KAI1401401.1	KAI1399455.1
	Hypoxylon rubiginosum	KAI4863860.1	KAI4864951.1
Ilyonectria	*Ilyonectria destructans*	KAH7011765.1	KAH7002281.1
	Ilyonectria robusta	XP_046104787.1	XP_046110074.1
Immersiella	*Immersiella caudata*	KAK0616255.1	KAK0624188.1
Jackrogersella	*Jackrogersella minutella*	KAI1107063.1	KAI1099809.1
Kalmusia	*Kalmusia sp.*	KAJ4295449.1	KAJ4293461.1
Karstenula	*Karstenula rhodostoma*	KAF2449100.1	KAF2448746.1
Lasallia	*Lasallia pustulata*	KAA6414625.1	KAA6413478.1
Lasiosphaeria	*Lasiosphaeria miniovina*	KAK0733273.1	KAK0706185.1
Lecanicillium	*Lecanicillium saksenae*	KAJ3498506.1	KAJ3497866.1
Leptographium	*Leptographium clavigerum*	XP_014168710.1	XP_014175810.1
Lophiostoma	*Lophiostoma macrostomum*	KAF2657876.1	KAF2648898.1
Lophiotrema	*Lophiotrema nucula*	KAF2116133.1	KAF2113514.1
Lophium	*Lophium mytilinum*	KAF2488524.1	KAF2497128.1
Macrophomina	*Macrophomina phaseolina*	EKG10336.1	EKG11414.1
Macroventuria	*Macroventuria anomochaeta*	XP_033560458.1	XP_033560487.1
Madurella	*Madurella mycetomatis*	KXX75366.1	KXX79238.1
Magnaporthiopsis	*Magnaporthiopsis poae*	KLU92256.1	KLU84765.1
Mariannaea	*Mariannaea sp.*	KAI5462524.1	KAI5458596.1
Massarina	*Massarina eburnea*	KAF2642528.1	KAF2646682.1
Melanomma	*Melanomma pulvis-pyrius*	KAF2800153.1	KAF2786108.1
Metarhizium	*Metarhizium acridum*	XP_007810202.1	KAG8422976.1
	Metarhizium album	XP_040678038.1	XP_040683175.1
	Metarhizium anisopliae	KJK84664.1	KJK80520.1
	Metarhizium brunneum	XP_014545532.1	XP_014548452.1
	Metarhizium guizhouense	KID87172.1	KID92007.1
	Metarhizium humberi	KAH0599959.1	KAH0597174.1
	Metarhizium rileyi	OAA42134.1	OAA43280.1
	Metarhizium robertsii	XP_007819142.2	XP_007822448.1
Microdochium	*Microdochium bolleyi*	KXJ90972.1	KXJ92914.1
	Microdochium nivale	KAJ1326562.1	KAJ1329274.1
	Microdochium trichocladiopsis	XP_046009150.1	XP_046013910.1
Moelleriella	*Moelleriella libera*	OAA33830.1	KZZ90826.1
Monilinia	*Monilinia laxa*	KAB8300938.1	KAB8303359.1
Monosporascus	*Monosporascus cannonballus*	RYO83116.1	RYO94743.1
	Monosporascus ibericus	RYP07719.1	RYP11091.1
Mytilinidion	*Mytilinidion resinicola*	XP_033579010.1	XP_033568734.1
Neofusicoccum	*Neofusicoccum parvum*	EOD43149.1	EOD53031.1
Neonectria	*Neonectria ditissima*	KPM46246.1	KPM40292.1
Niveomyces	*Niveomyces insectorum*	AZHD01000039.1	OAA57048.1
Ophiocordyceps	*Ophiocordyceps sinensis*	KAF4508004.1	EQK98407.1
Ophiostoma	*Ophiostoma piceae*	EPE10043.1	EPE10437.1
Paraphoma	*Paraphoma chrysanthemicola*	KAH7083038.1	KAH7071542.1

Genera	Species	Rht1 accession	Rht2 accession
Penicilliopsis	*Penicilliopsis zonata*	XP_022583757.1	XP_022577075.1
Penicillium	*Penicillium alfredii*	XP_056508930.1	XP_056509782.1
	Penicillium bovifimosum	XP_056523126.1	XP_056526610.1
	Penicillium macrosclerotiorum	XP_056932270.1	XP_056934836.1
	Penicillium odoratum	XP_057001022.1	XP_057000407.1
Periconia	*Periconia macrospinosa*	PVI05090.1	PVH96591.1
Pestalotiopsis	*Pestalotiopsis fici*	XP_007834024.1	XP_007841187.1
Phaeoacremonium	*Phaeoacremonium minimum*	XP_007914921.1	XP_007916963.1
Phaeomoniella	*Phaeomoniella chlamydospora*	KKY25069.1	KKY20074.1
Phialemonium	*Phialemonium atrogriseum*	KAK1762406.1	KAK1762806.1
Pleurostoma	*Pleurostoma richardsiae*	KAJ9156358.1	KAJ9149742.1
Podospora	*Podospora anserina*	XP_001903917.1	XP_001904848.1
Polyplosphaeria	*Polyplosphaeria fusca*	KAF2739724.1	KAF2731913.1
Pseudogymnoascus	*Pseudogymnoascus destructans*	XP_024324285.1	XP_024325152.1
	Pseudogymnoascus verrucosus	XP_018130987.1	XP_018126712.1
Pseudomassariella	*Pseudomassariella vexata*	XP_040716094.1	XP_040718474.1
Purpureocillium	*Purpureocillium lavendulum*	KAJ6440404.1	KAJ6441125.1
Purpureocillium	*Purpureocillium lilacinum*	XP_018175438.1	XP_018181559.1
	Purpureocillium takamizusanense	XP_047844175.1	XP_047841106.1
Pycnora	*Pycnora praestabilis*	KAI9813616.1	KAI9822666.1
Pyrenochaeta	*Pyrenochaeta sp.*	OAL53857.1	OAL46984.1
Pyricularia	*Pyricularia grisea*	KAI6356710.1	KAI6379156.1
	Pyricularia oryzae	KAH8839881.1	KAI6251849.1

Genera	Species	Rht1 accession	Rht2 accession
Rhexocercosporidium	*Rhexocercosporidium sp.*	KAH7350870.1	KAH7346361.1
Rostrohypoxylon	*Rostrohypoxylon terebratum*	KAI1092632.1	KAI1090772.1
Sclerotinia	*Sclerotinia borealis*	ESZ92680.1	ESZ95592.1
	Sclerotinia nivalis	KAJ8059674.1	KAJ8071588.1
	Sclerotinia sclerotiorum	XP_001588350.1	XP_001589362.1
	Sclerotinia trifoliorum	CAD6445273.1	CAD6453695.1
Setomelanomma	*Setomelanomma holmii*	KAF2025903.1	KAF2023082.1
Sodiomyces	*Sodiomyces alkalinus*	XP_028463447.1	XP_028465600.1
Sporothrix	*Sporothrix brasiliensis*	XP_040616120.1	XP_040617365.1
	Sporothrix schenckii	XP_016583713.1	XP_016584143.1
Stachybotrys	*Stachybotrys chartarum*	KFA52060.1	KFA76153.1
	Stachybotrys chlorohalonata	KFA66408.1	KFA67805.1
	Stachybotrys elegans	KAH7326028.1	KAH7322735.1
Stagonospora	*Stagonospora sp. SRC1lsM3a*	OAL06240.1	OAL04400.1
Staphylotrichum	*Staphylotrichum longicolle*	KAG7286337.1	KAG7294653.1
Stromatinia	*Stromatinia cepivora*	KAF7858421.1	KAF7872547.1
Stylonectria	*Stylonectria norvegica*	KAF7554195.1	KAF7554329.1
Talaromyces	*Talaromyces amestolkiae*	XP_040733191.1	XP_040736898.1
	Talaromyces atroroseus	XP_020124160.1	XP_020121501.1
Thermochaetoides	*Thermochaetoides thermophila*	XP_006694985.1	XP_006692471.1
Thermothielavioides	*Thermothielavioides terrestris*	XP_003650890.1	XP_003658264.1
Thozetella	*Thozetella sp.*	KAH8887751.1	KAH8901217.1
Thyridium	*Thyridium curvatum*	XP_030997551.1	XP_030999529.1

Genera	Species	Rht1 accession	Rht2 accession
Tolypocladium	*Tolypocladium ophioglossoides*	KND89648.1	KND93039.1
	Tolypocladium paradoxum	POR38644.1	POR35566.1
Trematosphaeria	*Trematosphaeria pertusa*	XP_033678715.1	XP_033681098.1
Trichoderma	*Trichoderma arundinaceum*	RFU72470.1	RFU78995.1
	Trichoderma asperelloides	KAH8130780.1	KAH8127233.1
	Trichoderma asperellum	XP_024758558.1	UKZ86906.1
	Trichoderma atroviride	XP_013943227.1	UKZ67550.1
	Trichoderma breve	XP_056025069.1	XP_056030790.1
	Trichoderma citrinoviride	XP_024751380.1	XP_024752346.1
	Trichoderma cornu-damae	KAH6609697.1	KAH6605774.1
	Trichoderma gracile	KAH0495172.1	KAH0492740.1
	Trichoderma guizhouense	OPB40233.1	OPB36448.1
	Trichoderma harzianum	XP_024772549.1	KKO97596.1
	Trichoderma longibrachiatum	PTB74489.1	PTB77621.1
	Trichoderma reesei	XP_006961478.1	XP_006968899.1
	Trichoderma semiorbis	KAH0522488.1	KAH0529301.1
	Trichoderma simmonsii	QYT05558.1	QYS95815.1
	Trichoderma virens	XP_013961374.1	XP_013957984.1
Trichothecium	*Trichothecium roseum*	KAI9900351.1	KAI9901541.1
Truncatella	*Truncatella angustata*	XP_045959758.1	KAH8199344.1
Ustilaginoidea	*Ustilaginoidea virens*	XP_043000764.1	XP_042995238.1
Valsa	*Valsa sordida*	ROV96478.1	ROV93292.1
Whalleya	*Whalleya microplaca*	KAI1081251.1	KAI1080707.1
Xylona	*Xylona heveae*	XP_018185144.1	XP_018185306.1
Zopfia	*Zopfia rhizophila*	KAF2193744.1	KAF2191997.1

Accession: A unique identifier assigned to each biological sequence within the NCBI database.

Table S2. Identified motifs in putative Rht1.

Species	Accession number	Motif 1	*p*-value	Motif 2	*p*-value	Motif 3	*p*-value	Motif 4	*p*-value	Motif 5	*p*-value
Fusarium acutatum	KAF4417706.1	TVGATTGFGKLVESV	4.1E-17	LRVQCGPD	1.8E-16	HAGTGTI	2.48E-20	VVPNIQLLNDHQ	4.33E-13	EMARYLSKEGYA	1.12E-11
Fusarium albosuccineum	KAF4453631.1	TVGATVGFKNLIESV	1.6E-17	LHMQCGPD	8.1E-18	HAGTGTI	2.48E-20	VVPNTKLLNDHQ	6.69E-13	EMAKHLSKEGYA	2.19E-14
Fusarium ambrosium	RSM11772.1	TVGATVGFKKLTETA	1.6E-16	LHIQCGPD	1.5E-18	HAGTGTI	2.48E-20	VVPNTQLLNDHQ	8.61E-14	EMAKHLSKEGYA	2.19E-14
Fusarium austroafricanum	KAF4446928.1	TVGATTGFRKLAESV	5.4E-16	LHLQCGPD	2.3E-17	HAGTGTI	2.48E-20	VVPNTQLLNDHQ	8.61E-14	EMAKHLSKEGYA	2.19E-14
Fusarium austroamericanum	KAF5236077.1	TVGATAGFKTLIDSI	8.5E-18	LHVQCGPD	2.2E-15	HAGTGTI	2.48E-20	VVPNTELLDDHQ	1.11E-13	EMAKHLSKEGYA	2.19E-14
Fusarium avenaceum	KAH6968718.1	TVGATAGFKKLVDTV	8.5E-18	LHIQCGPD	3.2E-17	HAGTGTI	2.48E-20	VVPNTQLLNDHQ	8.61E-14	EMAKHLSKEGYA	2.19E-14
Fusarium beomiforme	KAF4344697.1	TVGATTGFRKLIESV	1.1E-18	LRIQCGPD	1.9E-18	HAGTGTI	3.42E-18	VVPNAQLLDDHQ	1.31E-12	EMAKHLSKEGYA	2.19E-14
Fusarium chuoi	KAI1013357.1	TVGATTGFQKLIQSV	4.5E-18	LRVQCGPD	4.4E-17	HAGTGTI	2.48E-20	VVPNIQLLNDHQ	4.33E-13	EMAKHLSKEGYA	2.19E-14
Fusarium coffeatum	XP_031013799.1	TVGATAGFRALIDSV	5.3E-19	LHVQCGPD	2.2E-18	HAGTGTI	2.48E-20	VVPNTQLLDDHQ	4.25E-14	EMAKHLSKEGYA	2.19E-14
Fusarium coicis	KAF5967328.1	TVGATTGFGKLVESV	4.1E-17	LRVQCGPD	1.5E-16	HAGTGTI	2.48E-20	VVPNIQLLNDHQ	4.33E-13	EMAKHLSKEGYA	2.19E-14
Fusarium culmorum	PTD03166.1	TVGATAGFKTLIDSV	2E-18	LHVQCGPD	9.7E-16	HAGTGTI	2.48E-20	VVPNTELLDDHQ	1.11E-13	EMAKHLSKEGYA	2.19E-14
Fusarium decemcellulare	KAF5000569.1	TVGATVGFKNLIESV	1.6E-17	LHIQCGPD	4.8E-20	HAGTGTI	2.48E-20	VVPNTKLLNDHQ	6.69E-13	EMAKHLSKEGYA	2.19E-14
Fusarium denticulatum	KAF5669558.1	TVGATTGFGKLVESV	4.1E-17	LRVQCGPD	6.8E-14	HAGTGTI	2.48E-20	VVPNIQLLNDHQ	4.33E-13	EMAKHLSKEGYA	2.19E-14
Fusarium duplospermum	RSL50528.1	TVGATVGFKKLTETA	1.6E-16	LHIQCGPD	4.5E-19	HAGTGTI	2.48E-20	VVPNTQLLNDHQ	8.61E-14	EMAKHLSKEGYA	2.19E-14
Fusarium equiseti	CAG7560528.1	TVGATAGFKALINSV	1.5E-18	LHVQCGPD	5.7E-18	HAGTGTI	2.48E-20	VVPNTQLLDDHQ	4.25E-14	EMAKHLSKEGYA	2.19E-14
Fusarium euwallaceae	RTE71192.1	TVGATVGFKKLTKTA	2.9E-16	LHIQCGPD	4.5E-19	HAGTGTI	2.48E-20	VVPNTQLLNDHQ	8.61E-14	EMAKHLSKEGYA	2.19E-14
Fusarium falciforme	XP_053008353.1	TVGATVGFRKLTEAA	4.1E-17	LHVQCGPD	8.4E-19	HAGTGTI	2.48E-20	VVPNTQLLNDHQ	8.61E-14	EMAKHLSKEGYA	2.19E-14
Fusarium flagelliforme	XP_045987014.1	TVGATAGFKALIDSV	4.6E-19	LHVQCGPD	1.9E-17	HAGTGTI	2.48E-20	VVPNTQLLDDHQ	4.25E-14	EMAKHLSKEGYA	2.19E-14
Fusarium floridanum	RSL77313.1	TVGATVGFKKLTETA	1.6E-16	LHIQCGPD	4.5E-19	HAGTGTI	2.48E-20	VVPNTQLLNDHQ	8.61E-14	EMAKHLSKEGYA	2.19E-14
Fusarium fujikuroi	KLO94116.1	TVGATTGFEKLIESV	2.6E-18	LRVQCGPD	4.4E-17	HAGTGTI	2.48E-20	VVPNIQLLNDHQ	4.33E-13	EMAKHLSKEGYA	2.19E-14
Fusarium gaditjirri	KAF4952308.1	TVGATTGFGKLVESV	4.1E-17	LRVQCGPD	1.6E-17	HAGTGTI	2.48E-20	VVPNIQLLNDHQ	4.33E-13	EMAKHLSKEGYA	2.19E-14
Fusarium graminearum	XP_011318866.1	TVGATAGFKTLIDSV	2E-18	LHVQCGPD	9.7E-16	HAGTGTI	2.48E-20	VVPNTELLDDHQ	1.11E-13	EMAKHLSKEGYA	2.19E-14
Fusarium graminum	KAF4989143.1	TVGATVGFKKLVGSV	7.2E-17	LHIQCGPD	1.6E-17	HAGTGTI	2.48E-20	VVPNTQLLDDHQ	4.25E-14	EMAKHLSKEGYA	2.19E-14
Fusarium heterosporum	KAF5673296.1	TVGATVGFKKLVGSV	7.2E-17	LHVQCGPD	1.2E-17	HAGTGTI	2.48E-20	VVPNTQLLDDHQ	4.25E-14	EMAKHLSKEGYA	2.19E-14
Fusarium irregulare	KAJ4028622.1	TVGATAGFRALIDSV	5.3E-19	LRVQCGPD	2.7E-17	HAGTGTI	2.48E-20	VVPNTQLLDDHQ	4.25E-14	EMAKHLSKEGYA	2.19E-14

Fusarium keratoplasticum	XP_052913386.1	TVGATVGFKKLTEAT	5.1E-17	LHIQCGPD	1E-18	HAGTGTI	2.48E-20	VVPNTQLLNDHQ	8.61E-14	EMAKHLSKEGYA	2.19E-14
Fusarium kuroshium	RMJ06590.1	TVGATVGFKKLTETA	8.35E-14	LHIQCGPD	1.1E-14	HAGTGTI	2.75E-15	VVPNTQLLNDHQ	8.61E-14	EMAKHLSKEGYA	2.19E-14
Fusarium langsethiae	GKU13606.1	TVGATAGFKTLIDSV	2E-18	LHIQCGPD	1.9E-17	HAGTGTI	2.48E-20	VVPNTQLLNDHQ	8.61E-14	EMAKHLSKEGYA	2.19E-14
Fusarium longipes	RGP64061.1	TVGATAGFKILIDSV	1.8E-17	LHVQCGPD	1.6E-17	HAGTGTI	2.48E-20	VVPNTQLLDDHQ	4.25E-14	EMAKHLSNEGYA	1.56E-12
Fusarium mangiferae	XP_041681908.1	TVGATTGFEKLIESV	2.6E-18	LRVQCGPD	4.4E-17	HAGTGTI	2.48E-20	VVPNIQLLNDHQ	4.33E-13	EMAKHLSKEGYA	2.19E-14
Fusarium mundagurra	KAF5703820.1	TVGATTGFEKLVESA	8E-17	LRVQCGPD	2.3E-17	HAGTGTI	2.48E-20	VVPNIQLLNNHQ	1.25E-13	EMARHLSKEGYA	4.89E-13
Fusarium musae	XP_044681754.1	TVGATTGFGKLVESV	4.1E-17	LRVQCGPD	4.4E-17	HAGTGTI	2.48E-20	VVPNIHLLNDHQ	3.31E-12	EMAKHLSKEGAE	1.07E-8
Fusarium napiforme	KAF5543507.1	TVGATTGFGKLVESV	4.1E-17	LRVQCGPD	4.8E-20	HAGTGTI	2.48E-20	VVPNIQLLNDHQ	4.33E-13	EMAKHLSKEGYA	2.19E-14
Fusarium odoratissimum	XP_031063514.1	TVGATTGFGKLVESV	4.1E-17	LRVQCGPD	2E-16	HAGTGTI	2.48E-20	VVPNIQLLNDHQ	4.33E-13	EMAKHLSKEGYA	2.19E-14
Fusarium oligoseptatum	RSM10484.1	TVGATVGFKKLTETA	1.6E-16	LHIQCGPD	4.5E-19	HAGTGTI	2.48E-20	VVPNTQLLNDHQ	8.61E-14	EMAKHLSKEGYA	2.19E-14
Fusarium oxysporum	RKK72986.1	TVGATTGFGKLVESV	4.1E-17	LRVQCGPD	2E-16	HAGTGTI	2.09E-20	VVPNIQLLNDHQ	4.33E-13	EMAKHLSKEGYA	2.19E-14
Fusarium piperis	KAJ4307910.1	TVGATVGFKKLTEAA	3.6E-17	LHIQCGPD	1E-18	HAGTGTI	2.48E-20	VVPNTQLLNNHQ	1.34E-14	EMAKHLSKEGYA	2.19E-14
Fusarium poae	XP_044713521.1	TVGATAGFKTLIDSV	2E-18	LHVQCGPD	9.6E-17	HAGTGTI	2.48E-20	VVPNTQLLDDHQ	4.25E-14	EMAKHLSKEGYA	2.19E-14
Fusarium proliferatum	KAI1009795.1	TVGATTGFEKLIESV	2.61E-18	LRVQCGPD	4.36E-17	HAGTGTI	2.48E-20	VVPNIQLLNDHQ	4.33E-13	EMAKHLSKEGYA	2.19E-14
Fusarium pseudoanthophilum	KAF5579641.1	TVGATTGFGKLVESV	4.08E-17	LRVQCGPD	4.36E-17	HAGTGTI	2.48E-20	VVPNIQLLNDHQ	4.33E-13	EMAKHLSKEGYA	2.19E-14
Fusarium pseudocircinatum	KAF5593813.1	TVGATTGFGKLVESV	4.08E-17	LRVQCGPD	4.36E-17	HAGTGTI	2.48E-20	VVPNIQLLNDHQ	4.33E-13	EMAKHLSKEGYA	2.19E-14
Fusarium pseudograminearum	QPC77377.1	TVGATAGFKTLIDSV	1.98E-18	LHVQCGPD	9.67E-16	HAGTGTI	2.48E-20	VVPNTELLDDHQ	1.11E-13	EMAKHLSKEGYA	2.19E-14
Fusarium redolens	XP_046051426.1	TVGATTGFTKLVESV	3.41E-18	LRVQCGPD	3.64E-16	HAGTGTI	2.48E-20	VVPNKQLLNDHQ	1.76E-12	EMARHLSKEGYA	4.89E-13
Fusarium sarcochroum	KAF4970797.1	TVGATTGFKKLTESV	2.27E-17	LHVQCGPD	5.66E-18	HAGTGTI	2.48E-20	VVPNTQLLNDHQ	8.61E-14	EMAKHLSKEGYA	2.19E-14
Fusarium solani	XP_046134246.1	TVGATVGFKKLTEAA	3.63E-17	LHVQCGPD	8.4E-19	HAGTGTI	2.48E-20	VVPNTQLLNDHQ	8.61E-14	EMAKHLSKEGYA	2.19E-14
Fusarium solani-melongenae	UPL01582.1	TVGATVGFKKLTEAA	3.63E-17	LHIQCGPD	1.03E-18	HAGTGTI	2.48E-20	VVPNTQLLNDHQ	8.61E-14	EMAKHLSKEGYA	2.19E-14
Fusarium sporotrichioides	RGP74396.1	TVGATAGFKTLIDSV	1.98E-18	LHIQCGPD	5.66E-18	HAGTGTI	2.48E-20	VVPNTQLLNDHQ	8.61E-14	EMAKHLSKEGYA	2.19E-14
Fusarium tjaetaba	XP_037201359.1	TVGATTGFGKLVESV	4.08E-17	LRVQCGPD	4.77E-20	HAGTGTI	2.48E-20	VVPNTQLLNDHQ	8.61E-14	EMAKHLSKEGYA	2.19E-14
Fusarium tricinctum	KAH7262271.1	TVGATAGFKKLVDSV	1.5E-18	LHIQCGPD	2.71E-18	HAGTGTI	2.95E-20	VVPNTQLLNDHQ	8.61E-14	EMAKHLSKEGYA	2.19E-14
Fusarium vanettenii	XP_003051169.1	TVGATVGFRKLTEAA	1.58E-17	LHVQCGPD	8.4E-19	HAGTGTI	2.48E-20	VVPNTQLLNDHQ	8.61E-14	EMAKHLSKEGYA	2.19E-14
Fusarium venenatum	XP_025590567.1	TVGATAGFKTLINSV	5.78E-18	LHVQCGPD	1.15E-17	HAGTGTI	2.48E-20	VVPNTQLLDDHQ	4.25E-14	EMAKHLSKEGYA	2.19E-14
Fusarium verticillioides	XP_018746363.1	TVGATTGFGKLVESV	4.08E-17	LRVQCGPD	4.36E-17	HAGTGTI	2.48E-20	VVPNIQLLNDHQ	4.33E-13	EMAKHLSKEGYA	2.19E-14

Fusarium xylarioides	KAG5745984.1	TVGATTGFEKLIESV	2.61E-18	LRVQCGPD	3.64E-16	HAGTGTI	7.78E-20	VVPNIRLLNDHQ	1.11E-12	EMAKHLSKEGYA	2.19E-14
Fusarium zealandicum	KAF4977827.1	TVGATVGFKKLTEAV	1.12E-18	LHVQCGPD	2.71E-18	HAGTGTI	2.48E-20	VVPNTQLLNDHQ	8.61E-14	EMAKHLSKEGYA	2.19E-14
Dactylonectria estremocensis	KAH7149514.1	TVGATVGFKELTAAV	1.4E-17	LHVQCGPD	6E-17	HAGTGTI	6.66E-20	VVPNTLLLDNHQ	1.93E-13	EMAEHLAKEGYA	8.15E-14
Dactylonectria macrodidyma	KAH7148757.1	TVGATVGFKELTAAV	1.4E-17	LHVQCGPD	6.77E-18	HAGTGTI	6.66E-20	VVPNTLLLDNHQ	1.93E-13	EMAEHLAKEGYA	8.15E-14
Ilyonectria destructans	KAH7011765.1	TVGATVGFKELTAAV	1.4E-17	LHVQCGPD	1.03E-18	HAGTGTI	2.95E-20	VVPNTLLLDNHQ	1.93E-13	EMAKHLSKEGYA	2.19E-14
Ilyonectria robusta	XP_046104787.1	TVGATVGFKELTAAV	1.4E-17	LHVQCGPD	1.03E-18	HAGTGTI	2.95E-20	VVPNTLLLDNHQ	1.93E-13	EMAQHLSKEGYA	4.89E-13
Mariannaea sp. PMI_226	KAI5462524.1	TVGATVGFKALTESV	1.12E-18	LRIQCGPD	1.03E-18	HAGTGTI	6.70E-21	VVPNARLLDNHQ	1.20E-12	EMADHLAKEGYA	3.03E-13
Stylonectria norvegica	KAF7554195.1	TVGATVGFKELTESV	2.27E-18	LHIQCGPD	9.57E-17	HAGTGTI	1.46E-20	VVPNTRLLDDHQ	1.53E-13	ELAQHLAKEGYA	2.59E-12
Neonectria ditissima	KPM46246.1	TVGATVGFKELTAAV	1.06E-15	LHVQCGPD	2.24E-18	HAGTGTI	1.21E-20	VVPNTGLLDDHQ	1.76E-13	EMANHLTREGYA	3.78E-10
Claviceps africana	KAG5920846.1	TVGATVGFEALTRQV	2.55E-17	LHVQCGPD	6.77E-18	HAGTGTI	1.64E-19	VVPNTALLDDHQ	1.25E-13	ELAVHLSKQGYA	1.56E-12
Claviceps arundinis	KAG5952815.1	TVGATVGFSALTRAA	7.19E-17	LHVQCGPD	2.67E-17	HAGTGTI	2.69E-21	VVPNTELLDDHQ	1.11E-13	EMARHLAKEGYA	6.00E-13
Claviceps capensis	KAG5921672.1	TVGATVGFSALTRAV	8.48E-18	LHVQCGPD	1.15E-17	HAGTGTI	6.78E-22	VVPNTELLDDHQ	1.11E-13	EMARHLAKEGYA	6.00E-13
Claviceps cyperi	KAG5952799.1	TVGATVGFPALTRAV	7.47E-18	LHVQCGPD	8.09E-18	HAGTGTI	9.07E-20	VVPNTELLDNHQ	2.36E-14	EMALHLAKEGYA	2.81E-11
Claviceps digitariae	KAG5972007.1	TVGATVGFQALTRQV	6.57E-18	LHVQCGPD	1.62E-17	HAGTGTI	1.90E-19	VVPNTDLLDDHQ	8.61E-14	ELAVHLAKQGYA	1.87E-12
Claviceps humidiphila	KAG6112056.1	TVGATVGFSALTRAA	7.19E-17	LHVQCGPD	7.35E-16	HAGTGTI	2.69E-21	VVPNTELLDDHQ	1.11E-13	EMAHHLAKEGYA	5.57E-12
Claviceps lovelessii	KAG5991086.1	TVGATVGFPALTRQV	1.09E-17	LHVQCGPD	3.27E-18	HAGTGTI	2.69E-21	VVPNTALLDDHQ	1.25E-13	ELATHLAKQGYA	5.57E-12
Claviceps maximensis	KAG6000611.1	TVGATVGFQALTRRV	7.19E-17	LHVQCGPD	1.15E-17	HAGTGTI	1.64E-19	VVPNTGLLDDHQ	1.76E-13	ELAIHLSKQGYA	7.40E-12
Claviceps monticola	KAG5947883.1	TVGATVGFSALTRAV	8.48E-18	LHVQCGPD	1.51E-16	HAGTGTI	6.78E-22	VVPNTELLDDHQ	1.11E-13	EMARHLAKEGYA	6.00E-13
Claviceps purpurea	KAG6139429.1	TVGATVGFSALTRAV	8.48E-18	LHVQCGPD	1.36E-17	HAGTGTI	2.69E-21	VVPNTELLDDHQ	1.11E-13	EMARHLAKEGYA	6.00E-13
Claviceps pusilla	KAG6000469.1	TVGATVGFPALTRQV	1.09E-17	LHVQCGPD	3.27E-18	HAGTGTI	2.69E-21	VVPNTALLDDHQ	1.25E-13	ELATHLAKQGYA	5.57E-12
Claviceps sorghi	KAG5929518.1	TVGATVGFEILTRQV	2.13E-16	LHVQCGPD	6.85E-19	HAGTGTI	1.64E-19	VVPNTALLDDHQ	1.25E-13	ELAVHLSKQGYA	1.56E-12
Claviceps spartinae	KAG5989744.1	TVGATVGFSALTRAA	7.19E-17	LHVQCGPD	2.67E-17	HAGTGTI	2.69E-21	VVPNTELLDDHQ	1.11E-13	EMARHLAKEGYA	6.00E-13
Metarhizium acridum	XP_007810202.1	TVGATVGFEELTKEV	5.78E-18	LHVQCGPD	1.91E-19	HAGTGTI	1.21E-20	VVPNTRLLNDHQ	2.87E-13	EMAKHLARQGYA	9.94E-15
Metarhizium album	XP_040678038.1	TVGATVGFQELTKQV	1.24E-17	LHVQCGPD	1.52E-18	HAGTGTV	2.88E-19	VVPNSRLLDDHQ	1.31E-12	EMAQHLAKQGYA	1.86E-13
Metarhizium anisopliae	KJK84664.1	TVGATVGFEELTKEV	5.78E-18	LHVQCGPD	1.91E-19	HAGTGTI	1.21E-20	VVPNTRLLNDHQ	2.87E-13	EMAEHLARQGYA	2.96E-14
Metarhizium brunneum	XP_014545532.1	TVGATVGFEELTKEV	5.78E-18	LHVQCGPD	1.91E-19	HAGTGTI	1.21E-20	VVPNTRLLNDHQ	2.87E-13	EMAKHLARQGYA	9.94E-15
Metarhizium guizhouense	KID87172.1	TVGATVGFEELTKEV	5.78E-18	LHVQCGPD	1.91E-19	HAGTGTI	1.21E-20	VVPNTRLLNDHQ	2.87E-13	EMAKHLARQGYA	9.94E-15

Metarhizium humberi	KAH0599959.1	**TVGATVGFEELTKEV**	5.78E-18	**LHVQCGPD**	1.91E-19	**HAGTGTI**	1.21E-20	**VVPNTRLLNDHQ**	2.87E-13	**EMAKHLARQGYA**	9.94E-15
Metarhizium rileyi	OAA42134.1	**TVGATVGFEDLTREV**	1.58E-17	**LHVQCGPD**	1.52E-18	**HAGTGTI**	1.21E-20	**VVPNTRLLNDHQ**	2.87E-13	**EMARHLAKQGYA**	1.86E-13
Metarhizium robertsii	XP_007819142.2	**TVGATVGFEELTKEV**	5.78E-18	**LHVQCGPD**	1.91E-19	**HAGTGTI**	1.21E-20	**VVPNTRLLNDHQ**	2.87E-13	**EMAKHLARQGYA**	9.94E-15
Ustilaginoidea virens	XP_043000764.1	**TVGATVGFEQLTKQV**	6.57E-18	**LHVQCGPD**	1.36E-17	**HAGTGTI**	1.00E-20	**VVPNTTLLNDHQ**	5.68E-14	**EMAQYLAKQGYA**	6.43E-12
Conoideocrella luteorostrata	KAK2612324.1	**TVGATVGFAALTKEV**	3.23E-17	**LHIQCGPD**	2.27E-17	**HAGTGTI**	1.42E-19	**VVPNTSLLDDHQ**	5.68E-14	**EMAEHLAKQGYA**	1.44E-14
Moelleriella libera	OAA33830.1	**TVGATTGFEALTREV**	9.62E-18	**LHVQCGPD**	6.77E-18	**HAGTGTI**	4.86E-22	**VVPNTGLLNDHQ**	3.25E-13	**EMARHVAKQGYA**	6.43E-12
Epichloe festucae	QPH04205.1	**TVGATVGFKALTKEV**	1.72E-18	**LHVQCGPD**	3.27E-18	**HAGTGTI**	2.69E-21	**VVPNTGLLDDHQ**	1.76E-13	**EMAKHLAKQGYA**	4.29E-15
Trichoderma arundinaceum	RFU72470.1	**TVGATVGFKKLTEQV**	1.5E-18	**LRIQCGPD**	1.36E-17	**HAGTGTI**	9.07E-20	**VVPNEELLDNHQ**	1.76E-13	**EMAKHLAKEGYA**	2.96E-14
Trichoderma asperelloides	KAH8130780.1	**TVGATVGFKKLTEQV**	8.48E-18	**LRIQCGPD**	2.24E-18	**HAGTGTI**	9.07E-20	**VVPNEELLDNHQ**	1.76E-13	**EMAKHLAKEGYA**	2.96E-14
Trichoderma asperellum	XP_024758558.1	**TVGATVGFKKLTEQV**	8.48E-18	**LRIQCGPD**	2.24E-18	**HAGTGTI**	9.07E-20	**VVPNEELLDNHQ**	1.76E-13	**EMAKHLAKEGYA**	2.96E-14
Trichoderma atroviride	XP_013943227.1	**TVGATVGFKQLTEQV**	1.5E-18	**LRIQCGPD**	4.72E-18	**HAGTGTI**	9.07E-20	**VVPNEELLDNHQ**	1.76E-13	**EMATHLASEGYA**	6.65E-11
Trichoderma breve	XP_056025069.1	**TVGATVGFKQLTEQV**	9.62E-18	**LRIQCGPD**	2.24E-18	**HAGTGTI**	9.07E-20	**VVPNEGLLDNHQ**	2.64E-13	**EMAKHLAKEGYA**	2.96E-14
Trichoderma citrinoviride	XP_024751380.1	**TVGATVGFQQLTEQV**	4.45E-18	**LRIQCGPD**	8.4E-19	**HAGTGTI**	9.27E-19	**VVPNETLLDNHQ**	4.25E-14	**EMAKHLAKEGYA**	2.96E-14
Trichoderma cornu-damae	KAH6609697.1	**TVGATVGFRQLTEQV**	1.98E-18	**LRVQCGPD**	1.92E-17	**NF**		**VVPNEELLDNHQ**	1.76E-13	**EMAQHLAKEGYA**	6.00E-13
Trichoderma gracile	KAH0495172.1	**TVGATVGFKQLTEQV**	1.72E-18	**LRVQCGPD**	6.77E-18	**HAGTGTI**	9.27E-19	**VVPNESLLDNHQ**	8.61E-14	**EMAKHLAKEGYA**	2.96E-14
Trichoderma guizhouense	OPB40233.1	**TVGATVGFKQLTEQV**	9.62E-18	**LRIQCGPD**	2.24E-18	**HAGTGTI**	9.07E-20	**VVPNEGLLDNHQ**	2.64E-13	**EMAKHLAKEGYA**	2.96E-14
Trichoderma harzianum	XP_024772549.1	**TVGATVGFKQLTEQV**	9.62E-18	**LRIQCGPD**	2.24E-18	**HAGTGTI**	9.07E-20	**VVPNEQLLDNHQ**	6.71E-14	**EMAKHLAKEGYA**	2.96E-14
Trichoderma longibrachiatum	PTB74489.1	**TVGATVGFKQLTEQV**	1.72E-18	**LRIQCGPD**	1.25E-18	**HAGTGTI**	9.27E-19	**VVPNETLLDNHQ**	4.25E-14	**EMAKHLAQEGYA**	4.14E-12
Trichoderma reesei	XP_006961478.1	**TVGATVGFKQLTEQV**	1.72E-18	**LRIQCGPD**	8.09E-18	**HAGTGTI**	9.27E-19	**VVPNETLLDNHQ**	4.25E-14	**EMAKHLAKEGYA**	2.96E-14
Trichoderma semiorbis	KAH0522488.1	**TVGATVGFKQLTEQV**	9.62E-18	**LRIQCGPD**	2.24E-18	**HAGTGTI**	9.07E-20	**VVPNEGLLDNHQ**	2.64E-13	**EMAKHLAKEGYA**	2.96E-14
Trichoderma simmonsii	QYT05558.1	**TVGATVGFKQLTEQV**	9.62E-18	**LRIQCGPD**	2.24E-18	**HAGTGTI**	9.07E-20	**VVPNEQLLDNHQ**	6.71E-14	**EMAKHLAKEGYA**	2.96E-14
Trichoderma virens	XP_013961374.1	**TVGATVGFKQLTEQV**	9.62E-18	**LRIQCGPD**	2.24E-18	**HAGTGTI**	6.66E-20	**VVPNEELLDNHQ**	1.76E-13	**EMAKHLAKEGYA**	2.96E-14
Escovopsis weberi	KOS20329.1	**TVGATVGFEALTREA**	9.65E-16	**LRVQCGPD**	4.45E-21	**HAGTGTI**	1.90E-19	**VVPNDKLLDNHQ**	1.89E-12	**EMAKHLSKEGYA**	2.19E-14
Cordyceps fumosorosea	XP_018701293.1	**TVGATVGFRKLTAAV**	9.62E-18	**LRVQCGPD**	5.66E-18	**HAGTGTI**	1.90E-19	**VVPNTELLDDHQ**	1.11E-13	**EMARHLAREGYA**	9.06E-13
Cordyceps javanica	TQV92045.1	**TVGATVGFRKLTAAV**	9.62E-18	**LRVQCGPD**	5.66E-18	**HAGTGTI**	2.18E-19	**VVPNTELLDDHQ**	1.11E-13	**EMAQHLAAEGYA**	5.91E-11
Cordyceps militaris	ATY63072.1	**TVGATVGFRKLTAAV**	2.01E-17	**LRVQCGPD**	5.66E-18	**HAGTGTI**	2.18E-19	**VVANTELLDNHQ**	3.94E-13	**EMAQHLAAEGYA**	5.91E-11
Akanthomyces lecanii	OAA77240.1	**TVGATVGFRKLTAAV**	9.62E-18	**LRVQCGPD**	1.15E-17	**HAGTGTI**	2.18E-19	**VVPNTDLLNDHQ**	1.76E-13	**EMAQHLAKEGYA**	6.00E-13

Akanthomyces muscarius	XP_056049605.1	TVGATVGFRKLTAAV	9.62E-18	LRVQCGPD	1.15E-17	HAGTGTI	2.18E-19	VVPNTDLLNDHQ	1.76E-13	EMAQHLAKEGYA	6.00E-13
Beauveria bassiana	XP_008598136.1	TVGATVGFKKLTAAV	8.48E-18	LHVQCGPD	2.24E-18	HAGTGTI	2.18E-19	VVPNTDLLDDHQ	8.61E-14	EMAKHLAKEGYA	2.96E-14
Beauveria brongniartii	OAA37782.1	TVGATVGFKKLTAAV	8.48E-18	LRVQCGPD	1.85E-18	HAGTGTI	2.18E-19	VVPNTDLLDDHQ	8.61E-14	EMAQHLAKEGYA	6.00E-13
Lecanicillium saksenae	KAJ3498506.1	TVGATVGFRKLTAAV	9.62E-18	LRVQCGPD	3.93E-18	HAGTGTI	2.18E-19	VVPNTDLLNDHQ	1.76E-13	EMAQHLAEEGYA	1.28E-11
Niveomyces insectorum	AZHD01000039.1	TIGATASFVKLLREV	3.59E-13	LLVQTGPD	5.59E-10	HAGSGSI	1.70E-14	VVANPDLQGNHQ	8.40E-12	ELADAVRDAGWA	4.72E-7
Purpureocillium lavendulum	KAJ6440404.1	TVGATVGFEQLTKAA	1.29E-15	LRIQCGPD	1.12E-16	HAGTGTI	1.46E-20	VVPNTGLLNDHQ	3.25E-13	EMAKHLAREGYA	6.14E-14
Purpureocillium lilacinum	XP_018175438.1	TVGATVGFEQLTKAA	4.37E-16	LRIQCGPD	8.2E-17	HAGTGTI	2.09E-20	VVPNTSLLDDHQ	5.68E-14	EMAKHLAREGYA	6.14E-14
Purpureocillium takamizusanense	XP_047844175.1	TVGATVGFEQLTKTA	1.56E-15	LRIQCGPD	2.67E-17	HAGTGTI	3.76E-19	VVPNTGLLDDHQ	1.76E-13	ELAKHLAREGYA	4.89E-13
Hirsutella minnesotensis	KJZ78224.1	TVGATVGFQALTEQA	4.57E-17	LHIQCGPD	5.57E-16	HAGTGTI	6.78E-22	VVPNTSLLHDHQ	7.93E-13	EMAEHLAQEGYA	6.43E-12
Hirsutella rhossiliensis	XP_044715856.1	TVGATVGFRALTQQV	3.9E-18	LHVQCGPD	9.57E-17	HAGTGTI	1.21E-20	VVPNTELLDDHQ	1.11E-13	QMARLLATEGYA	4.29E-8
Tolypocladium ophioglossoides	KND89648.1	TVGATVGFEELTKAV	1.55E-16	LHIQCGPD	6E-17	HATGTI	1.40E-17	VVPNTALLDDHQ	1.25E-13	EMAKHVAKEGYA	2.59E-12
Tolypocladium paradoxum	POR38644.1	AVGATVGFEELTRAV	8.68E-15	LHVQCGPD	1.85E-18	HAGTGTI	1.21E-20	VVPNTALLDDHQ	1.25E-13	EMAKHLAKEGYA	2.96E-14
Ophiocordyceps sinensis	KAF4508004.1	TVGATVGFRTLTQQV	1.4E-17	LHVQCGPD	1.92E-17	HAGTGTI	1.21E-20	VVPNTELLDDHQ	1.11E-13	QMARHLATEGYA	7.57E-10
Clonostachys byssicola	CAG9999494.1	TVGATVGFPKLIKTV	8.76E-16	LRIQCGPD	1.15E-17	HAGTGTI	1.46E-20	VVPNTDLLDDHQ	8.61E-14	EMARYLADEGYA	1.64E-10
Clonostachys chloroleuca	CAI6100125.1	TVGATVGFPKLTKTV	2.26E-15	LRIQCGPD	1.15E-17	HAGTGTI	1.21E-20	VVPNTDLLDDHQ	8.61E-14	EMAHYLADEGYA	6.23E-10
Clonostachys rhizophaga	CAH0016028.1	TVGATVGFPKLIKTV	8.76E-16	LRIQCGPD	1.15E-17	HAGTGTI	1.21E-20	VVPNTDLLDDHQ	8.61E-14	EMARYLADEGYA	1.64E-10
Clonostachys rosea	CAG9943511.1	TVGATVGFPKLIKTV	8.76E-16	LRIQCGPD	1.15E-17	HAGTGTI	1.21E-20	VVPNTDLLDDHQ	8.61E-14	EMARYLADEGYA	1.64E-10
Clonostachys solani	CAH0053102.1	TVGATVGFPKLIKTV	8.76E-16	LRIQCGPD	1.15E-17	HAGTGTI	1.21E-20	VVPNTDLLDDHQ	8.61E-14	EMAHYLADEGYA	6.23E-10
Emericellopsis atlantica	XP_046118537.1	TVGATVGFPDLTRAA	2.13E-16	LRIQCGPD	8.2E-17	HAGTGTI	8.25E-18	VVPNTSLLDDHQ	5.68E-14	ELARHLSKEGYA	2.21E-12
Hapsidospora chrysogena	KFH41691.1	TVGATVGFEDLTRAV	2.55E-17	LRIQCGPD	1.03E-18	HAGTGTI	2.48E-20	VVPNTRLLNDHQ	2.87E-13	ELARHLGNEGYA	6.23E-10
Stachybotrys chartarum	KFA52060.1	TVGATVGFRSLTQTA	3.57E-16	LRIQCGPD	4.77E-20	HAGTGTI	8.18E-19	VVPNESLLDDHQ	3.25E-13	EMAKHLATEGYA	4.14E-12
Stachybotrys chlorohalonata	KFA66408.1	TVGATVGFKSLTQIA	2.26E-15	LRVQCGPD	3.74E-20	HAGTGTI	8.18E-19	VVPNESLLDDHQ	3.25E-13	EMAKHLATEGYA	4.14E-12
Stachybotrys elegans	KAH7326028.1	TVGATVGFQSLIETA	7.2E-16	LRIQCGPD	8.09E-18	HAGTGTI	1.21E-20	VVPNSSLLDDHQ	6.15E-13	EMARHLAKEGYA	6.00E-13
Trichothecium roseum	KAI9900351.1	TVGATVGFEKLTKAV	2.01E-17	LRVQSGPD	8.58E-15	HAGTGTI	1.34E-18	VVPNTSLLDDHQ	5.68E-14	EMAQHMAQAGYA	2.50E-10
Aphanocladium album	KAJ6785788.1	TVGATVGFRKLTAAV	9.62E-18	LRVQCGPD	4.53E-19	HAGTGTI	2.18E-19	VVPNTDLLNDHQ	1.76E-13	EMAQHLAQEGYA	1.91E-11
Colletotrichum abscissum	KAI3548229.1	TVGATARFTQLLTEV	4.56E-19	LTLQCGKD	3.15E-16	HAGTGTI	4.30E-19	VVPNPTLKDNHQ	3.05E-15	ELAEEIQRQGYG	3.56E-12
Colletotrichum acutatum	KAK1728796.1	TVGATARFTQLLTEV	4.56E-19	LTLQCGKD	3.15E-16	HAGTGTI	4.30E-19	VVPNPTLKDNHQ	3.05E-15	ELAEEIQRLGYG	4.63E-10

Colletotrichum aenigma	XP_037173265.1	TVGATARFTQLLTEV	4.56E-19	LTLQCGKD	8.2E-17	NF		VVPNPTLKDNHQ	3.05E-15	ELAEEIQRQGYG	3.56E-12
Colletotrichum asianum	KAF0316491.1	TVGATARFTQLLTEV	4.56E-19	LTLQCGKD	5.48E-14	NF		VVPNPTLKDNHQ	3.05E-15	ELAEEIQRQGYG	3.56E-12
Colletotrichum camelliae	KAH0426174.1	TVGATARFTQLLTEV	4.56E-19	LTLQCGKD	8.2E-17	HAGTGTI	1.42E-19	VVPNPTLKDNHQ	3.05E-15	ELAEEIQRQGYG	3.56E-12
Colletotrichum caudatum	KAK2056574.1	TVGATARFTQLLTEV	4.56E-19	LTLQCGKD	5.58E-19	HAGTGTI	4.30E-19	VVPNPTLKDNHQ	3.05E-15	ELAEEIQRQGYG	3.56E-12
Colletotrichum cereale	KAK1983501.1	TVGATARFTQLLTEV	4.56E-19	LTLQCGKD	5.58E-19	HAGTGTI	4.30E-19	VVPNPTLKDNHQ	3.05E-15	ELAEEIQRQGYG	3.56E-12
Colletotrichum chlorophyti	OLN82217.1	TVGATARFTQLLSEA	7.47E-18	LTLQCGKD	3.71E-17	HAGTGTI	4.30E-19	VVPNPTLKDNHQ	3.05E-15	ELAEEIQRQGYG	3.56E-12
Colletotrichum chrysophilum	KAK1850901.1	TVGATARFTQLLTEV	4.56E-19	LTLQCGKD	8.2E-17	HAGTGTI	1.42E-19	VVPNPTLKDNHQ	3.05E-15	ELAEEIQRQGYG	3.56E-12
Colletotrichum eremochloae	KAK2012662.1	TVGATARFTQLLTEV	4.56E-19	LALQCGKD	2.03E-16	HAGTGTI	4.30E-19	VVPNPTLKDNHQ	3.05E-15	ELAEEIQRQGYG	3.56E-12
Colletotrichum falcatum	KAK1997804.1	TVGATARFTQLLTEV	4.56E-19	LTLQCGKD	8.09E-18	HAGTGTI	2.72E-18	VVPNPTLKDNHQ	3.05E-15	ELAEEIQRQAYG	6.87E-10
Colletotrichum filicis	KAI3546144.1	TVGATARFTQLLTEV	4.56E-19	LTLQCGKD	3.15E-16	HAGTGTI	4.30E-19	VVPNPTLKDNHQ	3.05E-15	ELAEEIQRQGYG	3.56E-12
Colletotrichum fioriniae	XP_053047741.1	TVGATACFTQLLTEV	2.61E-18	LTLQCGKD	3.15E-16	HAGTGTI	4.30E-19	VVPNPTLKDNHQ	3.05E-15	ELAEEIQRQGYG	3.56E-12
Colletotrichum fructicola	KAF4889272.1	TVGATARFTQLLTEV	4.56E-19	LTLQCGKD	8.2E-17	NF		VVPNPTLKDNHQ	3.05E-15	ELAEEIQRQGYG	3.56E-12
Colletotrichum gloeosporioides	KAH9228900.1	TVGATARFTQLLTEV	4.56E-19	LTLQCGKD	8.2E-17	HAGTGTI	1.42E-19	VVPNPTLKDNHQ	3.05E-15	ELAEEIQRQGYG	3.56E-12
Colletotrichum godetiae	KAK1688763.1	TVGATARFTQLLTEV	4.56E-19	LTLQCGKD	3.15E-16	HAGTGTI	4.30E-19	VVPNPTLKDNHQ	3.05E-15	ELAEEIQRQGYG	3.56E-12
Colletotrichum graminicola	XP_008092350.1	TVGATARFTQLLTEV	4.56E-19	LTLQCGKD	5.58E-19	HAGTGTI	4.30E-19	VVPNPTLKDNHQ	3.05E-15	ELAEEIQRQGYG	3.56E-12
Colletotrichum higginsianum	XP_018156347.1	TVGATARFTQLLTEV	4.56E-19	LTLQCGDD	2.73E-16	HAGTGTI	1.06E-19	VVPNPTLKDNHQ	3.05E-15	ELAEEIQRQGYG	3.56E-12
Colletotrichum incanum	KZL82890.1	TVGATARFTQLLTEV	4.56E-19	LTLQCGKD	1.15E-17	HAGTGTI	4.30E-19	VVPNPTLKDNHQ	3.05E-15	ELAEEIQRQGYG	3.56E-12
Colletotrichum karsti	XP_038751836.1	TVGATARFTQLLTEV	4.56E-19	LTLQCGKD	8.44E-16	HAGTGTI	8.25E-18	VVPNPTLKDNHQ	3.05E-15	ELADEIQRQCYG	3.78E-10
Colletotrichum limetticola	KAK0377708.1	TVGATARFTQLLTEV	4.56E-19	LTLQCGKD	3.15E-16	HAGTGTI	4.30E-19	VVPNPTLKDNHQ	3.05E-15	ELAEEIQRQGYG	3.56E-12
Colletotrichum liriopes	GJC85203.1	TVGATARFTQLLTEV	4.56E-19	LTLQCGKD	1.15E-17	HAGTGTI	4.30E-19	VVPNPTLKDNHQ	3.05E-15	ELAEEIQRQGYG	3.56E-12
Colletotrichum lupini	KAK1717078.1	TVGATARFTQLLTEV	4.56E-19	LTLQCGKD	3.15E-16	HAGTGTI	4.30E-19	VVPNPTLKDNHQ	3.05E-15	ELAEEIQRQGYG	3.56E-12
Colletotrichum musicola	KAF6844777.1	TVGATARFTQLLDEV	9.72E-19	LTLQCGRD	2.15E-15	HAGTGTI	1.34E-18	VVPNPTLKDNHQ	3.05E-15	EMAEEIQRQGYG	9.06E-13
Colletotrichum navitas	KAK1589649.1	TVGATARFTQLLTEV	4.56E-19	LTLQCGKD	2.38E-19	HAGTGTI	4.30E-19	VVPNPTLKDNHQ	3.05E-15	ELAEEIQHQGYG	2.03E-10
Colletotrichum noveboracense	KAJ0291777.1	TVGATARFTQLLTEV	4.56E-19	LTLQCGKD	8.2E-17	NF		VVPNPTLKDNHQ	3.05E-15	ELAEEIQRQGYG	3.56E-12
Colletotrichum nupharicola	KAJ0294167.1	TVGATARFTQLLTEV	4.56E-19	LTLQCGKD	8.2E-17	HAGTGTI	1.42E-19	VVPNPTLKDNHQ	3.05E-15	ELAEEIQRQGYG	3.56E-12
Colletotrichum nymphaeae	KXH63825.1	TVGATARFTQLLTEV	4.56E-19	LTLQCGKD	3.15E-16	HAGTGTI	4.30E-19	VVPNPTLKDNHQ	3.05E-15	ELAEEIQRQGYG	3.56E-12
Colletotrichum orbiculare	TDZ14397.1	TVGATARFTQLLTEI	1.98E-18	LVLQCGRD	5.25E-15	HAGTGTI	5.58E-19	VVPNPTLKDNHQ	3.05E-15	ELAEEIQRQGYG	3.56E-12

Colletotrichum orchidophilum	XP_022472465.1	TVGATARFTQLLTEV	4.56E-19	LTLQCGKD	1.3E-16	HAGTGTI	4.30E-19	VVPNPTLKDNHQ	3.05E-15	ELAEEIQRQGYG	3.56E-12
Colletotrichum paranaense	KAK1543004.1	TVGATARFTQLLTEV	4.56E-19	LTLQCGKD	3.15E-16	HAGTGTI	4.30E-19	VVPNPTLKDNHQ	3.05E-15	ELAEEIQRQGYG	3.56E-12
Colletotrichum phormii	KAK1654730.1	TVGATARFTQLLTEV	1.24E-17	LTLQCGKD	3.15E-16	HAGTGTI	4.30E-19	VVPNPTLKDNHQ	3.05E-15	ELAEEIQRQGYG	3.56E-12
Colletotrichum plurivorum	KAF6839083.1	TVGATARFTQLLDEV	9.72E-19	LTLQCGKD	3.6E-15	HAGTGTI	1.34E-18	VVPNPTLKDNHQ	3.05E-15	EMAEEIQRQGYG	9.06E-13
Colletotrichum salicis	KXH68169.1	TVGATARFTQLLTEV	4.56E-19	LTLQCGKD	3.15E-16	HAGTGTI	4.30E-19	VVPNPTLKDNHQ	3.05E-15	ELAEEIQRQGYG	3.56E-12
Colletotrichum scovillei	XP_035338834.1	TVGATARFTQLLTEV	4.56E-19	LTLQCGKD	3.15E-16	HAGTGTI	4.30E-19	VVPNPTLKDNHQ	3.05E-15	ELAEEIQRQGYG	3.56E-12
Colletotrichum shisoi	TQN74967.1	TVGATARFTQLLTEV	4.56E-19	LTLQCGDD	2.36E-16	HAGTGTI	1.06E-19	VVPNPTLKDNHQ	3.05E-15	ELAEEIQRQGYG	3.56E-12
Colletotrichum siamense	XP_036489658.1	TVGATARFTQLLTEV	4.56E-19	LTLQCGKD	8.2E-17	HAGTGTI	1.42E-19	VVPNPTLKDNHQ	3.05E-15	ELAEEIQRQGYG	3.56E-12
Colletotrichum sidae	TEA14001.1	TVGATARFTQLLTEI	1.98E-18	LVLQCGRD	5.25E-15	HAGTGTI	5.58E-19	VVPNPTLKDNHQ	3.05E-15	ELAEEIQRQGYG	3.56E-12
Colletotrichum simmondsii	KXH39862.1	TVGATARFTQLLTEV	4.56E-19	LTLQCGKD	3.15E-16	HAGTGTI	4.30E-19	VVPNPTLKDNHQ	3.05E-15	ELAEEIQRQGYG	3.56E-12
Colletotrichum sojae	KAF6819562.1	TVGATARFTQLLDEV	9.72E-19	LTLQCGKD	1.75E-16	HAGTGTI	1.34E-18	VVPNPTLKDNHQ	3.05E-15	EMAEEIQRQGYG	9.06E-13
Colletotrichum somersetense	KAK2040239.1	TVGATARFTQLLTEV	4.56E-19	LTLQCGKD	5.58E-19	HAGTGTI	4.30E-19	VVPNPTLKDNHQ	3.05E-15	ELAEEIQRQGYG	3.56E-12
Colletotrichum sublineola	KAK1967522.1	TVGATARFTQLLTEV	4.56E-19	LTLQCGKD	3.27E-18	HAGTGTI	4.30E-19	VVPNPTLKDNHQ	3.05E-15	ELAEEIQRQGYG	3.56E-12
Colletotrichum tamarilloi	KAK1504719.1	TVGATARFTQLLTEV	4.56E-19	LTLQCGKD	3.15E-16	HAGTGTI	4.30E-19	VVPNPTLKDNHQ	3.05E-15	ELAEEIQRQGYG	3.56E-12
Colletotrichum tanaceti	KAJ0168474.1	TVGATARFTQLLTEV	4.56E-19	LTLQCGDD	2.36E-16	HAGTGTI	1.06E-19	VVPNPTLKDNHQ	3.05E-15	ELAEEIQRQGYG	3.56E-12
Colletotrichum tofieldiae	GKT60742.1	TVGATARFTQLLTEV	4.56E-19	LTLQCGKD	1.15E-17	HAGTGTI	4.30E-19	VVPNPTLKDNHQ	3.05E-15	ELAEEIQRQGYG	3.56E-12
Colletotrichum trifolii	TDZ55051.1	TVGATARFTQLLTEI	1.98E-18	LVLQCGRD	5.25E-15	HAGTGTI	5.58E-19	VVPNPTLKDNHQ	3.05E-15	ELAEEIQRQGYG	3.56E-12
Colletotrichum tropicale	KAJ3960067.1	TVGATARFTQLLTEV	4.56E-19	LTLQCGKD	8.2E-17	NF		VVPNPTLKDNHQ	3.05E-15	ELAEEIQRQGYG	3.56E-12
Colletotrichum truncatum	XP_036584982.1	TVGATARFTQLLTEV	3.9E-19	LTLQCGKD	3.66E-19	HAGTGTI	2.10E-17	VVPNPTLKDNHQ	3.05E-15	ELADEIQRQGYG	7.40E-12
Colletotrichum viniferum	KAF4928949.1	TVGATARFTQLLTEV	4.56E-19	LTLQCGKD	8.2E-17	NF		VVPNPTLKDNHQ	3.05E-15	ELAEEIQRQGYG	3.56E-12
Colletotrichum zoysiae	KAK2027693.1	TVGATARFTQLLTEV	4.56E-19	LNLQCGKD	2.27E-17	HAGTGTI	4.30E-19	VVPNPTLKDNHQ	3.05E-15	ELAEEIQRQGYG	3.56E-12
Sodiomyces alkalinus	XP_028463447.1	TVGATARFTQLLAEV	1.72E-18	LTVQCGKD	2.24E-18	HAGTGTL	5.62E-17	VVPNPTLKDNHQ	3.05E-15	ELAEEVQSQGYA	1.31E-10
Daldinia bambusicola	KAI1806735.1	TIGASASFKLLIEEV	1.09E-17	LNIQCGPD	1.36E-17	HAGSGSI	1.13E-17	AVPNPTLMDNHQ	5.68E-14	EIADEMARQGHL	1.01E-9
Daldinia caldariorum	XP_047790272.1	TIGASASFKLLIEEV	1.09E-17	LNVQCGPD	6.77E-18	HAGSGSI	1.13E-17	AVPNPTLMDNHQ	5.68E-14	EIADEMARQGHL	1.01E-9
Daldinia childiae	XP_033434965.1	TIGASASFKLLIEEV	1.09E-17	LTIQCGPD	2.27E-17	HAGSGSI	1.13E-17	AVPNPTLMDNHQ	5.68E-14	EIAEEMDSQGHL	9.07E-9
Daldinia decipiens	XP_049104523.1	TIGASASFKLLIEEV	1.09E-17	LIIQCGPD	9.65E-18	HAGSGSI	1.13E-17	AVPNPTLMGNHQ	5.64E-13	EIAEEMDSQGHL	9.07E-9
Daldinia eschscholtzii	KAI1473488.1	TIGASASFKLLIEEV	5.13E-17	LTIQCGPD	8.4E-19	HAGSGSI	1.13E-17	AVPNPTLMDNHQ	5.68E-14	EIAEEMEKQGHL	4.18E-10

Daldinia grandis	KAI0108307.1	TIGASASFQPLIEEV	1.58E-17	LTLQCGPD	3.93E-18	HAGSGSI	1.13E-17	AVANPTLMDNHQ	6.69E-13	EIAEEMDSQGHL	9.07E-9
Daldinia loculata	KAI2779756.1	TIGASASFKLLIEEV	1.09E-17	LIIQCGPD	9.65E-18	HAGSGSI	1.13E-17	AVPNPTLMDNHQ	5.68E-14	EIAEEMDSQGHL	9.07E-9
Daldinia vernicosa	XP_047867275.1	TIGASASFKLLIEEV	1.58E-17	LIIQCGPD	9.65E-18	HAGSGSI	1.13E-17	AVPNPTLMDNHQ	5.68E-14	EIAEEMHSQGHL	1.47E-18
Annulohypoxylon bovei	KAI2473205.1	TVGASASFKPLIEEM	6.12E-15	LIIQCGPD	1.11E-15	HAGSGSI	3.05E-18	AVPNPALMGNHQ	1.64E-12	EIADDMEEQGFL	2.17E-18
Annulohypoxylon moriforme	KAI1454471.1	TVGASASFKPLIEEV	2.36E-16	LIVQCGPD	1.89E-15	HAGSGSI	2.16E-18	AVPNLALMGNHQ	2.81E-11	EIADEMEKQGFL	3.41E-10
Annulohypoxylon nitens	KAI0892314.1	TVGASASFKPLIEEV	2.36E-16	LIVQCGPD	1.65E-15	HAGSGSI	1.55E-17	AVPNPALMGNHQ	1.64E-12	EIAEEMEKQGFL	2.03E-10
Annulohypoxylon stygium	KAI1446163.1	TVGASASFKPLIEEV	2.36E-16	LIVQCGPD	1.65E-15	HAGSGSI	2.16E-18	AVPNPALMGNHQ	1.64E-12	EIAEEMEKQGFL	2.03E-10
Annulohypoxylon truncatum	XP_047856260.1	TVGASASFKPLIEEV	2.36E-16	LIVQCGPD	9.67E-16	HAGSGSI	3.05E-18	AVPNPALMGNHQ	1.64E-12	EIADEMEKQGFL	3.41E-10
Hypoxylon cercidicola	KAI1778259.1	TVGASASFKPLIEEV	5.07E-18	LVVQCGPD	1.65E-15	HAGSGSI	3.14E-17	AVPNTSLMDNHQ	1.11E-13	EIAVEMERQGFL	7.57E-10
Hypoxylon crocopeplum	KAI1380815.1	TVGASASFKSLIKEI	1E-16	LIVQCGPD	2.03E-16	HAGSGSI	1.02E-17	AVPNPALMGNHQ	1.64E-12	EIAEEMEKQGFL	2.03E-10
Hypoxylon fragiforme	XP_049114466.1	TIGASASFQPLIAEV	7.94E-16	LIVQYGPD	6E-17	HAGSGSI	3.82E-17	AVPNPSLMDNHQ	1.11E-13	EIATEMENEGFL	8.36E-9
Hypoxylon fuscum	KAI1401401.1	TVGASASFKSLVDEV	5.74E-17	LMVQCGPD	6.72E-15	HAGSGSI	2.57E-17	AVPNPTLMDNHQ	5.68E-14	EIADEMEKQGLL	2.76E-9
Hypoxylon rubiginosum	KAI4863860.1	TVGASASFKPLIEEV	5.07E-18	LVVQCGPD	8.44E-16	HAGSGSI	1.72E-17	AVPNTSLMDNHQ	1.11E-13	EIAEEMEEQGFL	1.34E-9
Hypomontagnella monticulosa	KAI0386569.1	TVGASASFKSLIDEV	3.23E-17	LIVQCGPD	1.97E-14	HAGSGSI	3.46E-17	AVPNPTLMGNHQ	5.64E-13	EIAKDMDKKEFL	6.88E-7
Hypomontagnella submonticulosa	KAI2638882.1	TVGASASFKSLIDEV	1.55E-16	LIVQCGPD	5.94E-15	HAGSGSI	3.46E-17	AVPNPTLMGNHQ	5.64E-13	EIAKEMDKKGFC	2.73E-8
Jackrogersella minutella	KAI1107063.1	TVGASASFKPLIEEV	1.09E-17	LIVQCGPD	3.93E-18	HAGSGSI	5.35E-18	AVPNPALMGNHQ	1.64E-12	EIADEMEKQGFL	3.41E-10
Rostrohypoxylon terebratum	KAI1092632.1	TVGASSSFKPLIEEV	2.06E-15	LIIQCGPD	9.67E-16	HAGAGSI	4.21E-17	AVPNPALMDNHQ	2.40E-13	EIAEEMGKQGFL	3.60E-9
Biscogniauxia marginata	KAI1502855.1	TIGATAGFRPLVEEV	3.9E-18	LIVQCGPD	1.12E-16	HAGSGSI	1.51E-18	AVPNPSLMDNHQ	1.11E-13	ELAEEMELQGYA	2.03E-10
Biscogniauxia mediterranea	KAI1491791.1	TIGATAGFRPLIEEV	1.5E-18	LVVQCGPD	2.15E-15	HAGSGSI	2.45E-16	AVANPALMDNHQ	1.89E-12	ELAEEMERQGYA	6.00E-13
Durotheca rogersii	XP_051368855.1	TVGASSSFRRLLQEV	1.12E-14	VIVQCGPD	5.25E-15	HGGSGSI	1.31E-16	AVPNPELMDNHQ	2.15E-13	EIVEGMAQEGFL	1.27E-6
Whalleya microplaca	KAI1081251.1	TVGATASFRPLIEEV	1.5E-18	LIVQCGPD	2.79E-15	HAGSGSL	1.73E-15	AVPNTSLMGNHQ	9.35E-13	EIAIELEKDNYV	1.16E-8
Microdochium bolleyi	KXJ90972.1	TIGATAGFRPLLEEV	3.88E-20	LLVQCGPD	8.2E-17	HAGSGSI	2.51E-19	AVPNESLMDNHQ	5.64E-13	ELADEMQTQGFL	1.11E-9
Microdochium nivale	KAJ1326562.1	TIGATAGFRPLLDEV	3.9E-19	LLVQCGPD	7.6E-15	HAGSGSI	2.51E-19	AVPNESLMDNHQ	5.64E-13	ELANEMQSQGFL	5.51E-9
Microdochium trichocladiopsis	XP_046009150.1	TIGATAGFRPLLDEV	3.9E-19	LVVQCGPD	8.58E-15	HAGSGSI	2.51E-19	AVPNESLMDNHQ	5.64E-13	ELADEMQTQGFL	1.11E-9
Monosporascus cannonballus	RYO83116.1	TVGATAGFQPLLAEV	3.23E-17	LVVQCGPD	1.3E-13	HAGSGSI	7.21E-19	AVPNPALMDNHQ	2.40E-13	ELAEEMERQGYL	3.56E-12
Monosporascus ibericus	RYP07719.1	TVGATAGFRPLLAEV	1.58E-17	LVIQCGPD	7.6E-15	HAGSGSI	4.90E-19	AVPNPALMDNHQ	2.40E-13	ELAEEMERQGYA	6.00E-13
Pestalotiopsis fici	XP_007834024.1	TIGSIASFKSLITEV	1.71E-15	LVVQCGPD	1.09E-14	HAGAGTI	6.11E-15	VVPNTSLMDNHQ	5.61E-15	ELAEELERQGYL	4.14E-12

Truncatella angustata	XP_045959758.1	TIGSIASFKDLVEEV	6.12E-15	LIVQCGPD	1.09E-14	HAGAGTI	2.35E-14	VVPNTSLMDNHQ	2.25E-18	**NF**	
Pseudomassariella vexata	XP_040716094.1	TIGATASFKDLITEV	2.48E-15	LIVQCGPD	1.31E-11	HAGAGTI	2.45E-16	VVPNATLMDNHQ	2.87E-13	QLAKAMDKEGYL	2.53E-9
Eutypa lata	KAI1250716.1	TVGATAGFQPLLQEV	6.57E-18	LVIQCGPD	8.58E-15	HAGSGTI	2.88E-19	AVANPALMDNHQ	1.89E-12	ELAKEMERQGFL	7.47E-11
Cercophora newfieldiana	KAK0644497.1	TVGATAGFRQLLAEV	2.27E-17	LSVQCGPD	3.6E-15	HAGTGTV	2.72E-18	VVPNPTLMDNHQ	4.60E-16	ELAEEVENQRWG	2.01E-8
Cercophora samala	KAK0666350.1	TIGSIASFLPLLEQV	2.48E-15	LTVQCGPN	8.87E-13	HAGAGTI	1.15E-15	VVPNPTLMDNHQ	4.60E-16	ELAVEIQRQGWA	8.38E-11
Lasiosphaeria miniovina	KAK0733273.1	TVGATAGFPQLLAEV	4.08E-17	LEVQCGPD	3.92E-14	HAGSGTI	1.59E-15	AVPNPTLMDNHQ	5.68E-14	ELADVIEPQGWG	2.73E-8
Immersiella caudata	KAK0616255.1	TVGATAGFRPLLAEV	4.84E-16	LVVQCGPD	2.73E-12	HAGTGTV	2.72E-18	VVPNPTLMNNHQ	5.61E-15	ELAEECEKQRWA	2.31E-9
Apiosordaria backusii	KAK0701482.1	TIGSIASFLPLLEQ	2.48E-15	LTVQCGPN	1.72E-12	HAGAGTI	1.15E-15	VVPNPTLMDNHQ	4.60E-16	ELAVEVQRQGWA	4.63E-10
Collariella sp. IMI 366227	KAJ4302561.1	TIGATAGFRSLLEE	3.16E-20	LHVQCGPD	1.23E-14	**NF**		VVANPTLMDNHQ	9.30E-14	ELAESLEEQGVA	3.98E-8
Thermothielavioides terrestris	XP_003650890.1	TVGATASFRALLDE	8.38E-20	LEAQCGPD	4.47E-13	**NF**		VVANPTLMDNHQ	9.30E-14	ELAETLEEENRA	2.63E-7
Staphylotrichum longicolle	KAG7286337.1	TVGATAGFRSLLEE	2.07E-20	LLDVQCGPD	2.49E-14	**NF**		VVANPTLMDNHQ	9.30E-14	ELAESLEEQNLA	6.18E-8
Thermochaetoides thermophila	XP_006694985.1	TVGATAGFRDLLDE	1.3E-18	LDVQCGPD	2.22E-14	**NF**		VVANPTLMDNHQ	9.30E-14	ELAEDLARRKLA	1.19E-6
Podospora anserina	XP_001903917.1	TIGSIASFLPLLEQV	2.48E-15	LTVQCGPN	8.87E-13	HAGAGTI	1.47E-15	VVPNPTLMDNHQ	4.60E-16	ELAVEVQRQGWA	4.63E-10
Echria macrotheca	KAK1750845.1	TVGATAGFRPLLERV	2.27E-17	LEVQCGPD	8.6E-12	HAGTGTI	8.21E-17	VVPNPTLMDNHQ	4.60E-16	ELADEIARNGWG	1.22E-9
Madurella mycetomatis	KXX75366.1	TVGATASFRVLIEEV	3.32E-19	LDVQCGPD	6E-17	HGGSGTV	4.64E-17	VVVNPTLMDNHQ	1.02E-12	ELAQSLADQNWA	2.01E-8
Diaporthe ampelina	KKY39506.1	TGGATVPFVALLEEA	6.34E-14	VYLQCGIA	4.47E-13	HAGTGTI	3.83E-15	IVANTTLMDDHQ	1.89E-12	**NF**	
Diaporthe amygdali	KAK2615727.1	TGGATVPFVPLLEEA	8.7E-14	VYLQCGTA	3.5E-14	HAGTGTI	3.20E-16	IVANTTLMDDHQ	1.89E-12	**NF**	
Diaporthe batatas	XP_044649453.1	TGGATVPFVGLLEET	1.61E-13	VFLQCGSV	1.18E-12	HAGTGTI	4.53E-16	IVANSTLMDDHQ	8.92E-12	**NF**	
Diaporthe eres	KAI7784925.1	TGGATVPFVALLEEA	6.34E-14	VYLQCGAA	1.45E-13	HAGTGTI	5.24E-15	IVANTTLMDDHQ	1.89E-12	**NF**	
Diaporthe helianthi	POS75403.1	TGGATVPFVGLLEEA	1.28E-13	VYLQCGSV	2.2E-13	HAGTGTI	4.84E-15	IVANSTLMDDHQ	8.92E-12	**NF**	
Diaporthe ilicicola	KAI3397926.1	TGGATVPFVALLEEA	6.34E-14	VYLQCGAA	2.7E-13	HAGTGTI	3.83E-15	IVANTTLMDDHQ	1.89E-12	**NF**	
Valsa sordida	ROV96478.1	**NF**		LHLQCNTF	9.76E-13	**NF**		IVANPNLMDNHQ	2.70E-12	**NF**	
Cytospora leucostoma	ROW08189.1	TGGATVPFKELLHEV	4.99E-14	IDIQCGSE	1.79E-13	HAGTGTI	1.24E-15	IVANPNLMDNHQ	2.70E-12	**NF**	
Coniella lustricola	PSR99022.1	TGGATVVFRELVDET	1.39E-13	LLLQCGVY	8.05E-13	HAGTGTI	8.95E-15	IVANPNLMDNHQ	2.70E-12	**NF**	
Gnomoniopsis smithogilvyi	KAJ4396492.1	TGGATVVFKELLDEV	4.6E-13	VLIQCGSY	1.55E-11	**NF**		IVANPNLMDNHQ	2.70E-12	**NF**	
Cryphonectria parasitica	XP_040771798.1	TGGATVVFKELIDET	5.86E-14	LLLQCGTY	1.89E-12	HAGTGTI	2.72E-14	IVANPNLMDNHQ	2.70E-12	**NF**	
Pyricularia grisea	KAI6356710.1	TIGATAGFRALLEQV	5.78E-18	MTVQCGSD	1.02E-11	HAGAGTV	4.19E-14	VVPNEGLMDNHQ	2.40E-13	ELATHLDQERWA	3.43E-8

Pyricularia oryzae	KAH8839881.1	TIGATAGFRSLLEQV	6.57E-18	MTVQCGSD	1.02E-11	NF		VVPNEGLMDNHQ	2.40E-13	ELATHLDKERWA	5.99E-9
Magnaporthiopsis poae	KLU92256.1	TVGATASFRPLLAEI	5.74E-17	IQVQCGPD	6.63E-12	HAGAGTV	1.75E-14	VVPNEDLLDNHQ	1.37E-13	NF	
Gaeumannomyces tritici	XP_009216301.1	TVGATASFRPLLAEI	5.74E-17	IQVQCGPD	2.55E-11	HAGAGTV	2.35E-14	IVPNEDLLDNHQ	9.35E-13	NF	
Sporothrix brasiliensis	XP_040616120.1	TVGATASFIRLLREV	4.45E-18	LVVQTGPD	6.08E-12	HAGSGSI	7.55E-16	VVANPDLLGNHQ	2.03E-12	ELADAVHDAGWA	1.89E-7
Sporothrix schenckii	XP_016583713.1	TVGATASFIKLLREV	4.56E-19	LVVQTGPD	6.08E-12	HAGSGSI	7.55E-16	VVANPDLLGNHQ	2.03E-12	ELADAVHDAGWA	1.89E-7
Grosmannia clavigerum	XP_014168710.1	TVGATASFYRLLEEI	2.01E-17	LLVQTGPD	2.49E-12	HAGSGSI	6.80E-17	VVANGALLGNHQ	3.61E-11	ELAEAVQTAGWA	3.43E-8
Ophiostoma piceae	EPE10043.1	TVGATASFQKLLSDI	2.55E-17	LVVQTGPD	1.3E-12	HGGSGSI	7.12E-15	VVANPDLLGNHQ	2.03E-12	ELADAVDEAGWA	1.09E-7
Coniochaeta hoffmannii	KAJ9161119.1	TTGSMASFKALLQEV	1.06E-15	LEVQCGPD	3.64E-16	HAGSGTI	9.90E-17	VVANPDLLGNHQ	2.03E-12	NF	
Coniochaeta ligniaria	OIW29085.1	TTGSIASFRPLLEEV	1.25E-16	LEVQCGPD	7.6E-15	HAGSGTI	2.10E-17	IVPNPTLLDNHQ	6.71E-14	EIASEMAAQDLA	1.43E-6
Coniochaeta pulveracea	RKU46626.1	TAGSVVSYRALLASV	4.34E-12	LEVQCGDD	8.05E-13	HAGAGTI	8.93E-16	VVANPDLMNNHQ	5.09E-13	ELADECAAKGYA	1.07E-8
Cephalotrichum gorgonifer	SPN99558.1	SVGATAPFTPLVSAA	9.65E-16	LVVQAGSD	4.39E-14	HAGTGTV	1.21E-14	VIPNPALQDNHQ	6.51E-12	ELAEELQNQGYA	6.43E-12
Thozetella sp. PMI_491	KAH8887751.1	TVGATAGFRPLLEEI	1.24E-17	LIVQCGPD	1.45E-13	HAGSGTI	1.09E-16	VVPNPTLMNNHQ	5.61E-15	ELAEEVERQNWA	8.34E-10
Thyridium curvatum	XP_030997551.1	NF		MAVQCGPD	6.63E-12	HCGSGTI	1.47E-15	AVPNPTLADNHQ	1.02E-12	ELAEVVDKNRWA	1.65E-7
Phialemonium atrogriseum	KAK1762406.1	TVGSIASFRALLTEI	1.12E-16	LEVQCGPD	1.31E-11	HAGAGTV	3.20E-16	VVPNSTLLDNHQ	9.30E-14	ELAEECEKQNWA	6.23E-10
Phaeoacremonium minimum	XP_007914921.1	TGGATVAFRELSDEV	1.61E-13	LDVQCGKY	5.57E-16	NF		VVANPNLMDNHQ	5.64E-13	NF	
Pleurostoma richardsiae	KAJ9156358.1	TVGATATFPALVDEV	4.69E-15	VEIQCGDD	1.09E-9	NF		IVPNPDLMDNHQ	1.93E-13	NF	
Ascochyta clinopodiicola	KAJ4351106.1	TTGATAPFTALIESV	8.19E-21	LLVQYGSA	1.09E-14	HAGSGSI	2.95E-20	VVPNTSLLDNHQ	8.97E-15	ELAVAMERSGYL	5.65E-10
Ascochyta lentis	KAF9700101.1	TTGATAPFTALIESV	8.19E-21	VLIQYGSA	3.92E-14	HAGSGSI	8.23E-21	VVPNTSLLDNHQ	8.97E-15	ELAVAMERSGYL	5.65E-10
Ascochyta rabiei	XP_059492683.1	TTGATAPFAALIESV	7.22E-19	LLVQHGSA	2.22E-14	HAGSGSI	1.92E-18	VVPNTSLLDNHQ	8.97E-15	ELAVAMERSGYL	5.65E-10
Didymella heteroderae	KAF3044419.1	TTGATAPFTALIESV	8.19E-21	LLVQYGSA	1.15E-17	HAGSGSI	8.23E-21	VVPNTSLLDNHQ	8.97E-15	ELADAMERSGYL	3.07E-10
Didymella pomorum	KAJ4403697.1	TTGATAPFTALIESV	8.19E-21	LLVQYGSA	1.11E-15	HAGSGSI	8.23E-21	VVPNTGLLDNHQ	4.25E-14	ELADAMERSGYL	3.07E-10
Boeremia exigua	KAJ8115054.1	TTGATAPFTALIESV	8.19E-21	LLVQYGSA	4.63E-15	HAGSGSI	8.25E-18	VVPNTSLLDNHQ	8.97E-15	ELAVAMERSGYL	5.65E-10
Macroventuria anomochaeta	XP_033560458.1	TTGATAPFTALIESV	8.19E-21	LLVQHGSA	1.61E-13	HAGSGSI	8.23E-21	VVPNTSLLDNHQ	8.97E-15	ELAVAMERSGYL	5.65E-10
Epicoccum nigrum	KAG9204943.1	TTGATAPFNALIESV	8.19E-21	LLVQYGSA	1.89E-15	HAGSGSI	7.21E-19	VVPNTGLLDNHQ	4.25E-14	ELADAMERSGYL	3.07E-10
Stagonospora sp. SRC1lsM3a	OAL06240.1	TTGATAPFTALIESA	2.82E-19	LLIQYGTA	2.79E-14	HAGSGTI	1.05E-18	VVPNTALLDNHQ	2.63E-14	ELAVAMERSNYL	2.31E-9
Byssothecium circinans	KAF1950910.1	TTGATAPFTALIEAV	6.36E-21	LLVQHGTA	3.91E-12	HAGSGSI	2.51E-19	VVPNTQLLDNHQ	4.92E-15	ELAAAMEKSNYL	8.36E-9
Massarina eburnea	KAF2642528.1	TTGATAPFTALIESV	6.36E-21	LLVQYGTA	6.12E-14	HAGSGSI	5.69E-20	VVPNTQLLDNHQ	4.92E-15	ELAVAMEKSNYL	1.9E-9

Bipolaris maydis	XP_014081052.1	TTGATAPFTALIESI	8.38E-20	LLVQYGSA	1.27E-15	HAGSGSI	8.23E-21	VVPNTGLLDNHQ	4.25E-14	ELAVAMERNNYL	2.76E-9
Bipolaris oryzae	XP_007689746.1	TTGATAPFTALIESV	8.19E-21	LLVQYGSA	9.67E-16	HAGSGSI	8.23E-21	VVPNTGLLDNHQ	4.25E-14	ELAVAMERNNYL	2.76E-9
Decorospora gaudefroyi	KAF1831972.1	TTGATAPFTALIESV	8.19E-21	LLVQYGSA	3.64E-16	HAGSGSI	8.23E-21	VVPNTGLLGNHQ	4.74E-13	ELAVAMERQNYV	3.41E-10
Kalmusia sp. IMI 367209	KAJ4295449.1	TTGATAPFTGLIESV	5.75E-20	VFVQFGSA	1.83E-11	HAGSGTI	9.07E-20	VVPNRALLDNHQ	1.40E-12	ELAVAMEEANYL	2.17E-8
Karstenula rhodostoma	KAF2449100.1	TTGATAPFTKLIEAV	4.9E-21	ILIQYGTA	2.49E-12	HAGSGSV	8.18E-19	VVPNTALLDNHQ	2.63E-14	ELAVAMDRAGYV	3.60E-9
Paraphoma chrysanthemicola	KAH7083038.1	TTGATAPFTALIESV	8.19E-21	LLIQYGSA	3.15E-16	HAGSGTI	2.95E-20	VVPNTSLLDNHQ	8.97E-15	ELAVAMERSNYL	2.31E-9
Setomelanomma holmii	KAF2025903.1	TTGATAPFTALIESV	8.19E-21	LLVQYGSA	8.58E-15	HAGSGSI	2.95E-20	VVPNTALLDNHQ	2.63E-14	ELAVAMERSNYL	2.31E-9
Pyrenochaeta sp. DS3sAY3a	OAL53857.1	TTGATAPFTALIESV	8.19E-21	LLVQYGSA	7.6E-15	HAGSGTI	2.95E-20	VVPNTGLLDNHQ	4.25E-14	ELAVAMERSNYL	2.31E-9
Cucurbitaria berberidis	XP_040787765.1	TTGATAPFTALIESV	8.19E-21	LLVQYGSA	2.15E-15	HAGSGSV	2.18E-19	VVPNIGLLDNHQ	2.40E-13	ELAVAMERNQYL	2.53E-8
Clohesyomyces aquaticus	ORX91671.1	TTGATAPFEALIESV	3.88E-20	LLVQYGTA	8.49E-14	HAGSGSI	1.42E-19	VVPNTSLLGNHQ	1.76E-13	ELATAMAKNNYL	5.06E-9
Dendryphion nanum	KAH7135752.1	TTGATAPFTGLIESV	4.73E-20	LLVQYGTA	7.62E-14	HAGSGSI	1.51E-18	VVPNTLLLDNHQ	1.93E-13	ELAVAMERSGYL	5.65E-10
Lophiostoma macrostomum	KAF2657876.1	TTGATAPFTGLIESI	1.09E-17	LLIQYGTA	1.61E-13	HAGSGSI	2.51E-19	VVPNTALLDNHQ	2.63E-14	ELAVAMERSNYL	2.31E-9
Lophiotrema nucula	KAF2116133.1	TTGATAPFIALIEAI	2.99E-18	LLIQYGTA	2.79E-15	HAGSGSI	2.51E-19	VVPNTALLDNHQ	2.63E-14	ELAIAMEKSGYL	1.22E-9
Melanomma pulvis-pyrius	KAF2800153.1	TTGATAPFTGLIESV	8.38E-19	LLIQYGSA	1.27E-15	HAGSGSI	5.11E-17	VVPNTELLDNHQ	2.36E-14	NF	
Polyplosphaeria fusca	KAF2739724.1	TTGATAPFTELIRSV	2.07E-20	LLIQYGTA	9.46E-14	HAGSGTI	2.95E-20	VVPNTSLLDNHQ	8.97E-15	ELAAAMERSNYL	9.84E-9
Trematosphaeria pertusa	XP_033678715.1	TTGATAPFTALIESL	5.78E-18	LLIQFGTA	1.97E-14	HAGSGSI	4.28E-18	VVPNDQLLDNHQ	3.55E-13	ELAIAMERSNYL	5.51E-9
Periconia macrospinosa	PVI05090.1	TTGATAPFTSLIESV	8.19E-21	LLIQYGTA	3.66E-13	HAGSGTI	4.85E-20	VVPNKQLLDNHQ	3.94E-13	ELADAMERSRYL	4.65E-9
Botryosphaeria dothidea	KAF4303495.1	TIGATATFDDLVRAC	1.98E-13	LLVQYGKN	3.52E-11	HAGSGSI	1.98E-13	VVPNPTLLDNHQ	2.27E-15	ELAEILEQQGYV	6.23E-10
Neofusicoccum parvum	EOD43149.1	TIGATATFDALITAC	7.47E-18	LLVQYGNN	4.08E-15	HAGSGSI	4.34E-21	VVPNPTLLDNHQ	2.27E-15	ELAEILEQQGYV	6.23E-10
Macrophomina phaseolina	EKG10336.1	TIGATATFDELVRAC	2.99E-18	LLVQYGKN	1.92E-17	HAGSGSI	1.23E-19	VVPNPTLLDNHQ	2.27E-15	ELAEILEQQGYV	6.23E-10
Aureobasidium melanogenum	XP_040877796.1	TIGATASFSALIRAT	7.22E-19	LIVQYGAD	4.08E-15	HAGSGTI	2.42E-18	VVPNSELLDNHQ	3.55E-13	ELADALADQEYV	9.07E-9
Mytilinidion resinicola	XP_033579010.1	TVGATASFNSLISAT	2.82E-19	LLVQYGKD	4.53E-19	HAGSGTI	2.51E-19	VVPNEDLLDNHQ	1.37E-13	ELAKVLEEQGYV	2.77E-10
Lophium mytilinum	KAF2488524.1	TVGATASFNSLISAT	2.82E-19	LLLQYGKE	1.3E-16	HAGSGTI	2.51E-19	VVPNEELLDNHQ	1.76E-13	ELAKVLEEQGYV	2.77E-10
Glonium stellatum	OCL07134.1	TIGATAAFNSLISAC	2.61E-18	LLIQYGKE	1.45E-15	HAGSGSI	9.27E-19	VVPNPDLLDNHQ	1.34E-14	ELAEELESQGYV	4.65E-11
Zopfia rhizophila	KAF2193744.1	TTGATAPFTDLIKAV	2.82E-19	LVQYGTA	7.31E-13	HAGSGSI	4.85E-20	VVPNDRLLHNHQ	6.11E-12	NF	
Aspergillus awamori	GCB26403.1	TVGATASFHLLLQSV	2.57E-20	LLLQFGKD	5.94E-15	HAGSGSI	1.23E-19	VVPNPTLKDNHQ	3.05E-15	ELARELQEQGYV	6.65E-11
Aspergillus brasiliensis	OJJ74616.1	TVGATASFHLLLQSV	2.57E-20	LLVQYGKD	6.77E-18	HAGSGSI	9.18E-18	VVPNPTLKDNHQ	3.05E-15	ELAQELQKQGYV	5.57E-12

Aspergillus carbonarius	OOF95088.1	**TVGATASFHLLIQSI**	1.72E-18	**LLIQYGKD**	1.92E-17	**HAGSGSI**	1.23E-19	**VVPNPTLKDNHQ**	3.05E-15	**ELAQELQKQGYV**	5.57E-12
Aspergillus carlsbadensis	KAJ0426393.1	**TVGATASFHLLLESL**	8.48E-18	**LLIQYGKD**	1.75E-16	**HAGSGSI**	4.30E-19	**VVPNPTLQDNHQ**	7.41E-14	**ELADVLQEEGYA**	3.78E-10
Aspergillus costaricensis	XP_025534806.1	**TVGATASFHLLLQSI**	2.55E-17	**LLVQYGKD**	6.77E-18	**HAGSGSI**	1.23E-19	**VVPNPTLKDNHQ**	3.05E-15	**ELAQELQKQGYV**	5.57E-12
Aspergillus eucalypticola	XP_025389724.1	**TVGATASFRLLLQSV**	2.57E-20	**LLVQYGKD**	6.77E-18	**HAGSGSI**	1.23E-19	**VVPNPTLKDNHQ**	3.05E-15	**ELAQELQKQGYV**	5.57E-12
Aspergillus hancockii	KAF7593079.1	**TVGATASFHLLLQGV**	7.22E-19	**LLVQYGKD**	1.85E-18	**HAGSGSI**	1.23E-19	**VVPNTTLKDNHQ**	3.05E-15	**ELARELHNQGYA**	2.03E-10
Aspergillus homomorphus	XP_025547265.1	**TVGATASFNLLIQSI**	5.75E-20	**LLIQYGQD**	3.93E-18	**HAGSGSI**	5.69E-20	**VVPNPTLKDNHQ**	3.05E-15	**ELADILQEQGYL**	7.57E-10
Aspergillus ibericus	XP_025575496.1	**TVGATASFHLLLQSV**	8.38E-19	**LLVQYGKD**	8.2E-17	**HAGSGSI**	1.23E-19	**VVPNPTLKDNHQ**	3.05E-15	**ELAQELQTQGYV**	9.39E-11
Aspergillus luchuensis	XP_041539789.1	**TVGATASFHLLLQSV**	2.57E-20	**LLVQYGKD**	6.77E-18	**HAGSGSI**	1.23E-19	**VVPNPTLKDNHQ**	3.05E-15	**ELAQELQKQGYV**	5.57E-12
Aspergillus mulundensis	XP_026604170.1	**TVGATASFHLLLKSI**	3.32E-19	**LLIQYGKD**	6.77E-18	**HAGSGSI**	1.70E-18	**VVPNPTLQDNHQ**	7.41E-14	**ELAEVLQDEGYA**	2.77E-10
Aspergillus neoniger	XP_025476319.1	**TVGATASFHFLLQSI**	1.3E-18	**LLVQYGKD**	6.77E-18	**HAGSGSI**	1.23E-19	**VVPNPTLKDNHQ**	3.05E-15	**ELAQELQKQGYV**	5.57E-12
Aspergillus niger	EHA26758.1	**TVGATASFHLLLQSV**	2.57E-20	**LLLQYGKD**	3.64E-16	**HAGSGSIL**	1.23E-19	**VVPNPTLKDNHQ**	3.05E-15	**ELARELQEQGYV**	6.65E-11
Aspergillus piperis	XP_025512668.1	**TVGATASFHLLLQSV**	2.57E-20	**LLVQYGKD**	1.75E-16	**HAGSGSI**	1.23E-19	**VVPNPTLKDNHQ**	3.05E-15	**ELAQELQKQGYV**	5.57E-12
Aspergillus sclerotiicarbonarius	PYI12272.1	**TVGATASFHLLIQSI**	1.72E-18	**LLIQFGKD**	4.84E-16	**HAGSGSI**	1.23E-19	**VVPNPTLKDNHQ**	3.05E-15	**ELAQELQKQGYV**	5.57E-12
Aspergillus sclerotioniger	XP_025471984.1	**TVGATASFHLLLHSI**	2.27E-17	**LLIQYGKD**	7.02E-17	**HAGSGSI**	1.23E-19	**VVPNPTLKDNHQ**	3.05E-15	**ELAQELQKQGYV**	5.57E-12
Aspergillus tubingensis	XP_035356720.1	**TVGATASFHLLLQSV**	2.57E-20	**LLVQYGKD**	6.77E-18	**HAGSGSI**	1.23E-19	**VVPNPTLKDNHQ**	3.05E-15	**ELAQELQKQGYV**	5.57E-12
Aspergillus versicolor	XP_040664242.1	**TVGATASFHFLLKSI**	1.98E-18	**LLIQFGKD**	3.15E-16	**HAGSGSI**	4.30E-19	**VVPNPTLQDNHQ**	7.41E-14	**ELAEVLQDEGYA**	2.77E-10
Aspergillus welwitschiae	XP_026629939.1	**TVGATASFHLLLQSV**	2.57E-20	**LLLQFGKD**	5.94E-15	**HAGSGSI**	1.23E-19	**VVPNPTLKDNHQ**	3.05E-15	**ELARELQEQGYV**	6.65E-11
Penicilliopsis zonata	XP_022583757.1	**TVGATASFEPLVSAV**	2.4E-19	**LLVQYGKD**	1.3E-16	**HAGSGSI**	3.05E-18	**VVPNSSLKDNHQ**	2.40E-13	**ELARELQKQGYV**	5.57E-12
Penicillium alfredii	XP_056508930.1	**TVGATASFHGLLQQV**	6.95E-20	**LLIQYGRD**	8.2E-17	**HAGTGTI**	1.05E-18	**VVPNPDLADNHQ**	4.74E-13	**ELATVLDKQNYV**	4.27E-9
Penicillium bovifimosum	XP_056523126.1	**TVGATAAFRKLLEQV**	1.44E-19	**FLVQYGKD**	4.39E-14	**HAGTGTI**	3.76E-19	**VVPNPDLADNHQ**	4.74E-13	**ELADVLEEDKYV**	1.89E-7
Penicillium macrosclerotiorum	XP_056932270.1	**TVGATASFEKLVQEV**	1.66E-20	**LIVQYGKN**	5.57E-16	**HAGTGSI**	2.42E-18	**VVPNPDLADNHQ**	4.74E-13	**ELALELANYGYA**	9.07E-9
Penicillium odoratum	XP_057001022.1	**TVGATAPFPDLIRAV**	2.4E-19	**LIIQHGTY**	4.94E-13	**HAGTGTI**	1.23E-19	**IVPNPALANNHQ**	4.36E-12	**ELAMQMENLGYG**	1.54E-7
Talaromyces amestolkiae	XP_040733191.1	**TIGATAPFDALLSNV**	9.72E-19	**LLIQYGKE**	1.12E-16	**HAGSGSI**	4.28E-18	**VVPNPALQDNHQ**	3.25E-13	**ELARQIAKNGWA**	9.07E-9
Talaromyces atroroseus	XP_020124160.1	**TIGATAPFDTLLSNV**	3.9E-18	**LLIQYGKE**	1.11E-15	**HAGSGSI**	4.28E-18	**VVPNPALQGNHQ**	2.03E-12	**ELAKEIENNGWA**	9.18E-10
Cladophialophora carrionii	XP_008726825.1	**TVGATAPFNSLVRAV**	4.73E-20	**LQIQYGDQ**	8.05E-13	**HAGSGSI**	1.22E-21	**VVPNTDLLHNHQ**	3.94E-13	**ELAEALAEQEYV**	5.06E-9
Cladophialophora chaetospira	KAJ9603522.1	**TVGATAPFNSLVRAV**	4.73E-20	**LRIQYGDQ**	2.22E-14	**HAGSGSI**	1.21E-20	**VVPNTDLLHNHQ**	3.94E-13	**ELAEALAEQEYV**	5.06E-9
Phaeomoniella chlamydospora	KKY25069.1	**TIGATAAFNSLLKAA**	6.57E-18	**LLLQYGSD**	1.65E-15	**HAGSGSI**	2.10E-21	**VVPNTDLLHNHQ**	3.94E-13	**ELAEELAKQDYV**	3.41E-10

Botryotinia calthae	TEY39080.1	TVGATATFKELIEEV	8.48E-18	LRVQAGPD	5.25E-15	HAGSGTI	4.86E-22	VVPNTSLLDNHQ	8.97E-15	ELADELERQGYV	6.43E-12
Botryotinia convoluta	TGO61616.1	TVGATATFKELIEEV	1.3E-18	LRVQAGPD	8.58E-15	HAGSGTI	4.86E-22	VVPNTSLLDNHQ	8.97E-15	ELADELERQGYV	6.43E-12
Botryotinia globosa	KAF7896435.1	TVGATAPFKELIEEV	2.4E-19	LRVQAGPD	3.13E-14	HAGSGTI	4.86E-22	VVPNTSLLDNHQ	8.97E-15	ELADELERQGYV	6.43E-12
Botryotinia narcissicola	TGO69167.1	TVGATATFKELIEEV	1.3E-18	LRVQAGPD	1.56E-14	HAGSGTI	4.86E-22	VVPNTSLLDNHQ	8.97E-15	ELADELERQGYV	6.43E-12
Botrytis aclada	KAF7946254.1	TVGATATFKELIEEV	1.3E-18	LRVQAGPD	4.39E-14	HAGSGTI	4.86E-22	VVPNTSLLDNHQ	8.97E-15	ELADELERQGYV	6.43E-12
Botrytis byssoidea	XP_038729320.1	TVGATATFKELIEEV	1.3E-18	LRVQAGPD	1.45E-14	HAGSGTI	4.86E-22	VVPNTSLLDNHQ	8.97E-15	ELADELERQGYV	6.43E-12
Botrytis cinerea	XP_001557717.1	TVGATATFKELIEEV	1.3E-18	LRVQAGPD	5.25E-15	HAGSGTI	6.86E-23	VVPNTSLLDNHQ	8.97E-15	ELADELERQGYV	6.43E-12
Botrytis deweyae	XP_038812155.1	TVGATATFKELIEEV	1.3E-18	LRVQAGPD	8.58E-15	HAGSGTI	4.86E-22	VVPNTSLLDNHQ	8.97E-15	ELADELERQGYV	6.43E-12
Botrytis fragariae	XP_037188780.1	TVGATATFKELIEEV	1.3E-18	LRVQAGPD	8.58E-15	HAGSGTI	4.86E-22	VVPNTSLLDNHQ	8.97E-15	ELADELERQGYV	6.43E-12
Botrytis galanthina	THV55011.1	TVGATATFKELIEEV	1.3E-18	LRVQAGPD	1.56E-14	HAGSGTI	4.86E-22	VVPNTSLLDNHQ	8.97E-15	ELADELERQGYV	6.43E-12
Botrytis paeoniae	TGO28479.1	TVGATATFKELIEEV	1.3E-18	LRVQAGPD	8.58E-15	HAGSGTI	4.86E-22	VVPNTSLLDNHQ	8.97E-15	ELADELERQGYV	6.43E-12
Botrytis porri	XP_038770654.1	TVGATATFKELIEEV	1.3E-18	LRVQAGPD	5.25E-15	HAGSGTI	1.22E-21	VVPNTSLLDNHQ	8.97E-15	ELADELERQGYV	6.43E-12
Botrytis sinoallii	XP_038758083.1	TVGATATFKELIEEV	1.3E-18	LRVQAGPD	8.58E-15	HAGSGTI	4.86E-22	VVPNTSLLDNHQ	8.97E-15	ELADELERQGYV	6.43E-12
Botrytis tulipae	TGO11019.1	TVGATATFKELIEEV	1.3E-18	LRVQAGPD	1.56E-14	HAGSGTI	4.86E-22	VVPNTSLLDNHQ	8.97E-15	ELADELERQGYV	6.43E-12
Sclerotinia borealis	ESZ92680.1	TVGATATFKELIEEV	1.3E-18	LRIQAGPD	1.61E-13	HAGSGTI	6.78E-22	VVPNTSLLDNHQ	8.97E-15	QLADELERQGYV	3.41E-10
Sclerotinia nivalis	KAJ8059674.1	TVGATATFKELIEEV	1.3E-18	LRVQAGPD	5.25E-15	HAGSGTI	4.86E-22	VVPNTSLLDNHQ	8.97E-15	ELADELERQGYV	6.43E-12
Sclerotinia sclerotiorum	XP_001588350.1	TVGATATFKELIEEV	1.3E-18	LRVQAGLD	5.45E-13	HAGSGTI	6.86E-23	VVPNTSLLDNHQ	8.97E-15	ELADELERQGYV	6.43E-12
Sclerotinia trifoliorum	CAD6445273.1	TVGATATFKELIEEV	1.3E-18	LRVQAGPD	5.25E-15	HAGSGTI	6.86E-23	VVPNTSLLDNHQ	8.97E-15	ELADELERQGYV	6.43E-12
Monilinia laxa	KAB8300938.1	TVGATATFKELIEEV	1.3E-18	LRVQAGPD	1.76E-14	HAGSGTI	6.78E-22	VVPNTSLLDNHQ	8.97E-15	ELADELERQGYV	6.43E-12
Stromatinia cepivora	KAF7858421.1	TVGATATFKELIEEV	1.3E-18	LRVQAGPD	5.25E-15	HAGSGTI	4.86E-22	VVPNTSLLDNHQ	8.97E-15	ELADELERQGYV	6.43E-12
Rhexocercosporidium sp. MPI-PUGE-AT-0058	KAH7350870.1	TTGATAKFTELIQAA	1.88E-15	LNVQCGES	4.67E-12	HAGAGTI	1.92E-18	VVPNSSLLDNHQ	1.93E-13	ELAAELETQGYA	4.18E-10
Pseudogymnoascus destructans	XP_024324285.1	TLGATAPFPALLSAS	4.99E-14	LTLQCGAD	9.67E-16	HAGAGTA	8.21E-16	LVPNPALLDNHQ	1.89E-12	ELAKELDRMGYA	1.64E-10
Pseudogymnoascus verrucosus	XP_018130987.1	TLGATAPFPTLLSAS	1.1E-13	LTLQCGAD	9.67E-16	HAGAGTA	8.21E-16	LVPNPALLDNHQ	1.89E-12	ELAKELDKIGYA	3.41E-10
Claussenomyces sp. TS43310	KAI9740875.1	TIGATASFRALVDAT	6.43E-17	LTLQCGPD	6.85E-19	HAGAGTI	1.42E-19	VVPNPELLDNHQ	2.36E-14	ELAEELERQGYV	3.05E-12
Alectoria fallacina	CAF9938699.1	TVGATASFDSLIRAT	1.71E-19	LRLQHGKN	1.97E-14	HAGSGSI	2.95E-20	VVPNPDLLDNHQ	1.34E-14	ELAEELAIQGYV	3.07E-10
Alectoria sarmentosa	CAD6567071.1	TVGATASFDSLIRAS	9.72E-19	LRLQHGKD	3.6E-15	HAGSGSI	2.48E-20	VVPNPDLLDNHQ	1.34E-14	ELAEELATQGYV	5.91E-11
Lasallia pustulata	KAA6414625.1	TIGATASFDALIKEA	2.4E-19	LLLQHGKE	5.48E-14	HAGSGSI	2.48E-20	VVPNPDLLDNHQ	1.34E-14	ELAEELSNQGYV	2.81E-11

Pycnora praestabilis	KAI9813616.1	**TIGATASFDSLLKAV**	2.57E-20	**LLLQYGKE**	1.75E-16	**HAGSGSI**	2.37E-22	**VVPNPELLDNHQ**	2.36E-14	**ELAEELAAQGYV**	1.64E-10
Xylona heveae	XP_018185144.1	**TIGATASFDALISAV**	1.66E-20	**LLVQYGKE**	2.67E-17	**HAGSGSI**	2.48E-20	**VVPNPSLLNNHQ**	2.36E-14	**ELAEELASMGYV**	1.34E-9

Accession number: Unique identifier assigned to each biological sequence within the NCBI database. **p-value:** Probability that a random sequence (of the same length) would match the test motif with a score equal to or higher than the highest found in the test sequence. **NF**: Not found.

Figure S1. Motifs location types in putative Rht1 sequences.

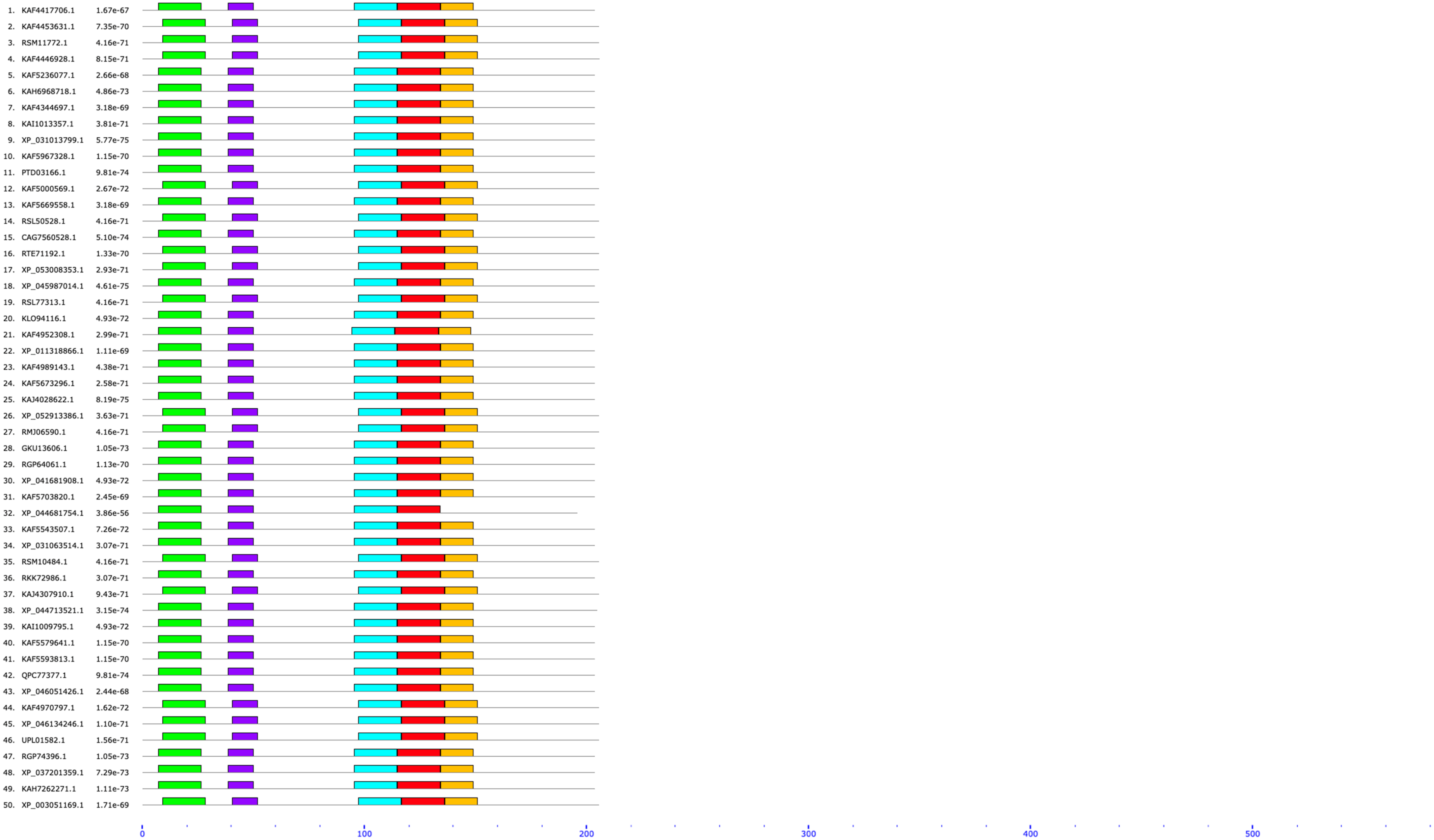

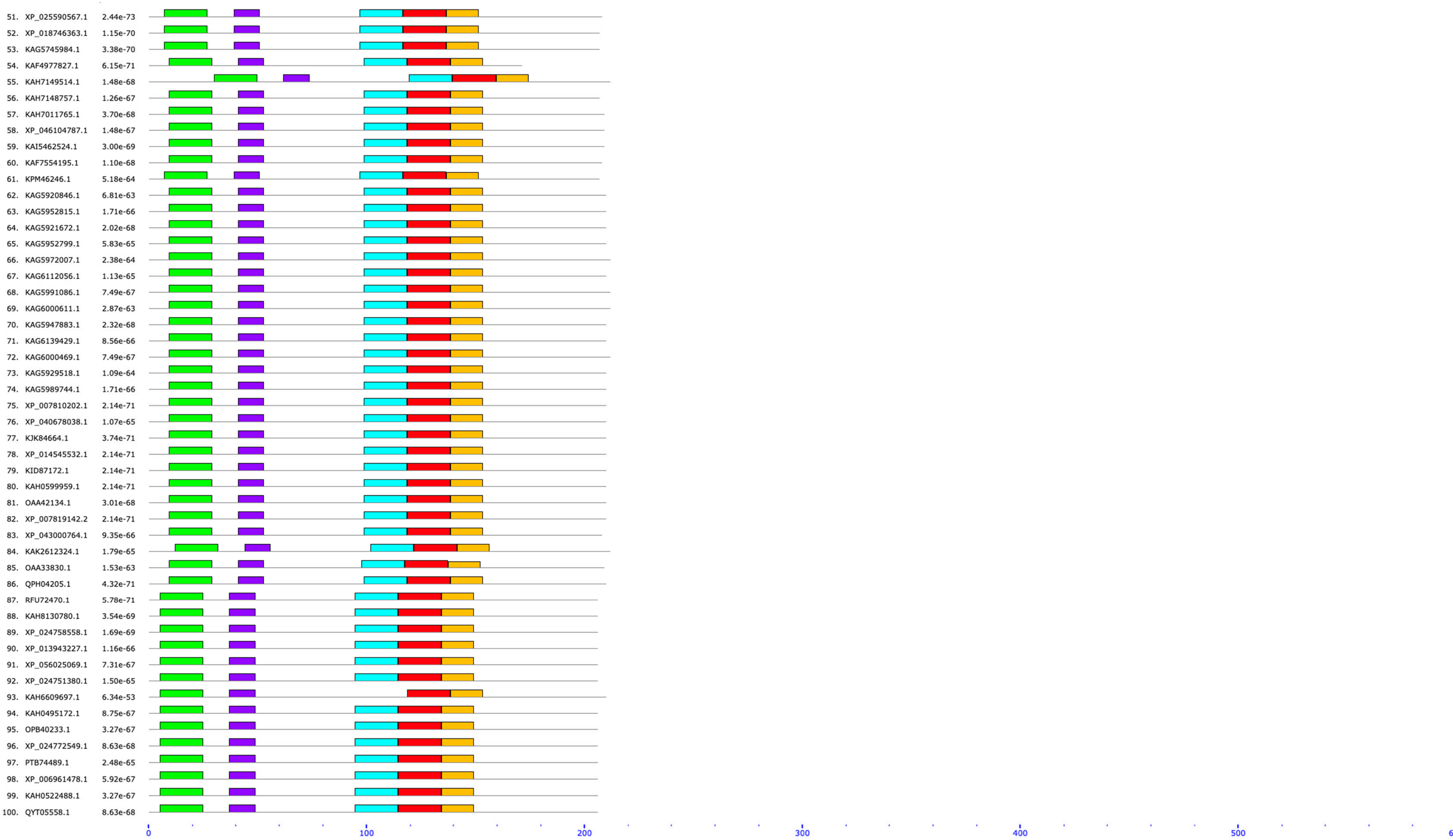
51. XP_025590567.1 2.44e-73
52. XP_018746363.1 1.15e-70
53. KAG5745984.1 3.38e-70
54. KAF4977827.1 6.15e-71
55. KAH7149514.1 1.48e-68
56. KAH7148757.1 1.26e-67
57. KAH7011765.1 3.70e-68
58. XP_046104787.1 1.48e-67
59. KAI5462524.1 3.00e-69
60. KAF7554195.1 1.10e-68
61. KPM46246.1 5.18e-64
62. KAG5920846.1 6.81e-63
63. KAG5952815.1 1.71e-66
64. KAG5921672.1 2.02e-68
65. KAG5952799.1 5.83e-65
66. KAG5972007.1 2.38e-64
67. KAG6112056.1 1.13e-65
68. KAG5991086.1 7.49e-67
69. KAG6000611.1 2.87e-63
70. KAG5947883.1 2.32e-68
71. KAG6139429.1 8.56e-66
72. KAG6000469.1 7.49e-67
73. KAG5929518.1 1.09e-64
74. KAG5989744.1 1.71e-66
75. XP_007810202.1 2.14e-71
76. XP_040678038.1 1.07e-65
77. KJK84664.1 3.74e-71
78. XP_014545532.1 2.14e-71
79. KID87172.1 2.14e-71
80. KAH0599959.1 2.14e-71
81. OAA42134.1 3.01e-68
82. XP_007819142.2 2.14e-71
83. XP_043000764.1 9.35e-66
84. KAK2612324.1 1.79e-65
85. OAA33830.1 1.53e-63
86. QPH04205.1 4.32e-71
87. RFU72470.1 5.78e-71
88. KAH8130780.1 3.54e-69
89. XP_024758558.1 1.69e-69
90. XP_013943227.1 1.16e-66
91. XP_056025069.1 7.31e-67
92. XP_024751380.1 1.50e-65
93. KAH6609697.1 6.34e-53
94. KAH0495172.1 8.75e-67
95. OPB40233.1 3.27e-67
96. XP_024772549.1 8.63e-68
97. PTB74489.1 2.48e-65
98. XP_006961478.1 5.92e-67
99. KAH0522488.1 3.27e-67
100. QYT05558.1 8.63e-68
0
100
200
300
400
500
6

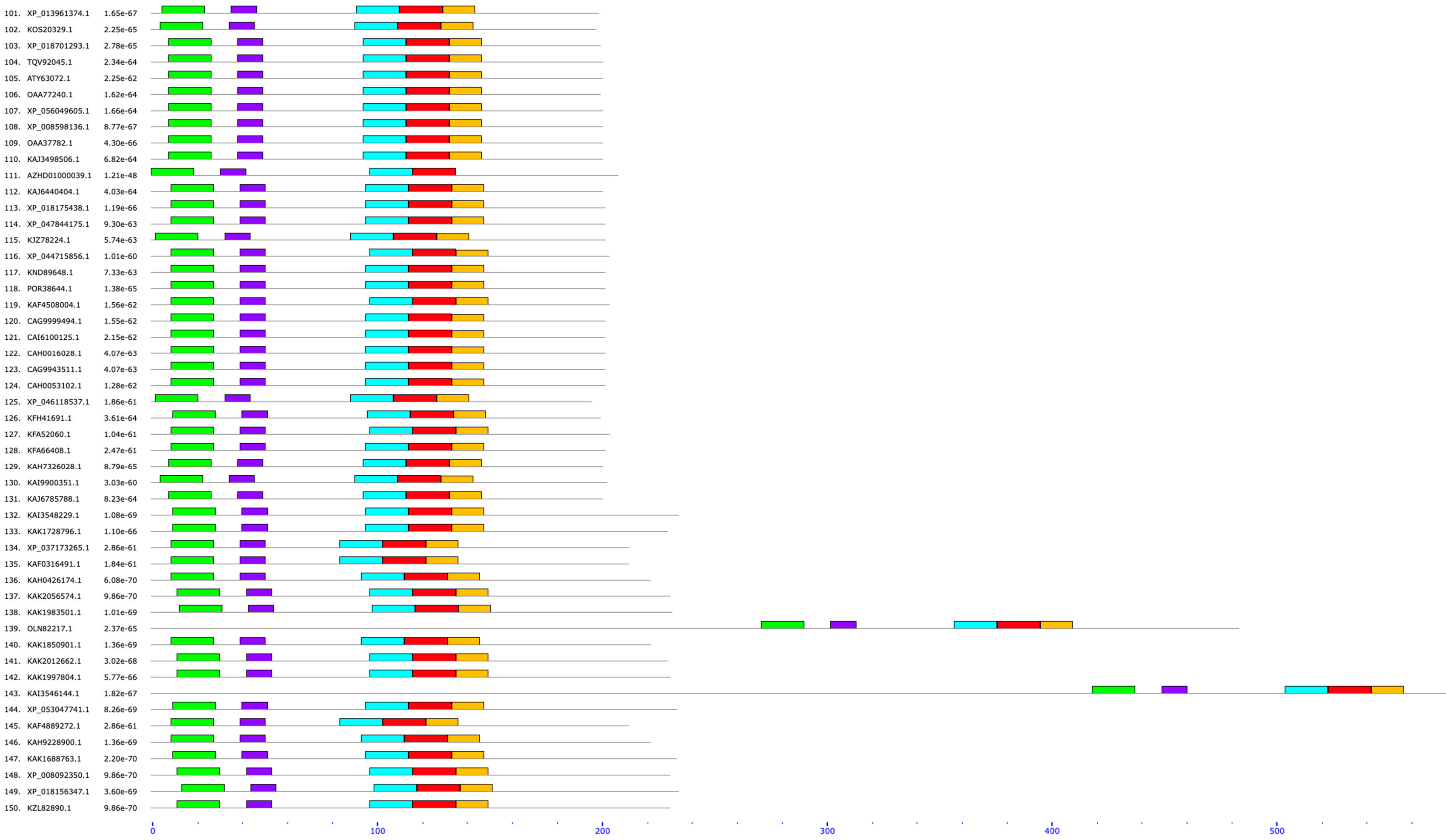
101. XP_013961374.1 1.65e-67
102. KOS20329.1 2.25e-65
103. XP_018701293.1 2.78e-65
104. TQV92045.1 2.34e-64
105. ATY63072.1 2.25e-62
106. OAA77240.1 1.62e-64
107. XP_056049605.1 1.66e-64
108. XP_008598136.1 8.77e-67
109. OAA37782.1 4.30e-66
110. KAJ3498506.1 6.82e-64
111. AZHD01000039.1 1.21e-48
112. KAJ6440404.1 4.03e-64
113. XP_018175438.1 1.19e-66
114. XP_047844175.1 9.30e-63
115. KJZ78224.1 5.74e-63
116. XP_044715856.1 1.01e-60
117. KND89648.1 7.33e-63
118. POR38644.1 1.38e-65
119. KAF4508004.1 1.56e-62
120. CAG9999494.1 1.55e-62
121. CAI6100125.1 2.15e-62
122. CAH0016028.1 4.07e-63
123. CAG9943511.1 4.07e-63
124. CAH0053102.1 1.28e-62
125. XP_046118537.1 1.86e-61
126. KFH41691.1 3.61e-64
127. KFA52060.1 1.04e-61
128. KFA66408.1 2.47e-61
129. KAH7326028.1 8.79e-65
130. KAI9900351.1 3.03e-60
131. KAJ6785788.1 8.23e-64
132. KAI3548229.1 1.08e-69
133. KAK1728796.1 1.10e-66
134. XP_037173265.1 2.86e-61
135. KAF0316491.1 1.84e-61
136. KAH0426174.1 6.08e-70
137. KAK2056574.1 9.86e-70
138. KAK1983501.1 1.01e-69
139. OLN82217.1 2.37e-65
140. KAK1850901.1 1.36e-69
141. KAK2012662.1 3.02e-68
142. KAK1997804.1 5.77e-66
143. KAI3546144.1 1.82e-67
144. XP_053047741.1 8.26e-69
145. KAF4889272.1 2.86e-61
146. KAH9228900.1 1.36e-69
147. KAK1688763.1 2.20e-70
148. XP_008092350.1 9.86e-70
149. XP_018156347.1 3.60e-69
150. KZL82890.1 9.86e-70
0
100
200
300
400
500

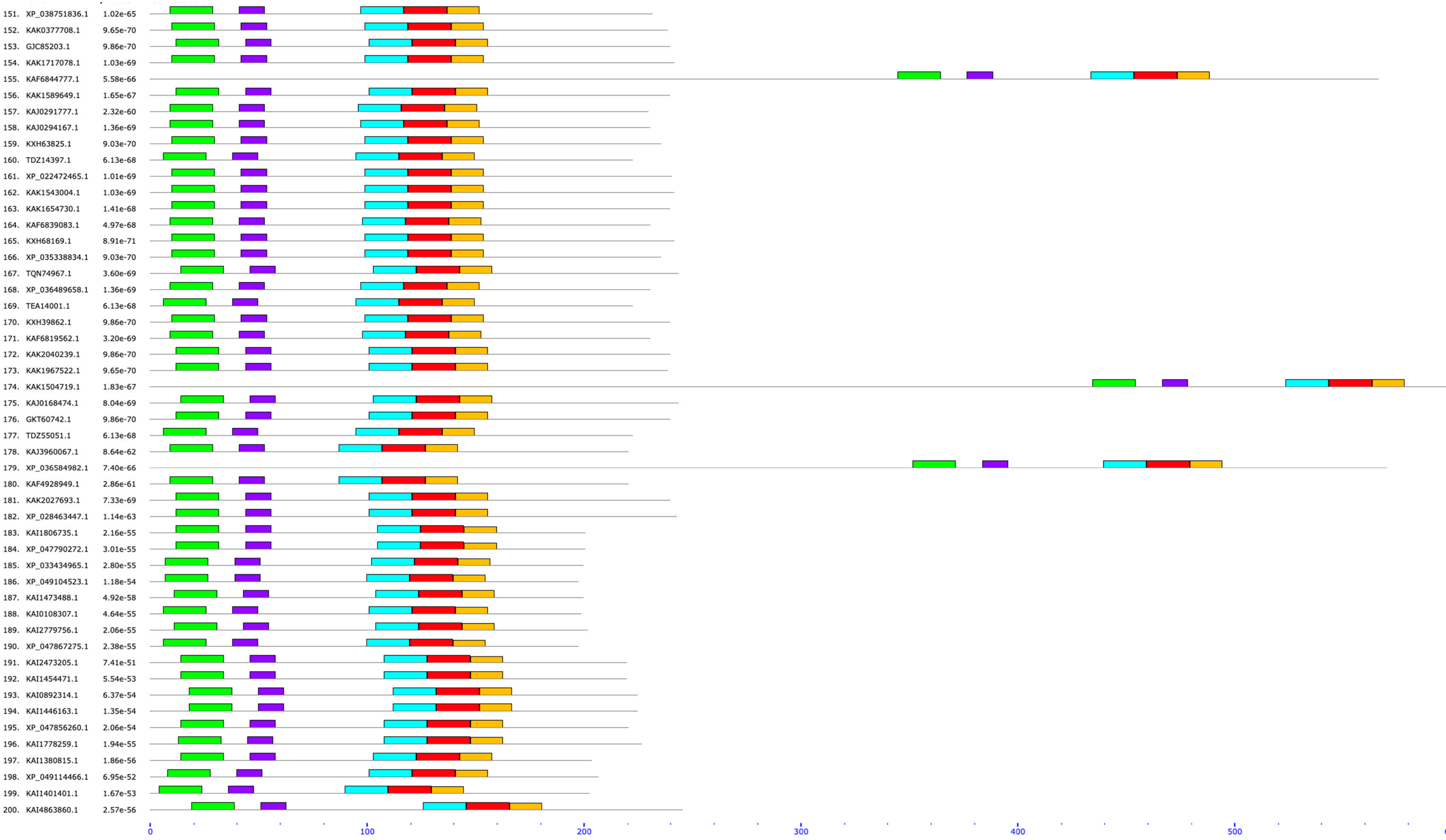
151. XP_038751836.1 1.02e-65
152. KAK0377708.1 9.65e-70
153. GJC85203.1 9.86e-70
154. KAK1717078.1 1.03e-69
155. KAF6844777.1 5.58e-66
156. KAK1589649.1 1.65e-67
157. KAJ0291777.1 2.32e-60
158. KAJ0294167.1 1.36e-69
159. KXH63825.1 9.03e-70
160. TDZ14397.1 6.13e-68
161. XP_022472465.1 1.01e-69
162. KAK1543004.1 1.03e-69
163. KAK1654730.1 1.41e-68
164. KAF6839083.1 4.97e-68
165. KXH68169.1 8.91e-71
166. XP_035338834.1 9.03e-70
167. TQN74967.1 3.60e-69
168. XP_036489658.1 1.36e-69
169. TEA14001.1 6.13e-68
170. KXH39862.1 9.86e-70
171. KAF6819562.1 3.20e-69
172. KAK2040239.1 9.86e-70
173. KAK1967522.1 9.65e-70
174. KAK1504719.1 1.83e-67
175. KAJ0168474.1 8.04e-69
176. GKT60742.1 9.86e-70
177. TDZ55051.1 6.13e-68
178. KAJ3960067.1 8.64e-62
179. XP_036584982.1 7.40e-66
180. KAF4928949.1 2.86e-61
181. KAK2027693.1 7.33e-69
182. XP_028463447.1 1.14e-63
183. KAI1806735.1 2.16e-55
184. XP_047790272.1 3.01e-55
185. XP_033434965.1 2.80e-55
186. XP_049104523.1 1.18e-54
187. KAI1473488.1 4.92e-58
188. KAI0108307.1 4.64e-55
189. KAI2779756.1 2.06e-55
190. XP_047867275.1 2.38e-55
191. KAI2473205.1 7.41e-51
192. KAI1454471.1 5.54e-53
193. KAI0892314.1 6.37e-54
194. KAI1446163.1 1.35e-54
195. XP_047856260.1 2.06e-54
196. KAI1778259.1 1.94e-55
197. KAI1380815.1 1.86e-56
198. XP_049114466.1 6.95e-52
199. KAI1401401.1 1.67e-53
200. KAI4863860.1 2.57e-56
0
100
200
300
400
500

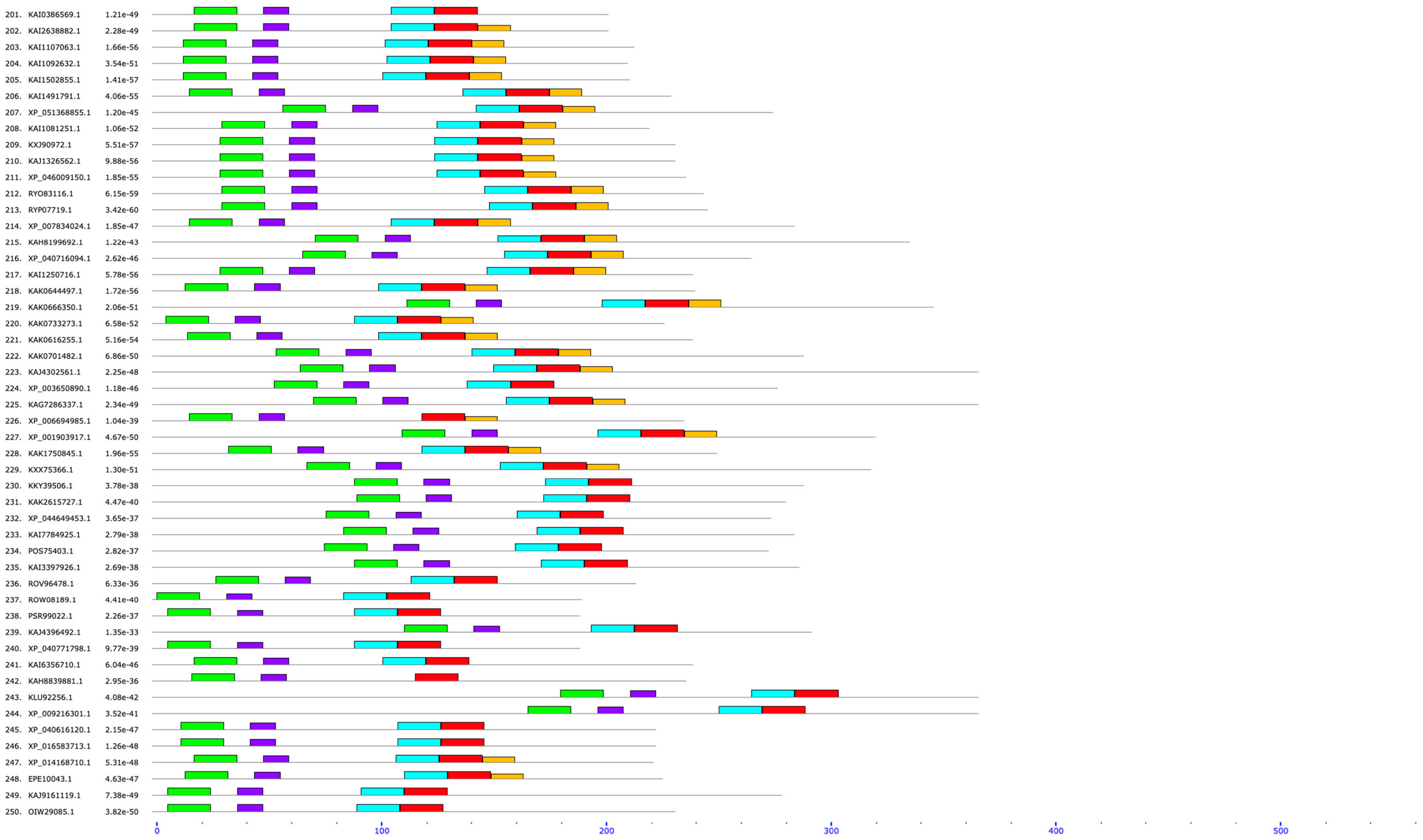
201. KAI0386569.1 1.21e-49
202. KAI2638882.1 2.28e-49
203. KAI1107063.1 1.66e-56
204. KAI1092632.1 3.54e-51
205. KAI1502855.1 1.41e-57
206. KAI1491791.1 4.06e-55
207. XP_051368855.1 1.20e-45
208. KAI1081251.1 1.06e-52
209. KXJ90972.1 5.51e-57
210. KAJ1326562.1 9.88e-56
211. XP_046009150.1 1.85e-55
212. RYO83116.1 6.15e-59
213. RYP07719.1 3.42e-60
214. XP_007834024.1 1.85e-47
215. KAH8199692.1 1.22e-43
216. XP_040716094.1 2.62e-46
217. KAI1250716.1 5.78e-56
218. KAK0644497.1 1.72e-56
219. KAK0666350.1 2.06e-51
220. KAK0733273.1 6.58e-52
221. KAK0616255.1 5.16e-54
222. KAK0701482.1 6.86e-50
223. KAJ4302561.1 2.25e-48
224. XP_003650890.1 1.18e-46
225. KAG7286337.1 2.34e-49
226. XP_006694985.1 1.04e-39
227. XP_001903917.1 4.67e-50
228. KAK1750845.1 1.96e-55
229. KXX75366.1 1.30e-51
230. KKY39506.1 3.78e-38
231. KAK2615727.1 4.47e-40
232. XP_044649453.1 3.65e-37
233. KAI7784925.1 2.79e-38
234. POS75403.1 2.82e-37
235. KAI3397926.1 2.69e-38
236. ROV96478.1 6.33e-36
237. ROW08189.1 4.41e-40
238. PSR99022.1 2.26e-37
239. KAJ4396492.1 1.35e-33
240. XP_040771798.1 9.77e-39
241. KAI6356710.1 6.04e-46
242. KAH8839881.1 2.95e-36
243. KLU92256.1 4.08e-42
244. XP_009216301.1 3.52e-41
245. XP_040616120.1 2.15e-47
246. XP_016583713.1 1.26e-48
247. XP_014168710.1 5.31e-48
248. EPE10043.1 4.63e-47
249. KAJ9161119.1 7.38e-49
250. OIW29085.1 3.82e-50
0
100
200
300
400
500

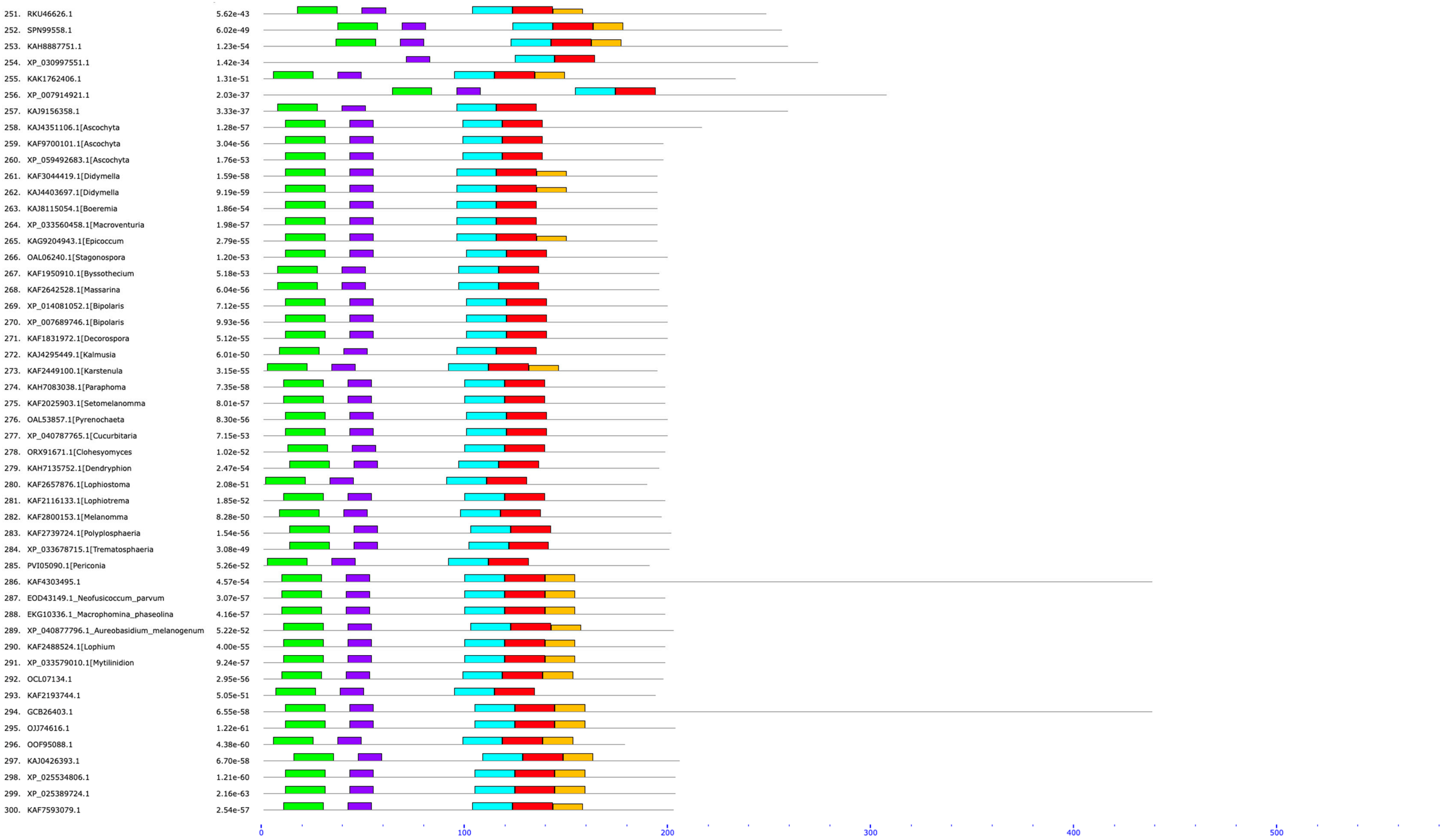

251. RKU46626.1 5.62e-43
252. SPN99558.1 6.02e-49
253. KAH8887751.1 1.23e-54
254. XP_030997551.1 1.42e-34
255. KAK1762406.1 1.31e-51
256. XP_007914921.1 2.03e-37
257. KAJ9156358.1 3.33e-37
258. KAJ4351106.1[Ascochyta 1.28e-57
259. KAF9700101.1[Ascochyta 3.04e-56
260. XP_059492683.1[Ascochyta 1.76e-53
261. KAF3044419.1[Didymella 1.59e-58
262. KAJ4403697.1[Didymella 9.19e-59
263. KAJ8115054.1[Boeremia 1.86e-54
264. XP_033560458.1[Macroventuria 1.98e-57
265. KAG9204943.1[Epicoccum 2.79e-55
266. OAL06240.1[Stagonospora 1.20e-53
267. KAF1950910.1[Byssothecium 5.18e-53
268. KAF2642528.1[Massarina 6.04e-56
269. XP_014081052.1[Bipolaris 7.12e-55
270. XP_007689746.1[Bipolaris 9.93e-56
271. KAF1831972.1[Decorospora 5.12e-55
272. KAJ4295449.1[Kalmusia 6.01e-50
273. KAF2449100.1[Karstenula 3.15e-55
274. KAH7083038.1[Paraphoma 7.35e-58
275. KAF2025903.1[Setomelanomma 8.01e-57
276. OAL53857.1[Pyrenochaeta 8.30e-56
277. XP_040787765.1[Cucurbitaria 7.15e-53
278. ORX91671.1[Clohesyomyces 1.02e-52
279. KAH7135752.1[Dendryphion 2.47e-54
280. KAF2657876.1[Lophiostoma 2.08e-51
281. KAF2116133.1[Lophiotrema 1.85e-52
282. KAF2800153.1[Melanomma 8.28e-50
283. KAF2739724.1[Polyplosphaeria 1.54e-56
284. XP_033678715.1[Trematosphaeria 3.08e-49
285. PVI05090.1[Periconia 5.26e-52
286. KAF4303495.1 4.57e-54
287. EOD43149.1_Neofusicoccum_parvum 3.07e-57
288. EKG10336.1_Macrophomina_phaseolina 4.16e-57
289. XP_040877796.1_Aureobasidium_melanogenum 5.22e-52
290. KAF2488524.1[Lophium 4.00e-55
291. XP_033579010.1[Mytilinidion 9.24e-57
292. OCL07134.1 2.95e-56
293. KAF2193744.1 5.05e-51
294. GCB26403.1 6.55e-58
295. OJJ74616.1 1.22e-61
296. OOF95088.1 4.38e-60
297. KAJ0426393.1 6.70e-58
298. XP_025534806.1 1.21e-60
299. XP_025389724.1 2.16e-63
300. KAF7593079.1 2.54e-57
0
100
200
300
400
500

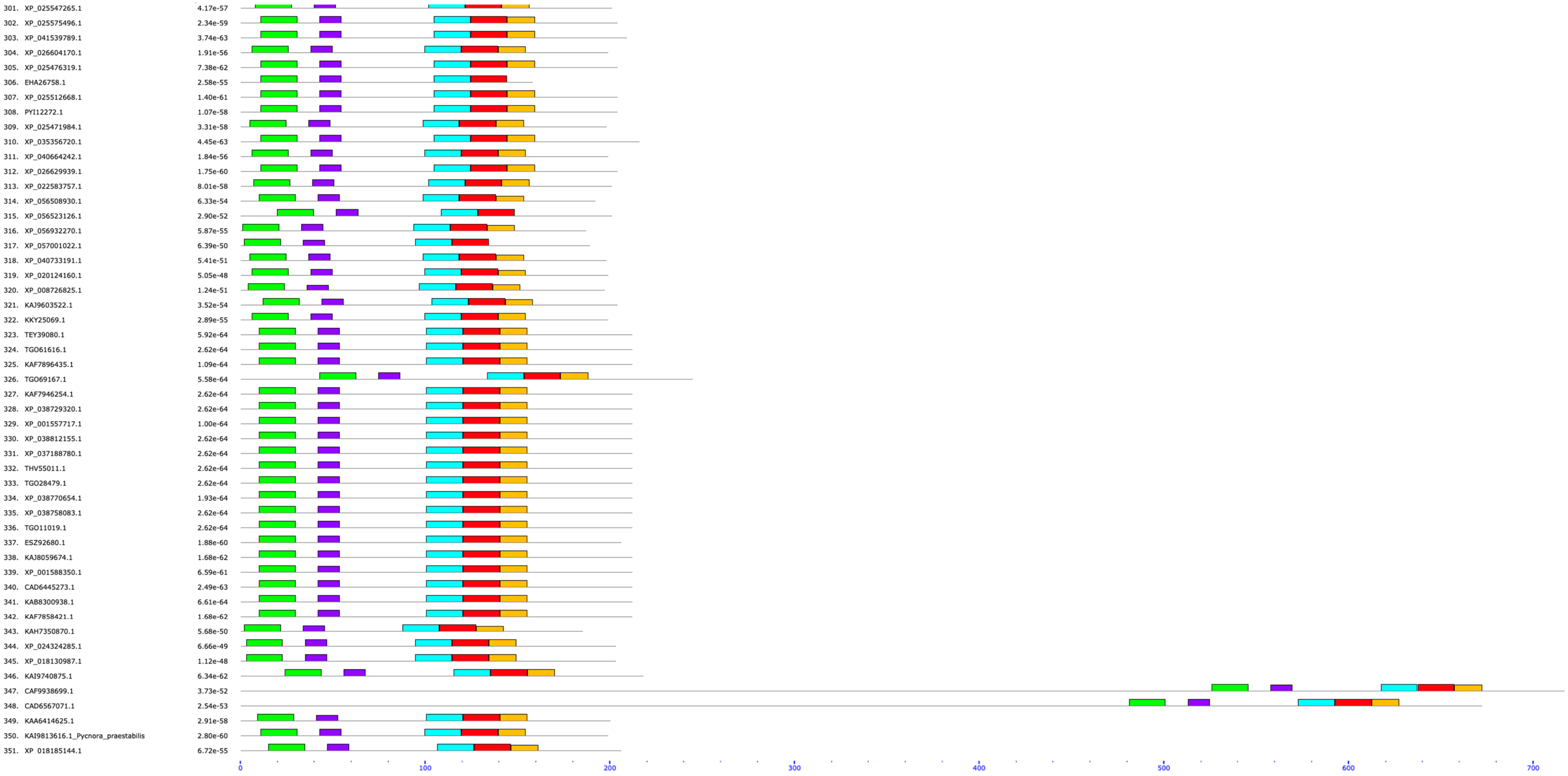

301. XP_025547265.1 4.17e-57
302. XP_025575496.1 2.34e-59
303. XP_041539789.1 3.74e-63
304. XP_026604170.1 1.91e-56
305. XP_025476319.1 7.38e-62
306. EHA26758.1 2.58e-55
307. XP_025512668.1 1.40e-61
308. PYI12272.1 1.07e-58
309. XP_025471984.1 3.31e-58
310. XP_035356720.1 4.45e-63
311. XP_040664242.1 1.84e-56
312. XP_026629939.1 1.75e-60
313. XP_022583757.1 8.01e-58
314. XP_056508930.1 6.33e-54
315. XP_056523126.1 2.90e-52
316. XP_056932270.1 5.87e-55
317. XP_057001022.1 6.39e-50
318. XP_040733191.1 5.41e-51
319. XP_020124160.1 5.05e-48
320. XP_008726825.1 1.24e-51
321. KAJ9603522.1 3.52e-54
322. KKY25069.1 2.89e-55
323. TEY39080.1 5.92e-64
324. TGO61616.1 2.62e-64
325. KAF7896435.1 1.09e-64
326. TGO69167.1 5.58e-64
327. KAF7946254.1 2.62e-64
328. XP_038729320.1 2.62e-64
329. XP_001557717.1 1.00e-64
330. XP_038812155.1 2.62e-64
331. XP_037188780.1 2.62e-64
332. THV55011.1 2.62e-64
333. TGO28479.1 2.62e-64
334. XP_038770654.1 1.93e-64
335. XP_038758083.1 2.62e-64
336. TGO11019.1 2.62e-64
337. ESZ92680.1 1.88e-60
338. KAJ8059674.1 1.68e-62
339. XP_001588350.1 6.59e-61
340. CAD6445273.1 2.49e-63
341. KAB8300938.1 6.61e-64
342. KAF7858421.1 1.68e-62
343. KAH7350870.1 5.68e-50
344. XP_024324285.1 6.66e-49
345. XP_018130987.1 1.12e-48
346. KAI9740875.1 6.34e-62
347. CAF9938699.1 3.73e-52
348. CAD6567071.1 2.54e-53
349. KAA6414625.1 2.91e-58
350. KAI9813616.1_Pycnora_praestabilis 2.80e-60
351. XP_018185144.1 6.72e-55
0
100
200
300
400
500
600
700

Table S3. Identified motifs in putative Rht2.

Species	Accession numbers	Motif 1	*p*-value	Motif 2	*p*-value	Motif 3	*p*-value	Motif 4	*p*-value	Motif 5	*p*-value
Fusarium acutatum	KAF4435765.1	**TQGTLAMDPTSLIIPS**	9.69E-20	**PPNVRFIRWIPY**	8.83E-12	**TNGGYGSITQ**	1.02E-19	**VPLICAGQTEDK**	5.68E-14	**RVSWARAGIDLK**	1.98E-11
Fusarium albosucccineum	KAF4470643.1	**TQGTIARDYTDLLIPS**	5.57E-18	**PSNTRVIDYLPY**	1.62E-14	**MNAGYGGFLH**	5.77E-20	**VPLVLAGETEDK**	3.69E-13	**RGQWSGVAANLR**	6.23E-11
Fusarium ambrosium	RSL97308.1	**TQGTLAMDPTSLIIPA**	3.17E-20	**NF**		**TNGGYGSVTQ**	1.53E-17	**VPLICAGQTEDK**	5.68E-14	**RVSWAGAGIDLK**	1.21E-12
Fusarium austroafricanum	KAF4445897.1	**TQGTLAMDPTSLIIPA**	3.17E-20	**VPNVRYIKWIPY**	4.88E-10	**TNGGYGSITQ**	1.02E-19	**VPLICAGRTEDK**	1.50E-12	**RVSWAKAGIDLK**	1.93E-10
Fusarium austroamericanum	KAF5233753.1	**TQGTLAMDPTSLIIPA**	3.17E-20	**LPNVRYVKWIPY**	1.46E-10	**TNGGYGSITQ**	1.89E-17	**IPLICAGQSEDK**	3.24E-12	**RVDWAGAGIDLK**	4.13E-12
Fusarium avenaceum	KIL89965.1	**TQGTLAMDPTSLIIPA**	3.17E-20	**PSNVRFIKWIPY**	2.56E-12	**TNGGYGSITQ**	1.02E-19	**VPLICAGQTEDK**	5.68E-14	**RVSWARAGIDLK**	1.98E-11
Fusarium beomiforme	KAF4334837.1	**TQGTIARDVTKLIIPT**	3.40E-16	**PPNSRVIDYLPY**	4.51E-12	**MNAGYGGFLH**	4.66E-18	**VPLVLAGETEDK**	3.69E-13	**RGEWSGVAVNLK**	2.65E-13
Fusarium chuoi	KAI1019319.1	**TQGTLAMDPTSLIIPA**	3.17E-20	**PPNVRFIRWIPY**	8.83E-12	**TNGGYGSITQ**	1.02E-19	**VPLICAGQTEDK**	5.68E-14	**RVSWARAGIDLK**	1.98E-11
Fusarium coffeatum	XP_031011366.1	**TQGTLAMDPTSLIIPA**	3.17E-20	**LPNVRYVKWIPY**	1.46E-10	**TNGGYGSITQ**	1.02E-19	**VPLICAGQTEDK**	5.68E-14	**RVGWAGAGIDLK**	2.52E-11
Fusarium coicis	KAF5967796.1	**TQGTLAMDPTSLIIPA**	3.17E-20	**PPNVRFIRWIPY**	8.83E-12	**TNGGYGSITQ**	1.02E-19	**VPLICAGQTEDK**	5.68E-14	**RVSWARAGIDLK**	1.98E-11
Fusarium culmorum	PTD08619.1	**TQGTLAMDPTSLIIPA**	3.17E-20	**LPNVRYVKWIPY**	1.46E-10	**TNGGYGSITQ**	1.02E-19	**VPLICAGQSEDK**	2.25E-13	**RVDWAGAGIDLK**	4.13E-12
Fusarium decemcellulare	KAF4990227.1	**TQGTLAMDPTSLIIPA**	3.17E-20	**LPNVRFAKFIPY**	1.79E-10	**TNGGYGSITQ**	1.02E-19	**VPLICAGQTEDK**	5.68E-14	**RISWAGAGIDLK**	6.27E-12
Fusarium denticulatum	KAF5676768.1	**TQGTLAMDPTSLIIPA**	3.17E-20	**PPNVRFIRWIPY**	8.83E-12	**TNGGYGSITQ**	1.02E-19	**VPLICAGQTEDK**	5.68E-14	**RVNWARAGIDLK**	1.29E-10
Fusarium duplospermum	RSL66332.1	**TQGTLAMDPTSLIIPA**	3.17E-20	**NF**		**TNGGYGSVTQ**	1.34E-19	**VPLICAGQTEDK**	5.68E-14	**RVSWAGAGIDLK**	1.21E-12
Fusarium equiseti	CAG7554771.1	**TQGTLAMDPTSLIIPA**	3.17E-20	**LPNVRYVKWIPY**	1.46E-10	**TNGGYGSITQ**	1.02E-19	**VPLICAGQTEDK**	5.68E-14	**RVGWAGAGIDLK**	2.52E-11
Fusarium euwallaceae	RTE70560.1	**TQGTLAMDPTSLIIPA**	3.17E-20	**NF**		**TNGGYGSVTQ**	1.34E-19	**VPLICAGQTEDK**	5.68E-14	**RVSWAGAGIDLK**	1.21E-12
Fusarium falciforme	KAJ4142701.1	**TQGTLAMDPTSLIIPA**	3.17E-20	**NF**		**TNGGYGSVTQ**	1.34E-19	**VPLICAGQTEDK**	5.68E-14	**RVSWAGAGIDLK**	1.21E-12
Fusarium flagelliforme	XP_045981550.1	**TQGTLAMDPTSLIIPA**	3.17E-20	**LPNVRYVKWIPY**	1.46E-10	**TNGGYGSITQ**	1.02E-19	**VPLICAGQTEDK**	5.68E-14	**RVGWAGAGIDLK**	2.52E-11
Fusarium floridanum	RSL81449.1	**TQGTLAMDPTSLIIPA**	3.17E-20	**NF**		**TNGGYGSVTQ**	1.34E-19	**VPLICAGQTEDK**	5.68E-14	**RVSWAGAGIDLK**	1.21E-12
Fusarium fujikuroi	KLO93232.1	**TQGTLAMDPTSLIIPA**	3.17E-20	**PPNVRFIRWIPY**	8.83E-12	**TNGGYGSITQ**	1.02E-19	**VPLICAGQTEDK**	5.68E-14	**RVSWVRAGIDLK**	5.43E-10
Fusarium gaditjirri	KAF4947967.1	**TQGTLAMDPTSLIIPA**	3.17E-20	**PPYVRFIRWIPY**	1.50E-9	**TNGGYGSITQ**	1.02E-19	**VPLICAGQTEDK**	5.68E-14	**RVSWARAGIDLK**	1.98E-11
Fusarium graminearum	PCD36796.1	**TQGTLAMDPTSLIIPA**	3.17E-20	**LPNVRYVKWIPY**	1.46E-10	**TNGGYGSITQ**	1.02E-19	**VPLICAGQSEDK**	2.25E-13	**RVDWAGAGIDLK**	4.13E-12
Fusarium graminum	KAF4992880.1	**TQGTLAMDPTSLIIPA**	3.17E-20	**PLNVRFVKWIPY**	3.64E-10	**TNGGYGSITQ**	1.02E-19	**VPLICAGQTEDK**	5.68E-14	**RVSWARAGIDLK**	1.98E-11
Fusarium heterosporum	KAF5665361.1	**TQGTLAMDPTSLIIPA**	3.17E-20	**PTNVRFVKWIPY**	6.12E-11	**TNGGYGSITQ**	1.02E-19	**VPLICAGQTEDK**	5.68E-14	**RVSWARAGIDLK**	1.98E-11

Fusarium irregulare	KAJ4002951.1	**TQGTIAREASILIIPT**	6.60E-15	**PSNTRVIDYLPY**	1.62E-14	**MNAGYGGFLH**	6.74E-22	**VPMVLAGESEDK**	8.03E-14	**RGEWSGVAVNLR**	4.96E-13
Fusarium keratoplasticum	XP_052910685.1	**TQGTIARDYTDLLIPT**	3.22E-19	**PANTRVIDYLPY**	5.10E-14	**MNAGYGGFLH**	5.77E-20	**VPLVLAGETEDK**	3.69E-13	**RGQWSGVAVNLR**	1.21E-12
Fusarium kuroshium	RMJ08909.1	**TQGTIARDYTDLLIPT**	3.22E-19	**PANTRVIDYLPY**	5.10E-14	**MNAGYGGFLH**	5.77E-20	**VPLVLAGETEDK**	3.69E-13	**RGQWSGVAVNLR**	1.21E-12
Fusarium langsethiae	KPA39552.1	**TQGTIAQDASNLIIPT**	8.90E-17	**PSNTRIIDYLPY**	3.38E-13	**MNAGYGGFLH**	5.77E-20	**VPLVLAGESEDK**	1.14E-12	**RGDWSGVAVNLR**	8.16E-12
Fusarium longipes	RGP64175.1	**TQGTLAMDPTSLIIPA**	3.17E-20	**LPNVRYVKWIPY**	1.46E-10	**TNGGYGSITQ**	1.02E-19	**VPLICAGQSEDK**	2.25E-13	**RVDWAGAGIDLK**	4.13E-12
Fusarium mangiferae	XP_041689374.1	**TQGTLAMDPTSLIIPA**	3.17E-20	**PPNVRFIRWIPY**	8.83E-12	**TNGGYGSITQ**	1.02E-19	**VPLICAGQTEDK**	5.68E-14	**RVSWARAGIDLK**	1.98E-11
Fusarium mundagurra	KAF5715773.1	**TQGTLAMDPTSLIIPA**	3.17E-20	**PPNVRFIRWIPY**	8.83E-12	**TNGGYGSITQ**	1.02E-19	**VPLICAGQTEDK**	5.68E-14	**RVSWARAGIDLK**	1.98E-11
Fusarium musae	XP_044678177.1	**TQGTLAMDPTSLIIPA**	3.17E-20	**PPNVRFIRWIPY**	8.83E-12	**TNGGYGSITQ**	1.02E-19	**VPLICAGQTEDK**	5.68E-14	**RVSWARAGIDLQ**	1.58E-10
Fusarium napiforme	KAF5530306.1	**TQGTLAMDPTSLIIPA**	3.17E-20	**PPNVRFIRWIPY**	8.83E-12	**TNGGYGSITQ**	1.02E-19	**VPLICAGQTEDK**	5.68E-14	**RVSWARAGIDLK**	1.98E-11
Fusarium odoratissimum	XP_031068097.1	**TQGTIARDATNLIIPT**	1.42E-17	**PSNTRVIDYLPY**	1.62E-14	**MNAGYGGFLH**	5.77E-20	**VPLVLAGETEDK**	3.69E-13	**RGEWSGVAINLK**	9.86E-14
Fusarium oligoseptatum	RSM09688.1	**TQGTIARDYTDLLIPT**	3.22E-19	**PANTRVIDYLPY**	5.10E-14	**MNAGYGGFLH**	5.77E-20	**VPLVLAGETEDK**	3.69E-13	**RGQWSGVAVNLR**	1.21E-12
Fusarium oxysporum	KAJ4047383.1	**TQGTLAMDPTSLIIPA**	3.17E-20	**PPNVRFIRWIPY**	8.83E-12	**TNGGYGSITQ**	1.02E-19	**VPLICAGQTEDK**	5.68E-14	**RVSWASAGIDLK**	1.93E-10
Fusarium piperis	KAJ4328312.1	**TQGTLAMDPTSLIIPA**	3.17E-20	**LPNVRFAKWIPY**	1.18E-10	**TNGGYGSVTQ**	1.34E-19	**VPLICAGQTEDK**	5.68E-14	**RVSWAGAGIDLK**	1.21E-12
Fusarium poae	OBS22433.1	**TQGTLAMDPTSLIIPA**	3.17E-20	**NF**		**TNGGYGSITQ**	1.02E-19	**VPLICAGQSEDK**	2.25E-13	**RVDWAGAGIDLK**	4.13E-12
Fusarium proliferatum	KAG4256852.1	**TQGTLAMDPTSLIIPA**	3.17E-20	**PPNVRFIRWIPY**	8.83E-12	**TNGGYGSITQ**	1.02E-19	**VPLICAGQTEDK**	5.68E-14	**RVSWARAGIDLK**	1.98E-11
Fusarium pseudoanthophilum	KAF5585516.1	**TQGTLAMDPTSLIIPA**	3.17E-20	**PPNVRFIRWIPY**	8.83E-12	**TNGGYGSITQ**	1.02E-19	**VPLICAGQTEDK**	5.68E-14	**RVSWARAGIDLK**	1.98E-11
Fusarium pseudocircinatum	KAF5606259.1	**TQGTLAMDPTSLIIPA**	3.17E-20	**PANVRFIRWIPY**	1.01E-11	**TNGGYGSITQ**	1.02E-19	**VPLICAGQTEDK**	5.68E-14	**RVSWARAGIDLK**	1.98E-11
Fusarium pseudograminearum	QPC79958.1	**TQGTLAMDPTSLIIPA**	3.17E-20	**LPNVRYVKWIPY**	1.46E-10	**TNGGYGSITQ**	1.02E-19	**VPLICAGQSEDK**	2.25E-13	**RVDWAGAGIDLK**	4.13E-12
Fusarium redolens	XP_046045510.1	**TQGTLAMDPTSLIIPA**	3.17E-20	**PPNVRFIRWIPY**	8.83E-12	**TNGGYGSITQ**	1.02E-19	**VPLICAGQTEDK**	5.68E-14	**RVSWARAGIDLK**	1.98E-11
Fusarium sarcochroum	KAF4970128.1	**TQGTLAMDPTSLIIPA**	3.17E-20	**LPNVRFVKWIPY**	1.31E-10	**TNGGYGSVTQ**	1.34E-19	**VPLICAGQTEDK**	5.68E-14	**RVRWAGAGIDLR**	1.37E-11
Fusarium solani	XP_046129193.1	**TQGTLAMDPTSLIIPA**	3.17E-20	**NF**		**TNGGYGSVTQ**	1.34E-19	**VPLICAGQTEDK**	5.68E-14	**RVSWAGAGIDLK**	1.21E-12
Fusarium solani-melongenae	UPL00732.1	**TQGTLAMDPTSLIIPA**	3.17E-20	**NF**		**TNGGYGSVTQ**	1.34E-19	**VPLICAGQTEDK**	5.68E-14	**RVSWAGAGIDLK**	1.21E-12
Fusarium sporotrichioides	RGP62770.1	**TQGTLAMDPTSLIIPA**	3.17E-20	**LPNVRYVKWIPY**	1.46E-10	**TNGGYGSITQ**	1.02E-19	**VPLICAGQSEDK**	2.25E-13	**RVDWAGAGIDLK**	4.13E-12
Fusarium tjaetaba	XP_037210905.1	**TQGTLAMDPTSLIIPA**	3.17E-20	**PPNVRFIRWIPY**	8.83E-12	**TNGGYGSITQ**	1.02E-19	**VPLICAGQTEDK**	5.68E-14	**RVSWARAGIDLK**	1.98E-11
Fusarium tricinctum	KAH7263618.1	**TQGTLAMDPTSLIIPA**	3.17E-20	**PSNVRFIKWIPY**	2.56E-12	**TNGGYGSITQ**	1.02E-19	**VPLICAGQTEDK**	5.68E-14	**RVSWTRAGIDLK**	6.93E-11
Fusarium vanettenii	XP_003048134.1	**TQGTLAMDPTSLIIPA**	3.17E-20	**NF**		**TNGGYGSVTQ**	1.34E-19	**VPLICAGQTEDK**	5.68E-14	**RVSWAGAGIDLK**	1.21E-12
Fusarium venenatum	KAG8361315.1	**TQGTLAMDPTSLIIPA**	3.17E-20	**LPNVRYVKWIPY**	1.46E-10	**TNGGYGSITQ**	1.02E-19	**VPLICAGQSEDK**	2.25E-13	**RVDWAGAGIDLK**	4.13E-12

Fusarium verticillioides	XP_018759284.1	**TQGTLAMDPTSLIIPA**	3.17E-20	**PPNVRFIRWIPY**	8.83E-12	**TNGGYGSITQ**	1.02E-19	**VPLICAGQTEDK**	5.68E-14	**RVSWARAGIDLQ**	1.58E-10
Fusarium xylarioides	KAG5750058.1	**TQGTLAMDPTSLIIPA**	3.17E-20	**PPNVRFIRWIPY**	8.83E-12	**TNGGYGSITQ**	1.02E-19	**VPLICAGQTEDK**	5.68E-14	**RVSWAIAGVDLK**	3.76E-10
Fusarium zealandicum	KAF4976478.1	**TQGTLAMDPTSLIIPA**	3.17E-20	**NF**		**TNGGYGSITQ**	1.02E-19	**VPLICAGQTEDK**	5.68E-14	**RVSWAGAGVDLK**	2.31E-12
Dactylonectria estremocensis	KAH7159663.1	**TQGTLAMDPTSLIVPA**	7.19E-19	**FENVRFAKWIPY**	6.50E-10	**TNGGYGSITQ**	1.02E-19	**VPLICAGQTEDK**	5.68E-14	**RIHWAGAGIDLK**	4.48E-11
Dactylonectria macrodidyma	KAH7143671.1	**TQGTLAMDPTSLIVPA**	7.19E-19	**FDNVRFAKWIPY**	4.01E-10	**TNGGYGSITQ**	1.53E-17	**LPLICAGQTEDK**	2.58E-11	**RIHWAGAGIDLK**	4.48E-11
Ilyonectria destructans	KAH7002281.1	**TQGTLAMDPTSLIVPA**	7.19E-19	**FPNVRFARWIPY**	3.30E-10	**TNGGYGSITQ**	1.02E-19	**VPLICAGQTEDK**	5.68E-14	**RVNWAGAGIDLK**	1.37E-11
Ilyonectria robusta	XP_046110074.1	**TQGTVANDYSDLLIPT**	1.79E-18	**PANTRVIDYLPY**	5.10E-14	**MNAGYGGFLH**	4.54E-21	**VPMVLGGESEDK**	3.69E-13	**RGAWSGVAVNLR**	2.67E-12
Mariannaea sp. PMI_226	KAI5458596.1	**TQGTLAMDPTSLIIPA**	3.17E-20	**LPNVRFATFLPY**	1.04E-9	**TNGGYGSVTQ**	3.33E-18	**VPLICAGQTEDK**	5.68E-14	**RVEWAGAGIDLR**	3.28E-13
Stylonectria norvegica	KAF7554329.1	**TQGTVANDYADLLIPA**	4.21E-16	**PANTRVIDYLPY**	5.10E-14	**MNAGYGGFLH**	6.43E-21	**VPMVLAGETEDK**	1.75E-14	**RGEWSGVAVNLR**	4.96E-13
Neonectria ditissima	KPM40292.1	**TQGTLAMDPTSLIMPA**	1.54E-18	**LPNVRFAKWIPY**	1.18E-10	**TNGGYGSITQ**	1.04E-18	**IPLICAGQTEDK**	1.14E-12	**RVAWAGAGIDLK**	1.21E-12
Claviceps africana	KAG5919883.1	**TQGTVAVNHRDLLIPT**	1.42E-17	**PSNAHVIDYLPY**	1.86E-13	**SNGGYGGFIQ**	2.43E-16	**VPMILAGTTEDK**	2.69E-13	**RGEYAGIAINLR**	4.74E-12
Claviceps arundinis	KAG5966788.1	**TQGTVAVEYRHLLIPA**	3.40E-16	**PPNTHVIDYLPY**	6.04E-13	**MNAGYGGVIQ**	6.35E-19	**VPMVLAGGTEDK**	1.46E-13	**RAEHAGIAVNLR**	7.72E-11
Claviceps capensis	KAG5921632.1	**TQGTVAVEYRHLLIPT**	7.92E-17	**PPNTHVIDYLPY**	6.04E-13	**MNAGYGGVIQ**	6.35E-19	**VPMVLAGGTEDK**	1.46E-13	**RAEHAGIAFNLR**	4.96E-10
Claviceps cyperi	KAG5965626.1	**TQGTVAVEYRHLLIPT**	7.92E-17	**PPNTHVIDYLAY**	4.88E-11	**MNAGYGGVIQ**	6.35E-19	**VPMVLAGGTEDK**	1.46E-13	**RAEYAGIAANLR**	4.12E-10
Claviceps digitariae	KAG5982393.1	**TQGTVAMEYHELLVPT**	7.10E-16	**PSNAHVIDYLPY**	1.86E-13	**SNAGYGGFIQ**	1.04E-18	**VPMVLGGETEDK**	1.01E-13	**RGEYSGIAINLR**	7.14E-12
Claviceps humidiphila	KAG6118839.1	**TQGTVAVEYRHLLIPA**	3.40E-16	**PPNTHVIDYLPY**	6.04E-13	**MNAGYGGVIQ**	6.35E-19	**VPMVLAGGTEDK**	1.46E-13	**RAEHAGIAVNLR**	7.72E-11
Claviceps lovelessii	KAG5986980.1	**TQGTVAREYEDLIIPT**	1.33E-18	**PPNAHVIDYLPY**	2.75E-13	**SNAGYGGFLQ**	4.72E-17	**VPMVLAGETEDK**	1.75E-14	**RGEYAGVAINLR**	3.58E-12
Claviceps maximensis	KAG6002038.1	**TQGTVAMDYENLIIPT**	2.00E-21	**PPNARVIDYLSY**	6.82E-14	**SNAGNGGLIQ**	3.71E-15	**VPMVFAGITEDK**	4.13E-12	**RGEYAGIAINLR**	4.74E-12
Claviceps monticola	KAG5944898.1	**TQGTVAVEYRHLIIPT**	1.24E-17	**PPNTHVIDYLPY**	6.04E-13	**MNAGYGGVIQ**	6.35E-19	**VPMVLAGGTEDK**	1.46E-13	**RAEHAGIAFNLR**	4.96E-10
Claviceps purpurea	KAG6132685.1	**TQGTVAVEYRHLIIPT**	1.24E-17	**PPNTHVIDYLPY**	6.04E-13	**MNAGYGGVIQ**	6.35E-19	**VPMVLAGGTEDK**	1.46E-13	**RAEHAGIAFNLR**	4.96E-10
Claviceps pusilla	KAG5989301.1	**TQGTVATEYKDLIIPT**	3.22E-19	**PPNAHVIDYLPY**	2.75E-13	**SNAGYGGFLQ**	4.72E-17	**VPMVLAGETEDK**	1.75E-14	**RGEYAGVAINLR**	3.58E-12
Claviceps sorghi	KAG5949601.1	**TQGTVAVKYQDLLIPT**	1.12E-16	**PPNAHVIDYLPY**	2.75E-13	**SNAGYGGFIQ**	2.97E-19	**VPMVLAGTTEDK**	6.91E-14	**RGAYSGIAINLK**	1.75E-11
Claviceps spartinae	KAG5994791.1	**TQGTVAVEYRHLLIPA**	3.40E-16	**PPNTHVIDYLPY**	6.04E-13	**MNAGYGGVIQ**	6.35E-19	**VPMVLAGGTEDK**	1.46E-13	**RAEHAGIAVNLR**	7.72E-11
Metarhizium acridum	KAG8422976.1	**NF**		**PPNARVIDYLPY**	4.76E-15	**MNAGYGGFIQ**	7.68E-20	**VPMILAGDSEDK**	4.95E-13	**RGEYAGVAVNLK**	4.13E-12
Metarhizium album	XP_040683175.1	**TQGTLATDPTSLIIPS**	4.47E-19	**NF**		**TNGGYGSIKQ**	1.67E-18	**VPLLCAGQSEDK**	1.14E-12	**RVAYSRLGIDLK**	7.76E-10
Metarhizium anisopliae	KJK80520.1	**SQGTIAVDYAHLLIPA**	1.12E-16	**PPNARVVDYLPY**	2.50E-14	**MNAGYGGFIQ**	2.97E-19	**VPMVLAGGSEDK**	4.95E-13	**RGEYSGVAVNLR**	9.34E-12
Metarhizium brunneum	XP_014548452.1	**SQGTIAVDYADLLIPA**	1.24E-17	**PPNARVIDYLPY**	4.76E-15	**MNAGYGGFIQ**	2.97E-19	**VPMVLAGGSEDK**	4.95E-13	**RGEYSGVAVNLR**	9.34E-12

Metarhizium guizhouense	KID92007.1	SQGTIAVDYADLLIPA	1.24E-17	PPNARVIDYLPY	4.76E-15	MNAGYGGFIQ	5.77E-20	VPMVLAGGSEDK	4.95E-13	RGEYSGVAVNLR	9.34E-12
Metarhizium humberi	KAH0597174.1	SQGTIAVDYADLLIPA	2.67E-17	PPNARVVDYLPY	2.50E-14	MNAGYGGFIQ	2.97E-19	VPMVLAGGSEDK	4.95E-13	RGEYSGVAVNLR	9.34E-12
Metarhizium rileyi	OAA43280.1	SQGTVSTNWDELIVPT	7.92E-17	PANARVADYLPY	2.50E-14	SNAGYGAFGH	1.24E-17	VPAIFAGETEEK	4.89E-10	RAEWAGFAYNLR	6.23E-11
Metarhizium robertsii	XP_007822448.1	SQGTIAVDYADLLIPA	2.67E-17	PPNARVIDYLPY	4.76E-15	MNAGYGGFIQ	2.97E-19	VPMVLAGGSEDK	4.95E-13	RGEYSGVAVNLR	9.34E-12
Ustilaginoidea virens	XP_042995238.1	TQGTFAVDYAHLLMPA	9.65E-16	PPNARVADFLPY	1.19E-13	TNAGYGGFIH	1.06E-20	VPMVLAGDTEDK	3.11E-14	RGEYAGIAVNLR	8.16E-12
Conoideocrella luteorostrata	KAK2616729.1	TQGTVAMNYTHLIIPT	2.53E-21	PPNAHVIDYLPY	2.75E-13	MNAGYGGFIQ	5.77E-20	VPMVLAGSTEDK	7.60E-13	RGEYSGVAINLR	5.45E-12
Moelleriella libera	KZZ90826.1	TQGTVAVDYSQLVIPA	3.19E-18	APNAHVADYLSY	6.12E-11	MNAGYGGFVH	1.32E-18	VPMVLAGASEEK	2.83E-11	RGEYAGIGINLR	1.97E-12
Epichloe festucae	QPH11511.1	CQGTVAMDFNQLVIPT	3.22E-19	PSNCRVVDYIPY	4.15E-13	TTGGYGSLQR	2.06E-15	TPLVMAGTTEEK	2.90E-10	RAEWAGVAVNLR	8.67E-13
Trichoderma arundinaceum	RFU78995.1	TQGTLAMDPTSLIMPA	1.54E-18	IANIRYADWLPY	5.47E-9	TNGGYGSITQ	2.02E-20	VPLLCAGQTEDK	3.69E-13	RVRWCGVGIDLK	3.58E-12
Trichoderma asperelloides	KAH8127233.1	TQGTLAMDPTSLIIPS	9.69E-20	AVNVCYADWLPY	3.91E-9	TNGGYGSITQ	2.75E-20	VPLLCAGQTEDK	3.69E-13	RVAWCGAGIDLK	8.67E-13
Trichoderma asperellum	UKZ86906.1	TQGTLAMDPTSLIIPS	9.69E-20	AVNVCYADWLPY	3.91E-9	TNGGYGSITQ	2.75E-20	VPLLCAGQTEDK	3.69E-13	RVTWCGAGIDLK	3.08E-12
Trichoderma atroviride	UKZ67550.1	TQGTLAMDPTSLITPS	4.37E-17	TDNVRYADWLPY	2.98E-10	TNGGYGSITQ	2.75E-20	VPLLCAGQTEDK	3.69E-13	RVAWCGAGIDLK	8.67E-13
Trichoderma breve	XP_056030790.1	TQGTLATDPTSLIIPS	4.47E-19	SANVRYADWLPY	4.35E-11	TNGGYGSITQ	2.75E-20	VPLLCAGQSEDK	1.14E-12	RVTWCGAGIDLR	4.74E-12
Trichoderma citrinoviride	XP_024752346.1	TQGTLAMNPTSLIIPS	1.16E-19	ASNVRYADWLPY	3.44E-11	TNGGYGSITQ	2.75E-20	VPLLCAGQTEDK	3.69E-13	RITWCGAGIDLK	1.37E-11
Trichoderma cornu-damae	KAH6605774.1	TQGTLAMDPISLIIPS	3.42E-17	AANVRYANWLPY	7.85E-10	TNGGYGSITQ	2.75E-20	VPLLCAGQTEDK	3.69E-13	RVAWCGAGIDLK	8.67E-13
Trichoderma gracile	KAH0492740.1	TQGTLAMNPTSLIIPS	1.16E-19	ASNVRYADWLPY	3.44E-11	TNGGYGSITQ	1.32E-18	VPLLCAGQTEDK	3.69E-13	RITWCGVGIDLK	7.14E-12
Trichoderma guizhouense	OPB36448.1	TQGTLATDPTSLIIPS	4.47E-19	SANVRYADWLPY	4.35E-11	TNGGYGSITQ	1.02E-19	VPLLCAGQSEDK	1.14E-12	RVTWCGAGIDLR	4.74E-12
Trichoderma harzianum	KKO97596.1	TQGTLATDPTSLIIPS	4.47E-19	SVNVRYANWLPY	4.25E-9	TNGGYGSITQ	2.75E-20	VPLLCAGQSEDK	1.14E-12	RVTWCGAGIDLR	4.74E-12
Trichoderma longibrachiatum	PTB77621.1	TQGTLAMNPTSLIIPS	1.16E-19	ASNVRYADWLPY	3.44E-11	TNGGYGSITQ	2.75E-20	VPLLCAGQTEDK	3.69E-13	RVSWCGAGIDLR	1.44E-12
Trichoderma reesei	XP_006968899.1	TQGTLAMNPTSLIIPS	1.16E-19	APNVRYADWLPY	4.35E-11	TNGGYGSITQ	2.75E-20	VPLLCAGQTEDK	3.69E-13	RITWCGAGIDLK	1.37E-11
Trichoderma semiorbis	KAH0529301.1	TQGTLATDPTSLIIPS	4.47E-19	SANVRYAGWLPY	1.14E-9	TNGGYGSITQ	2.75E-20	VPLLCAGQSEDK	1.14E-12	RVTWCGAGIDLR	4.74E-12
Trichoderma simmonsii	QYS95815.1	TQGTLATDPTSLIIPS	4.47E-19	SANVRYADWLPY	4.35E-11	TNGGYGSITQ	1.02E-19	VPLLCAGQSEDK	1.14E-12	RVTWCGAGIDLR	4.74E-12
Trichoderma virens	XP_013957984.1	TQGTLAMNPTSLIIPS	1.16E-19	ADNVRYADWLPY	1.18E-10	TNGGYGSITQ	2.75E-20	VPLVCAGQSEDK	5.68E-14	RVTWCGAGIDLR	4.74E-12
Escovopsis weberi	KOS17718.1	NF		NF		TNAGYQPMLQ	9.77E-13	NF		NF	
Cordyceps fumosorosea	XP_018701796.1	SQGTVAVHYDQLLVPT	3.78E-16	PSNCHVVDYLSY	5.89E-12	VNAGYGGFMH	2.97E-19	VPMVLAGASEDK	2.49E-12	RAEFAGVGINLR	1.55E-11
Cordyceps javanica	TQV95450.1	SQGTVAMRYDHLLIPT	1.09E-17	PSNAHVVDYLSY	1.01E-12	LNAGYGGFIH	7.19E-19	IPMVLAGGSEDK	5.75E-12	RGEFAGVGVNLR	1.20E-11
Cordyceps militaris	XP_006674754.1	SQGTVAQRYDQLLIPA	1.30E-15	PSNAHVVDYLSY	1.01E-12	MNAGYGGFMH	6.74E-22	VPMVLAGGTEDK	1.46E-13	RGEFAGVGINLR	7.14E-12

Akanthomyces lecanii	OAA70212.1	SQGTVAVRYDQLLIPA	2.45E-16	PSNAHVVDYLSY	1.01E-12	LNAGYGGFIH	6.66E-20	VPMVLAGGSEDK	4.95E-13	RGEFAGVGINLR	7.14E-12
Akanthomyces muscarius	XP_056054863.1	SQGTVAVRYDQLLIPA	2.45E-16	PSNTHVVDYLSY	1.92E-12	LNAGYGGFIH	6.66E-20	VPMVLAGGSEDK	4.95E-13	RGEFAGVGINLR	7.14E-12
Beauveria bassiana	XP_008602005.1	SQGTVAVRYDQLLVPA	1.59E-15	PSNAYTVDYLSY	6.84E-11	LNAGYGGFMH	1.72E-20	VPMVLAGGSEDK	4.95E-13	RGEFAGVGINLR	7.14E-12
Beauveria brongniartii	OAA39919.1	SQGTVAVRYDQLVVPA	1.44E-15	PSNAYTVDYLSY	6.84E-11	LNAGYGGFMH	1.72E-20	VPMVLAGGSEDK	4.95E-13	RGEFAGVGINLR	7.14E-12
Lecanicillium saksenae	KAJ3497866.1	SQGTVAVDYSQLLIPT	9.80E-19	PSNAHVIDYLSY	6.04E-13	VNAGYGGFMH	5.61E-19	VPMVLAGGSEDK	4.95E-13	RGEFAGVAINLR	1.55E-11
Niveomyces insectorum	OAA57048.1	AQGTIATSYDELILPT	3.40E-16	PSNVRVLDYFPY	6.73E-12	TNGGYGSCTH	2.97E-19	VPMVVGGITEDK	3.24E-12	RAEYAGLAVNLR	1.17E-10
Purpureocillium lavendulum	KAJ6441125.1	TQGTLAMDPTSLIVPA	1.14E-18	PSNVHFATWMPY	4.01E-10	TNGGYGSITQ	1.02E-19	VPLLCAGQSEDK	1.14E-12	RVVWTGVGVDLK	1.75E-11
Purpureocillium lilacinum	XP_018181559.1	TQGTLAMDPTSLILPT	2.15E-20	PSNVRYATWMPY	1.31E-10	TNGGYGSITQ	1.02E-19	VPLLCAGQSEDK	1.14E-12	RVVWAGVGIDLK	2.31E-12
Purpureocillium takamizusanense	XP_047841106.1	TQGTLAMDPTSLILPA	2.31E-19	PDNVRFATWMPY	3.64E-10	TNGGYGSITQ	1.02E-19	VPLLCAGQSEDK	1.14E-12	RVVWAGVGIDLK	2.31E-12
Hirsutella minnesotensis	KJZ71824.1	CQGTVNLDWRELVIPT	8.90E-17	PSNARVIDYLPY	9.29E-16	SNGGYGGLQH	5.53E-16	NF		RGAWAGFAVNLK	2.24E-11
Hirsutella rhossiliensis	XP_044720357.1	SQGTVNKNWNELVLPT	3.13E-15	PANARVIDYLPY	9.57E-15	SNAGYGAFRH	1.46E-15	VATVFAGETEDK	1.22E-10	RGEWAGFAHNLK	1.98E-11
Tolypocladium ophioglossoides	KND93039.1	TQGTLAMDPTSLIIPS	4.47E-19	PSNVRLAEWLPY	2.98E-10	TNGGYGSITQ	1.02E-19	VPLLCAGQSEDK	1.14E-12	RVVWAGVGIDLK	2.31E-12
Tolypocladium paradoxum	POR35566.1	TQGTLAMDPTSLIVPS	6.39E-18	PSNVRLAEWLPY	2.98E-10	TNGGYGSITQ	1.02E-19	VPLLCAGQSEDK	1.14E-12	RVVWAGVGIDLK	2.31E-12
Ophiocordyceps sinensis	EQK98407.1	SQGTVNMDWDELVLPT	8.90E-17	PANARVVDYLPY	3.84E-14	SNAGYGAFRH	2.99E-14	VATVFAGETEEK	5.12E-10	RGEWAGFALNLK	7.72E-11
Clonostachys byssicola	CAG9995431.1	TQGTVVLDYHNLVIPT	6.26E-17	PANARVVDYFPY	2.26E-13	MNAGYGGFIH	4.54E-21	VPMVLGGGTEDK	5.67E-13	RGEFAGVAVNLR	2.52E-11
Clonostachys chloroleuca	CAI6093753.1	TQGTVVLDYRNLVIPT	1.41E-16	PSNARVVDYFPY	1.19E-13	MNAGYGGFIH	4.54E-21	VPMVLGGGTEDK	5.67E-13	RGEFAGVAVNLR	2.52E-11
Clonostachys rhizophaga	CAH0019948.1	TQGTVVLDYRNLVIPT	1.41E-16	PSNARVVDYFPY	1.19E-13	MNAGYGGFIH	4.54E-21	VPMVLGGGTEDK	5.67E-13	RGEFAGVAVNLR	2.52E-11
Clonostachys rosea	CAG9952845.1	TQGTVALDYSNLVIPT	4.23E-18	PSNARVVDYFPY	1.19E-13	MNAGYGGFID	4.17E-18	VPMVLGGGTADK	1.72E-11	RGEYAGVAVNLR	6.27E-12
Clonostachys solani	CAH0038538.1	TQGTVVLDYSNLVIPT	7.92E-17	PSNARVVDYFPY	1.19E-13	MNAGYGGFIH	4.54E-21	VPMVLGGGTEDK	5.67E-13	RGEHAGVAVNLR	3.18E-11
Emericellopsis atlantica	XP_046121130.1	TQGTVALEYGHLIMPT	1.94E-15	PGNARVIDVLNY	4.43E-10	FNAGFGGLTH	4.21E-16	VPMVFAGDTEDK	5.67E-13	RGEWAGVGINLK	3.28E-15
Hapsidospora chrysogena	KFH48365.1	TQGTLAMNPTSLIVPS	2.07E-18	NF		TNGGYGSITQ	2.75E-20	VPLLCAGQTEDK	3.69E-13	RVSWAGAGIDLA	3.18E-11
Stachybotrys chartarum	KFA76153.1	TQGTLATDPTSLILPA	2.39E-18	PRNAHFATFLPY	7.15E-10	TNGGYGSITQ	1.34E-19	VPLLCAGQTEDK	3.69E-13	RVAWCGAGIDLK	8.67E-13
Stachybotrys chlorohalonata	KFA67805.1	TQGTLATDPTSLILPA	2.39E-18	PRNAHFATFLPY	7.15E-10	TNGGYGSITQ	1.34E-19	VPLLCAGQTEDK	3.69E-13	RVAWCGAGIDLK	8.67E-13
Stachybotrys elegans	KAH7322735.1	TQGTLAMDPNDLVIPA	4.47E-19	FNNLRFAKWLPY	7.61E-9	TNGGYGSITQ	1.34E-19	VPLLCAGQTEDK	3.69E-13	RVAWCQAGIDLK	7.72E-11
Trichothecium roseum	KAI9901541.1	TQGTVAVDYKELVVPT	2.20E-16	PDNTRVIDHLSY	6.84E-11	CNGGYGGFIH	1.17E-19	VPMIMGGESEDK	1.50E-12	RGEWAGIAVNLK	2.10E-13
Aphanocladium album	KAJ6789860.1	SQGTVAVDYSHLLIPT	1.33E-18	PSNAHVVDYLSY	1.01E-12	ANAGYGGFMH	1.53E-19	VPMVLAGGSEDK	4.95E-13	RGEFAGVAINLR	1.55E-11

Colletotrichum abscissum	KAI3530008.1	SQGTVNMDYSELVLPT	1.14E-18	PSNARVVDYMPY	2.75E-13	SNGGYGALTH	2.97E-18	VPIVLAGESEEK	1.01E-10	RAVYAGLGVSLS	2.70E-8
Colletotrichum acutatum	KAK1724125.1	TQGTYATNAANLITPT	1.04E-14	PSNARVANFIPH	1.18E-10	TNAGYNGALTH	9.01E-11	VPLVCAGRSEDK	1.50E-12	RVAWSGAGIDLA	4.48E-11
Colletotrichum aenigma	XP_037184972.1	CQGTVAMDLTQLVIPT	1.42E-17	PPNCRVTDYVPY	1.89E-11	TTGGYGAFQR	3.20E-16	NF		RAAWAGVGVDLR	4.74E-12
Colletotrichum asianum	KAF0316378.1	SQGTLNPLWSELIIPA	1.48E-14	PANARVIDYVPH	1.30E-11	SNAGYGTLTH	3.73E-18	VPVLLAGENEEK	8.67E-10	RAVYAGVGLSLG	8.97E-9
Colletotrichum camelliae	KAH0442418.1	AQGTIATDYTDLIIPT	6.19E-21	PANTRVADFLSY	1.01E-12	LNAGYGGFLH	8.84E-20	VPMVLGGDTEDK	1.65E-13	RGEWTGVAHNLK	4.74E-12
Colletotrichum caudatum	KAK2059483.1	TQGTVAMEYGNLVIPT	1.54E-18	PPNTRVADFLPY	2.75E-13	TNAGYGGFTH	2.27E-22	VPMVLGGDTEDK	1.65E-13	RGQWAGVAYNLK	2.31E-12
Colletotrichum cereale	KAK1986054.1	TQGTVAMEYTNLVIPT	6.74E-20	PANARVVDFVPY	2.22E-12	TNAGYGGFAH	1.17E-19	VPMVLGGDTEDK	1.65E-13	RGQWAGVAYNLK	2.31E-12
Colletotrichum chlorophyti	OLN96240.1	SQGTVTHDYSELVLPT	5.20E-16	PPNARVINYVPY	2.14E-11	SNGGYGALMH	3.49E-17	VPTILAGESQEK	3.63E-10	RAVYAGLGVSLA	1.63E-8
Colletotrichum chrysophilum	XP_053034241.1	NF		PANARIIDYLPY	2.75E-13	SNAGYGTLTH	3.73E-18	VPVLLAGENEEK	8.67E-10	RAVYAGVGLSLG	8.97E-9
Colletotrichum eremochloae	KAK2005828.1	SQGTVNSNYNDLVLPT	2.36E-17	PPNARVLDYMPY	8.83E-12	SNAGYGALTH	4.66E-18	VPVVLAGESQEK	9.28E-11	RAVYAGLGVSLA	1.63E-8
Colletotrichum falcatum	KAK1994865.1	SQGTVSSDHTELLLPT	1.44E-15	PPNVRVLDYLPY	3.42E-12	SNGGYGALTH	2.36E-18	VPVVLAGESQEK	9.28E-11	RAVYAGLGVSLG	7.70E-9
Colletotrichum filicis	KAI3528431.1	SQGTVNMDYSELVLPT	1.14E-18	PSNARFVDYMPY	1.19E-12	SNGGYGALTH	2.97E-18	VPIVLAGESEEK	1.01E-10	RAVYAGLGVSLS	2.70E-8
Colletotrichum fioriniae	KAJ0331578.1	AQGTIAINYTDLIIPT	2.15E-20	PANTKVIDFLSY	5.16E-12	LNAGYGGFLH	8.84E-20	VPMVLGGDTEDK	1.65E-13	RGQWTGVAVNLK	2.67E-12
Colletotrichum fructicola	XP_031878303.1	SQGTLNPLWSELIIPA	1.48E-14	PANARIIDYLPY	2.75E-13	SNAGYGTLTH	3.73E-18	VPVLLAGENEEK	8.67E-10	RAVYAGVGLSLG	8.97E-9
Colletotrichum gloeosporioides	KAH9225977.1	SQGTLNPLWSELIIPA	1.48E-14	PANARIIDYLPY	2.75E-13	SNAGYGTLTH	3.73E-18	VPVLLAGENEEK	8.67E-10	RAVHAGVGLSLG	2.34E-8
Colletotrichum godetiae	KAK1657259.1	TQGTYATNSSNLITPT	6.02E-15	PSNARVASFIPH	2.44E-10	TNAGYNGVLA	1.67E-16	VPMICAGRSEDK	4.21E-13	RVAWSGAGIDLA	4.48E-11
Colletotrichum graminicola	XP_008100482.1	TQGTYATNAANLISPT	1.13E-14	PENARVADFVPH	1.46E-10	TNAGYNGVLA	1.67E-16	VPMVCAGRTEDK	2.55E-14	RVAWSGAGIDLG	1.37E-11
Colletotrichum higginsianum	GJC91227.1	TQGTIALDYTNLVIPA	9.80E-19	PANARVVDFISY	7.19E-13	MNAGYGGFMH	2.65E-21	VPMVLGGDTEDK	1.65E-13	RGQWAGVAHNLK	1.97E-12
Colletotrichum incanum	OHW97533.1	TQGTIALDYTDLVIPT	3.85E-20	PANTRVVDFISY	1.41E-12	MNAGYGGFMH	2.65E-21	VPMVLGGDTEDK	1.65E-13	RGQWAGVAYNLK	2.31E-12
Colletotrichum karsti	XP_038743776.1	AQGTIATDYNDLVIPT	8.40E-19	PANTRVADFLSY	1.01E-12	MNAGYGGFLH	5.41E-21	VPMVLGGDTEDK	1.65E-13	RGEYVGVAYNLK	4.52E-10
Colletotrichum limetticola	KAK0371775.1	AQGTIAINYTDLIIPT	2.15E-20	PANTKVVDFLSY	7.71E-12	LNAGYGGFLH	8.84E-20	VPMVLGGDTEDK	1.65E-13	RGQWTGVAVNLK	2.67E-12
Colletotrichum liriopes	GJC77217.1	TQGTIALDYTDLVIPT	3.85E-20	PANTRVVDFISY	1.41E-12	MNAGYGGFMH	8.30E-22	VPMVLGGDTEDK	1.65E-13	RGQWAGVAYNLQ	2.83E-11
Colletotrichum lupini	KAK1705670.1	AQGTIAINYTDLIIPT	2.15E-20	PANTKVVDFLSY	7.71E-12	LNAGYGGFLH	8.84E-20	VPMVLGGDTEDK	1.65E-13	RGQWTGVAVNLK	2.67E-12
Colletotrichum musicola	KAF6838802.1	TQGTVATNYNDLIIPT	2.53E-21	PANTKVVDYFSY	8.83E-12	MNAGYGGFLH	5.41E-21	VPMVLGGDTEDK	1.65E-13	RAQWAGVAHNLK	4.74E-12
Colletotrichum navitas	KAK1573862.1	SQGTVASDYTELALPT	3.05E-16	PPNVRVLDYMPY	1.47E-11	SNGGYGALTH	2.36E-18	VPVVLAGESQEK	9.28E-11	RAVYAGIGVSLA	4.47E-9
Colletotrichum noveboracense	KAJ0277668.1	AQGTIATDYTDLIIPT	6.19E-21	PANTRVADFLSY	1.01E-12	LNAGYGGFLH	8.84E-20	VPMVLGGDTEDK	1.65E-13	RGEWTGVAHNLK	4.74E-12
Colletotrichum nupharicola	KAJ0337848.1	AQGTIATDYTDLIIPT	6.19E-21	PANTRVADFLSY	1.01E-12	LNAGYGGFLH	8.84E-20	VPMVLGGDTEDK	1.65E-13	RGEWTGVAHNLK	4.74E-12

Colletotrichum nymphaeae	KXH45981.1	AQGTIAINYTDLIIPT	2.15E-20	PANTKVIDFLSY	5.16E-12	LNAGYGGFLH	8.84E-20	VPMVLGGDTEDK	1.65E-13	RGQWTGVAVNLK	2.67E-12
Colletotrichum orbiculare	TDZ14673.1	GQGTVAMDYTDLVIPA	9.56E-18	PANTRVVDYLHY	2.96E-12	MNAGYGGFIH	2.65E-21	VPMVLGGDSEDK	5.67E-13	RGEWAGVAHNLK	8.67E-13
Colletotrichum orchidophilum	XP_022475743.1	AQGTIATDYTDLIIPT	6.19E-21	PANTKVIDFLSY	5.16E-12	LNAGYGGYLH	1.48E-18	VPMVLGGDTEDK	1.65E-13	RGQWSGVAYNLK	3.58E-12
Colletotrichum paranaense	KAK1540822.1	AQGTIAINYTDLIIPT	2.15E-20	PANTKVVDFLSY	7.71E-12	LNAGYGGFLH	8.84E-20	VPMVLGGDTEDK	1.65E-13	RGQWTGVAVNLK	2.67E-12
Colletotrichum phormii	KAK1635646.1	TQGTIATGYTDLIIPT	2.07E-18	PANTKVIDFLSY	5.16E-12	MNAGYGGFLH	7.63E-21	VPMVLGGDTEDK	1.65E-13	RGQWAGIAFNLK	1.06E-11
Colletotrichum plurivorum	KAF6829629.1	TQGTVATNYNDLIIPT	2.53E-21	PANTKVVDYFSY	8.83E-12	MNAGYGGFLH	5.41E-21	VPMVLGGDTEDK	1.65E-13	RAQWAGVAHNLK	4.74E-12
Colletotrichum salicis	KXH39943.1	AQGTIATDYTDLIIPT	6.19E-21	PANTKVVDFLSY	7.71E-12	LNAGYGGFLH	1.17E-19	VPMVLGGDTEDK	1.65E-13	RGQWTGIAFNLK	4.00E-11
Colletotrichum scovillei	XP_035327966.1	AQGTIAIDYTDLIIPT	1.76E-20	PANTKVIDFLSY	5.16E-12	LNAGYGGFLH	8.84E-20	VPMVLGGDTEDK	1.65E-13	RGQWTGVAVNLK	2.67E-12
Colletotrichum shisoi	TQN67810.1	TQGTIALDYTNLVIPA	9.80E-19	PANARVVDFISY	7.19E-13	MNAGYGGFMH	2.65E-21	VPMVLGGDTEDK	1.65E-13	RGQWAGVAHNLK	1.97E-12
Colletotrichum siamense	KAF4814982.1	AQGTVATDYTDLIIPT	1.57E-21	PANTRVADFLSY	1.01E-12	LNAGYGGFLH	8.84E-20	VPMVLGGDTEDK	1.65E-13	RGEWTGVAHNLK	4.74E-12
Colletotrichum sidae	TEA21432.1	GQGTVAMDYTDLVIPA	9.56E-18	PANTRVVDYLHY	2.96E-12	MNAGYGGFIH	2.65E-21	VPMVLGGDSEDK	5.67E-13	RGEWAGVAHNLK	8.67E-13
Colletotrichum simmondsii	KXH40793.1	SQGTVNMDYSELVLPT	1.14E-18	PSNARVVDYMPY	2.75E-13	SNGGYGALTH	2.97E-18	VPIVLAGESEEK	1.01E-10	RAVYAGLGVSLS	2.70E-8
Colletotrichum sojae	KAF6806549.1	TQGTVATNYNDLIIPT	2.53E-21	PANTKVVDYFSY	8.83E-12	MNAGYGGFLH	5.41E-21	VPMVLGGDTEDK	1.65E-13	RAQWAGVAHNLK	4.74E-12
Colletotrichum somersetense	KAK2043394.1	TQGTVVMEYGNLVIPT	3.87E-17	PQNTRVVDFLPY	3.93E-12	TNAGYGGFTH	2.27E-22	VPMVLGGDTEDK	1.65E-13	RGQWAGVAYNLK	2.31E-12
Colletotrichum sublineola	KDN67295.1	AQGTEVLDHRELIIPT	4.16E-15	PSNARVVDYFPY	1.19E-13	SNSGYGGFQH	7.68E-20	VPMVQAGNVFDK	3.09E-9	RIEWSGLGIYMV	7.11E-8
Colletotrichum tamarilloi	KAK1490332.1	SQGTVNMDYSELVLPT	1.14E-18	PSNARVVDYMPY	2.75E-13	SNGGYGALTH	2.97E-18	VPIVLAGESEEK	1.01E-10	RAVYAGLGVSLS	2.70E-8
Colletotrichum tanaceti	KAJ0162061.1	AQGTEVLDHRELIIPT	3.78E-15	PANARVVDYFPY	2.26E-13	SNSGYGGFQH	5.61E-19	**NF**		RIEWCGLGVYLE	3.52E-9
Colletotrichum tofieldiae	KZL66573.1	TQGTIALDYTDLVIPT	3.85E-20	PANTRVVDFISY	1.41E-12	MNAGYGGFMH	8.30E-22	VPMVLGGDTEDK	1.65E-13	RGQWAGVAYNLQ	2.83E-11
Colletotrichum trifolii	TDZ54034.1	SQGTLNPNYTDLILPS	4.93E-17	PSNARVVDYIPY	1.62E-14	SNAGYGTFTH	2.61E-19	VPVVLAGENEEK	2.12E-10	RAIYAGIGLSLE	1.06E-17
Colletotrichum tropicale	KAJ3960792.1	AQGTVATDYTDLIIPT	1.57E-21	PANTRVADFLSY	1.01E-12	LNAGYGGFLH	8.84E-20	VPMVLGGDTEDK	1.65E-13	RGEWTGVAHNLK	4.74E-12
Colletotrichum truncatum	XP_036576291.1	TQGTVAVDYKDLIIPT	1.17E-20	PANARIIDYLPY	2.75E-13	LNAGYGGFLH	6.66E-20	VPMVLGGDTEDK	1.65E-13	RGEWAGVAYNLR	1.68E-12
Colletotrichum viniferum	KAF4925869.1	AQGTVATDYTDLIIPT	1.57E-21	PANTRVADFLSY	1.01E-12	LNAGYGGFLH	8.84E-20	VPMVLGGDTEDK	1.65E-13	RGEWTGVAHNLK	4.74E-12
Colletotrichum zoysiae	KAK2035655.1	TQGTIVMEYGNLVIPT	7.92E-17	PQNTRVVDFLPY	3.93E-12	TNAGYGGFTH	2.27E-22	VPMVLGGDTEDK	1.65E-13	RGQWAGVAYNLK	2.31E-12
Sodiomyces alkalinus	XP_028465600.1	SQGTVATEYVNLLIPA	2.13E-15	PANTRVVDYFSY	1.41E-12	MNAGWGGFQQ	1.32E-18	VPMVLAGNTEDK	8.58E-13	RAAYSGVAIDLK	6.23E-11
Daldinia bambusicola	KAI1805383.1	AQGTVEINPRDLIVPT	8.90E-17	PPNARIADYLSY	1.01E-12	HNAGFGAVNH	3.38E-19	VPMVVAGEGMDK	7.60E-13	RVAWSGIGVDLG	1.55E-11
Daldinia caldariorum	XP_047785741.1	AQGTVEINPRDLIVPT	8.90E-17	PPNARIADYVSY	7.71E-12	HNAGFGAVNH	3.38E-19	VPMVVAGEGMDK	7.60E-13	RVAWSGIGVDLG	1.55E-11
Daldinia childiae	XP_033438607.1	AQGTVEINPYDLIVPT	1.26E-16	PVNARIANYLSY	3.64E-10	HNAGFGAVNH	4.36E-19	VPMVVAGEGMDK	7.60E-13	RVAWSGIGIDLG	9.34E-12

Daldinia decipiens	XP_049095979.1	AQGTVEINPYDLIVPT	1.41E-16	PPNVRIADYISY	2.22E-12	HNGGFGAVNH	1.53E-19	VPMVVAGEGMDK	7.60E-13	RVAWSGIGVDLG	1.55E-11
Daldinia eschscholtzii	KAI1475320.1	AQGTVEINPYDLIVPT	4.37E-17	PPNARIADYLSY	1.01E-12	HNAGFGAVNH	3.38E-19	VPMVVAGEGMDK	7.60E-13	RVAWSGIGVDLG	1.55E-11
Daldinia grandis	KAI0095932.1	AQGTVETNPYDLIVPT	6.26E-17	PPNARIADYLSY	1.01E-12	HNAGFGAVNH	5.76E-17	VSMVVAGEGMDK	9.28E-11	RVAWSGIGLDLG	1.58E-10
Daldinia loculata	KAI2777984.1	AQGTIEINPYDLIVPT	2.45E-16	PPNARIADYLSY	1.01E-12	HNAGFGAVNH	3.38E-19	VPMVVAGEGMDK	7.60E-13	RVAWSGIGVDLG	1.55E-11
Daldinia vernicosa	XP_047863139.1	AQGTVEINPYDLIVPT	1.26E-16	PPNARIADYLSY	1.01E-12	HNAGFGAVNH	3.38E-19	VPMVVAGEGMDK	7.60E-13	RVAWSGIGVDLG	1.55E-11
Annulohypoxylon bovei	KAI2463407.1	AQGTVEIDPHDLIIPT	8.40E-19	PPNARIADYLSY	1.01E-12	HNAGFGAVNH	3.38E-19	VPMVVAGEGMDK	7.60E-13	RVAWSGIGVDLR	3.58E-12
Annulohypoxylon moriforme	KAI1454171.1	AQGTVEINPHDLIIPT	9.80E-19	PPNARIADYLSY	1.01E-12	HNAGFGAVNH	3.38E-19	VPMVVAGEGMDK	7.60E-13	RVAWSGIGVDLG	1.55E-11
Annulohypoxylon nitens	KAI0896334.1	AQGTVEINPHDLIIPT	1.14E-18	PPNARIADYLSY	1.01E-12	HNAGFGAVNH	3.38E-19	VPMVVAGEGMDK	7.60E-13	RVAWSGIGVDLG	1.55E-11
Annulohypoxylon stygium	KAI1445084.1	AQGTVEINPHDLIIPT	1.14E-18	PPNARIADYLSY	1.01E-12	HNAGFGAVNH	3.38E-19	VPMVVAGEGMDK	7.60E-13	RVAWSGIGVDLG	1.55E-11
Annulohypoxylon truncatum	XP_047849137.1	AQGTVEINPHDLIIPT	3.67E-18	PANARIADYLSY	1.19E-12	HNAGFGAVNH	3.38E-19	VPMVVAGEGMDK	7.60E-13	RVAWSGIGVDLG	1.55E-11
Hypoxylon cercidicola	KAI1774420.1	AQGTVEINPHDLIIPT	9.80E-19	PSNARIADYLSY	7.19E-13	HNAGFGAVNH	4.36E-19	VPMVVAGEGMDK	7.60E-13	RVEWSGIGIDLG	2.31E-12
Hypoxylon crocopeplum	KAI1377137.1	AQGTVEINPHDLIIPT	9.80E-19	PSNARVADYLSY	6.82E-14	HNAGFGAVNH	4.36E-19	VPMVVAGEGMDK	7.60E-13	RVAWSGVGVDLE	6.23E-11
Hypoxylon fragiforme	XP_049117066.1	AQGTVEINPHDLLIPT	8.37E-18	PGNSRVADYLNY	4.01E-10	HNAGFGAVNH	3.38E-19	VPMVVAGEGMDK	7.60E-13	RVAWSGIGVDLQ	2.83E-11
Hypoxylon fuscum	KAI1399455.1	AQGTVETNPEDLILPT	7.87E-16	PANARVADYLSY	1.49E-13	HNGGFGAVGH	5.21E-18	VPMVVAGEGMDK	7.60E-13	RVAWAGVGVDLG	8.16E-12
Hypoxylon rubiginosum	KAI4864951.1	AQGTVEINPHDLIIPT	3.67E-18	PGNARIADYLSY	7.71E-12	HNAGFGAVNH	4.36E-19	VPMVVAGEGMDK	7.60E-13	RVAWSGIGVDLG	1.55E-11
Hypomontagnella monticulosa	KAI0378476.1	AQGTVEINPHDLIIPT	3.67E-18	PPNARIADYLSY	1.01E-12	HNAGFGAVNH	4.36E-19	VPMVVAGEGMDK	7.60E-13	RVAWSGIGVDLG	1.55E-11
Hypomontagnella submonticulosa	KAI2620953.1	AQGTVEINPHDLIIPT	3.67E-18	PTNARIADYLSY	1.67E-11	HNAGFGAVNH	3.38E-19	VPMVVAGEGMDK	7.60E-13	RVAWSGIGVDLG	1.55E-11
Jackrogersella minutella	KAI1099809.1	AQGTVEINPHDLIIPT	3.67E-18	PPNARVADYLSY	1.19E-13	HNAGFGAVSH	3.33E-18	VPMVAAGEGMDK	7.26E-12	RIAWAGIGVDLE	1.75E-10
Rostrohypoxylon terebratum	KAI1090772.1	AQGTVEINPYDLIIPT	1.42E-17	PPNARIADYLSY	1.01E-12	HNAGFGAVNH	2.85E-17	VPMVVAGEGMDK	7.60E-13	RVAWSGIGVDLG	1.55E-11
Biscogniauxia marginata	KAI1498195.1	CQGTVATDLNQLVYPT	1.30E-15	PSNCKVEDYIPY	8.83E-12	TTGGYGAFQR	5.05E-16	NF		RAEWAGVAVNLR	8.67E-13
Biscogniauxia mediterranea	KAI1491404.1	AQGTVETNPHDLIIPT	4.47E-19	PPNAYIAPYLSY	7.15E-10	HNGGFGAVSH	1.87E-18	VPMVVAGEGMDK	7.60E-13	RVAWSGIGVDLR	3.58E-12
Durotheca rogersii	XP_051373960.1	AQGTVEINPHDLIIPT	9.80E-19	PPNTRIADYLSY	1.92E-12	HNAGFGAVNH	3.38E-19	VPMVAAGEGMDK	7.26E-12	RIAWSGIGIDLC	1.43E-10
Whalleya microplaca	KAI1080707.1	AQGTVETNPRDLIIPT	1.14E-18	PGNARVADYLSY	1.65E-12	HNAGFGAVNH	3.38E-19	VPMVVAGEGMDK	7.60E-13	RVAWSGIGVDLG	1.55E-11
Microdochium bolleyi	KXJ92914.1	SQGTVEINPLDLIIPT	8.66E-15	PSNARIADFLNY	1.89E-11	HNAGFGAVCH	1.17E-18	VPMVCAGEGMDK	1.90E-14	RVAWAGTGVDLQ	4.12E-10
Microdochium nivale	KAJ1329274.1	SQGTVETNPLDLIIPT	5.01E-15	PSNARIADFLNY	1.89E-11	HNAGFGAVCH	1.17E-18	VPMVCAGEGMDK	1.90E-14	RVAWAGVGVDLQ	1.55E-11
Microdochium trichocladiopsis	XP_046013910.1	AQGTVEINPHDLIIPT	1.41E-16	PGNARVADFLNY	3.44E-11	HNAGFGALCH	4.17E-18	VPMVCAGEGMDK	1.90E-14	RVAWAGAGVDVG	6.50E-10
Monosporascus cannonballus	RYO94743.1	CQGTVARDLDQLVIPT	8.90E-17	PPNARVVDYIPY	3.84E-14	SNAGLGALNH	2.65E-15	VPLVLAGITEDK	4.13E-12	NF	

Monosporascus ibericus	RYP11091.1	CQGTIAMDLNQLVIPT	1.09E-17	PPNARVADYIPY	2.50E-14	SNAGTGGLNH	1.23E-15	VPLVLAGVTEDK	3.68E-12	NF	
Pestalotiopsis fici	XP_007841187.1	AQGTVEVNPRQLIIPT	1.26E-16	PGNARIADYLSY	7.71E-12	HNAGFGAVNH	3.84E-19	VPMVVAGEGMDK	7.60E-13	RVTWSGIGVDLG	4.00E-11
Truncatella angustata	KAH8199344.1	AQGTVEINPNDLIIPT	1.09E-17	PDNVRVADYLLY	4.88E-11	HNAGFGAVNH	4.95E-19	VPMVAAGEGMDK	7.26E-12	RVAWSGIGVNLG	6.27E-12
Pseudomassariella vexata	XP_040718474.1	AQGTVEINPNDLIIPT	6.15E-19	PSNARIADYLSY	7.19E-13	HNAGFGAVNH	6.36E-17	VPMVVAGEGMDK	7.60E-13	RVAWSGIGVDLG	1.55E-11
Eutypa lata	EMR68577.1	NF		PANARVADYLPY	2.50E-14	SNAGYGALCH	2.85E-17	VPLVLAGETEDK	3.69E-13	RATWAGYALGLW	2.15E-7
Cercophora newfieldiana	KAK0638609.1	NF		PEGAKVMDYLPY	1.44E-18	FNGGYGGLMH	1.48E-18	TPMVIAGTAADK	3.29E-10	RAEWAGIAVNLR	1.21E-12
Cercophora samala	KAK0667676.1	TQGTVHRAYDELIIPV	5.49E-15	PENVRMVDYFPY	7.65E-11	SNAGYGGFMH	1.17E-19	VPMVLAGLVADK	1.96E-10	RAERAGVAVNLA	2.35E-9
Lasiosphaeria miniovina	KAK0706185.1	AQGTAMLDYSELIVPT	7.91E-15	PANTKVIDYLPY	8.52E-13	CNAGYGGFMH	1.24E-21	VPMVLAGTQADK	1.42E-10	RAEYVGVAVNLR	4.12E-10
Immersiella caudata	KAK0624188.1	NF		PGNAMVVDHVPY	7.61E-10	FNGGYGGLMH	5.21E-18	VPMVIAGTAADK	3.63E-10	RAEWAGVAVNLR	8.67E-13
Apiosordaria backusii	KAK0718985.1	SQGTVHRAYHELLIPT	3.78E-16	PKNVRIVDYFPY	5.89E-12	SNAGYGGFMH	2.84E-22	VPMVLAGLIADK	7.52E-10	RAARAGIAVNLA	6.11E-9
Collariella sp. IMI 366227	KAJ4286560.1	TQGTGHIEYTALIIPT	3.44E-15	PENTIVVDYFPY	2.98E-10	SNGGYGGVMH	5.47E-22	VPMVLAGTETDK	4.54E-10	RAEWAGVAVNLR	8.67E-13
Thermothielavioides terrestris	XP_003658264.1	TQGTAHCNYADLLIPT	2.13E-15	PANAIVVDYLPY	2.14E-11	SNAGYGGFMH	5.61E-19	GPMVLAGTAADK	3.09E-9	RGEYAGMAVNLR	1.58E-10
Staphylotrichum longicolle	KAG7294653.1	SQGTVHRVYDDLVIPT	5.01E-15	PDNAIVVDYFPY	1.18E-10	CNAGYNGFIH	2.65E-18	NF		RAEWAGVAVNLR	8.67E-13
Thermochaetoides thermophila	XP_006692471.1	SQGTLARDYSMLLAPT	1.59E-15	VGNVRVVDFIPY	4.88E-10	FNAGYGGFVH	2.66E-16	VPVIAAGLTEDK	5.66E-10	RVEWTGVGINLR	2.65E-13
Podospora anserina	XP_001904848.1	SQGTLARDYTMLLAPT	5.77E-16	LGNVKVVDFLPY	3.64E-10	FNAGYGGFVH	2.21E-16	VPVVAAGLTEDK	3.13E-10	RVEWTGAGINLR	7.23E-13
Echria macrotheca	KAK1751134.1	TQGTLSPIFTELLVPA	1.18E-13	PANTRVIDYLPY	5.10E-14	TNAGYNSFMH	5.61E-19	VPMLVAGKDADK	3.74E-9	RAEYCGVAVNLR	1.06E-11
Madurella mycetomatis	KXX79238.1	SQGTVHLDYREMITTA	4.03E-13	PDNTKIIDYLPY	1.30E-11	SNAGYGGFMH	2.84E-22	VPMVLSGAVADK	7.01E-10	RGEWAGVAVNLR	2.65E-13
Diaporthe ampelina	KKY35200.1	AQGTFANDFSDLILPT	1.07E-15	PPNARVADYLPY	1.62E-14	QSGSYGGFQH	1.73E-15	VPAVLGGDTEDK	2.58E-11	RAEWAGVGFNLK	4.13E-12
Diaporthe amygdali	XP_052998701.1	AQGTVETDPNDLIIPT	1.95E-19	SRNALVTDYLHY	1.14E-8	HNGGYGAITH	2.65E-21	VPMVVAGEGQDK	4.95E-13	RVRFSGIGVGLG	1.31E-8
Diaporthe batatas	XP_044648057.1	AQGTFANDFNDLILPT	6.40E-16	PSNARVADYLPY	4.76E-15	QAGSYGGFQH	4.74E-14	VPAVLAGETEDK	7.26E-12	RAEWAGVGISLK	4.74E-12
Diaporthe eres	KAI7784403.1	AQGTVETDPNDLIIPT	1.95E-19	PCNALVTDYLHY	1.37E-9	HNGGYGAITH	2.65E-21	VPMVVAGEGQDK	4.95E-13	NF	
Diaporthe helianthi	POS81165.1	AQGTFANDFNDLILPT	6.40E-16	PPNARVADYLPY	1.62E-14	QAGSYGGFQH	4.74E-14	VPAVLAGDTEDK	1.02E-11	RAEWAQVGLSLK	2.16E-9
Diaporthe ilicicola	KAI3399409.1	TQGTFATDPTSLIIPA	6.15E-19	PSNLRLASWLPY	4.25E-9	TNGGYGSVTQ	1.34E-19	VPLVCAGTSEDK	3.69E-13	NF	
Valsa sordida	ROV93292.1	AQGTVETDPNDLIIPT	9.56E-18	PGNALVTDYLSY	2.70E-10	HNGGYGAITH	2.65E-21	VPMVVAGEGQDK	4.95E-13	NF	
Cytospora leucostoma	ROW16112.1	AQGTVETDPNDLIIPT	1.95E-19	PGNALVTDYLNY	1.37E-9	HNGGYGAITH	3.80E-21	VPMVVAGEGQDK	4.95E-13	RVRFSGIGVGLG	1.31E-8
Coniella lustricola	PSR88517.1	AQGTVETNPNELILPT	9.56E-18	PSNARITDYLHY	2.14E-11	HNGGYGAITH	3.80E-21	VPMVVAGEGQDK	4.95E-13	RIRYSGIGVGLG	1.31E-8
Gnomoniopsis smithogilvyi	KAJ4397375.1	AQGTVETNPHDLIIPT	1.79E-18	PKNARVTDYLHY	1.47E-11	HNGGYGAITH	3.80E-21	VPMVVAGEGQDK	4.95E-13	NF	

Cryphonectria parasitica	XP_040776216.1	AQGTVETNPYDLIIPT	1.79E-18	PTNARVTDYLHY	7.65E-11	HNGGYGAITH	2.65E-21	VPMVVAGEGQDK	4.95E-13	RVRYSGIGVGLG	5.65E-9
Pyricularia grisea	KAI6379156.1	AQGTVAVKYNDLIIPV	3.40E-16	PTNTKVVDFMPY	1.31E-10	INAGYGGFTQ	6.66E-20	VPMVFSGDTEDK	6.49E-12	RGQWAGVGYNLR	1.44E-12
Pyricularia oryzae	KAI6251849.1	AQGTVALDYNKLAVPA	5.01E-15	PANARVVDVLPY	1.14E-11	INFGYGGFTQ	2.66E-16	FPMLLAGDTEDK	6.54E-11	RGEWAGVGYNLR	5.94E-13
Magnaporthiopsis poae	KLU84765.1	TQGTYAVDPTALIFPA	2.59E-15	NF		TNGGYGSVTQ	2.75E-20	VPLVCAGASEDK	3.24E-12	NF	
Gaeumannomyces tritici	XP_009229452.1	AQGTVSTSPHELLAPT	6.20E-14	PANARVLDYFPY	5.89E-12	HNAGYGGVQQ	7.24E-18	VPLVAAGQNEDK	5.01E-11	RIQCAGVGYNLA	2.16E-9
Sporothrix brasiliensis	XP_040617365.1	TQGTIATNTDLLVKPT	9.72E-13	PANVRTAAFVPH	3.62E-8	TNAGYNGVLA	6.04E-16	VPLVCAGRTEDK	4.95E-13	RVAWSGAGIDLA	4.48E-11
Sporothrix schenckii	XP_016584143.1	TQGTIATNTDLLVKPT	9.72E-13	PANVRTAAFVPH	3.62E-8	TNAGYNGVLA	6.04E-16	VPLVCAGRTEDK	4.95E-13	RVAWSGAGIDLA	4.48E-11
Grosmannia clavigerum	XP_014175810.1	TQGTIATSYSDLLIPT	9.56E-18	PANARVVDYLPY	3.84E-14	LNAGYGGLIH	1.67E-18	VPMVLAGSTEDK	7.60E-13	RGQCAGVAVNLR	8.58E-11
Ophiostoma piceae	EPE10437.1	TQGTISVNTDLLVKPT	7.28E-13	PANVRTAPFVPH	2.48E-8	TNAGYNGTLT	1.73E-15	VPLVCAGRSEDK	1.50E-12	RVAWSGAGIDLG	1.37E-11
Coniochaeta hoffmannii	KAJ9155879.1	AQGTVETDPWELILPT	7.92E-17	PSNARVTDYLHY	5.16E-12	HNGGYGAVQH	2.20E-21	VPMVVGGEGQDK	1.76E-12	RIAYSGIGVNLG	1.93E-10
Coniochaeta ligniaria	OIW23813.1	AQGTVETDPWELILPT	7.92E-17	PSNARVTDYLHY	5.16E-12	HNGGYGAVQH	2.20E-21	VPMVVAGEGQDK	4.95E-13	RMAYSGAGVSLG	8.31E-9
Coniochaeta pulveracea	RKU48537.1	AQGTVETDPNQLVIPT	8.37E-18	PTIRRLVDHASY	1.88E-5	HNGGYGAVQH	2.20E-21	VPMVIAGEGQDK	1.76E-12	RLAYSGAGVDLK	1.68E-9
Cephalotrichum gorgonifer	SPO03607.1	TQGTVVSNFDNLVKPV	2.56E-13	PRNARVQDYLLY	1.25E-9	SNGGYGGFMQ	3.20E-20	VPMVLAGTTQDK	1.46E-13	RGEWAGIAVNLR	4.06E-13
Thozetella sp. PMI_491	KAH8901217.1	SQGTAHVDYSQLIVPT	8.90E-17	PQNVRWTDFLAY	8.25E-9	CNAGYGSTSH	8.63E-16	TPAVLAGEVLDK	5.80E-9	NF	
Thyridium curvatum	XP_030999529.1	SQGTAEVDYGQLIVPT	9.99E-17	PDNVRCADFLAY	7.15E-10	CNAGYGSTSH	1.83E-16	VPAVLAGEVLDK	7.01E-10	NF	
Phialemonium atrogriseum	KAK1762806.1	AQGTVAVDFMDLVIPT	2.74E-16	PANARVIDYLPY	9.57E-15	MNGGYGGVMH	2.02E-20	VPLVLAGDTEDK	5.67E-13	RAEWAGVAVNLR	8.67E-13
Phaeoacremonium minimum	XP_007916963.1	TQGTVNNKPDQLIQPA	1.48E-14	PSNARVIDYLPY	9.29E-16	FNGGFGGYMH	5.21E-18	IPMVMAGISEDK	1.55E-11	RAEWAGVAVNLR	8.67E-13
Pleurostoma richardsiae	KAJ9149742.1	AQGTVEFDPHDLIIPT	6.39E-18	PANVRVADYLSY	3.38E-13	HNAGYGAVTH	1.06E-20	VPMVVAGEGQDK	4.95E-13	NF	
Ascochyta clinopodiicola	KAJ4346241.1	TSSTVVFDNNVLIIPA	7.69E-12	PENARVTKFFPL	8.25E-9	TNGGYGTIQQ	9.00E-18	VPMIVSGVGQDK	7.14E-11	NF	
Ascochyta lentis	KAF9695519.1	TSSTVVFDNNVLIIPT	3.21E-12	PDNARVTKFIPL	3.29E-9	TNGGYGTIQQ	2.32E-17	VPMIVSGVGQDK	7.14E-11	NF	
Ascochyta rabiei	XP_038797369.2	TSSTVVFDNNVLIIPA	7.69E-12	PENARVTKFFPL	8.25E-9	TNGGYGTIQQ	9.00E-18	VPMIVSGVGQDK	7.14E-11	NF	
Didymella heteroderae	KAF3045961.1	SQGTVETDLSNLVLPT	3.20E-14	PHNARVAKFVPY	7.65E-11	INGGYGAIVQ	2.89E-15	VPVVVSGKGQDK	7.52E-10	IIQWSGVGIDIG	4.83E-9
Didymella pomorum	KAJ4411464.1	SQGTVETDLSDLLLPT	5.20E-16	PRNARVAKFVPY	5.47E-11	NNGGYGAIIQ	5.62E-15	IPVVVSGEGQDK	5.66E-10	IIQWSGVGVDIG	6.60E-9
Boeremia exigua	XP_046000075.1	SQGTVETDLSNLVLPT	3.78E-16	PHNARVAKFVPY	7.65E-11	NNGGYGAVIQ	3.16E-17	VPLVVGGEGQDK	1.26E-11	IVQWSGVGIHVG	2.18E-8
Macroventuria anomochaeta	XP_033560487.1	SQGTIETDLNNLVLPT	4.21E-16	PQNARVAKFVPY	5.47E-11	NNGGYGAVIQ	1.67E-16	TPLVVGGEGQDK	2.29E-10	IVQWSGVGIHVG	2.18E-8
Epicoccum nigrum	OSS46826.1	SQGTVETDLTNLVLPT	1.26E-16	PANARVAKFVPY	1.01E-11	NNGGYGAVIQ	3.16E-17	VPLVVAGEGQDK	4.67E-12	IVQWTGVGLNIG	1.51E-8
Stagonospora sp. SRC1lsM3a	OAL04400.1	SQGTVGTNLNDLLIPT	2.59E-15	PKNARVAKFVPY	1.89E-11	NNGGYGAVIQ	6.61E-16	IPMVVAGKGQDK	5.96E-11	NF	

Byssothecium circinans	KAF1948264.1	**SQGTLEVNPENLVLPT**	1.97E-16	**PSNARVTKFVPF**	2.44E-10	**SNGGYVAVQH**	3.71E-15	**VPMVVSGTTQDK**	4.67E-12	**IIEYTGVGINLR**	4.52E-10
Massarina eburnea	KAF2646682.1	**SQGTVEVRPEDLVLPA**	2.85E-15	**PSNARVAKFVPY**	6.73E-12	**TNGGYGAVQH**	2.36E-18	**VPMVVSGEGQDK**	5.75E-12	**IIEYTGVGINLK**	3.42E-10
Bipolaris maydis	XP_014078238.1	**SQGTIAVNFNDLVVPT**	7.04E-17	**PSNARVEGFIPF**	3.30E-10	**TNGGYGSVQH**	4.95E-19	**VPLVLAGASEDK**	1.72E-11	**RAEWAGVAVNLR**	8.67E-13
Bipolaris oryzae	XP_007682054.1	**SQGTIAVNFNDLVVPT**	7.04E-17	**PSNARVEGFIPF**	3.30E-10	**TNGGYGSVQH**	3.84E-19	**VPLVLAGASEDK**	1.72E-11	**RAEWAGVAVNLR**	8.67E-13
Decorospora gaudefroyi	KAF1836052.1	**SQGTVETNFDELLIPT**	4.37E-17	**RGNVRATRFVPY**	3.00E-7	**SNGGFGAVMT**	1.59E-15	**IPVVVAGSGQDK**	7.01E-10	**NF**	
Kalmusia sp. IMI 367209	KAJ4293461.1	**SQGTVETKPEEIIIPT**	1.75E-15	**PDNARVARYIPH**	8.54E-11	**ANGGFGAVQK**	7.34E-10	**IPMVLSGVGQDK**	1.10E-10	**NF**	
Karstenula rhodostoma	KAF2448746.1	**SQGTVETNPNDLILPT**	9.80E-19	**PNNARVARYIPH**	1.31E-10	**GNGGFGGVQK**	9.82E-10	**IPMVISGVAQDK**	1.96E-9	**IADFTGVGINLG**	8.97E-9
Paraphoma chrysanthemicola	KAH7071542.1	**SQGTLGTNMGDLLLPT**	1.61E-14	**PKNTRVAKFVPY**	3.06E-11	**NNGGFGAVIS**	1.37E-14	**IPMVLAGTGQDK**	6.49E-12	**NF**	
Setomelanomma holmii	KAF2023082.1	**SQGTVAMEFGDLLLST**	2.70E-14	**PRNARVAKFVPY**	5.47E-11	**NNGSYGAVIT**	4.74E-14	**VPMVLAGTGQDK**	5.67E-13	**NF**	
Pyrenochaeta sp. DS3sAY3a	OAL46984.1	**SQGTVDTNLNEILIPT**	8.66E-15	**PVNARAAKFVPY**	3.29E-9	**SNGGYGAVMT**	2.89E-15	**VPLVVGGEGQDK**	1.26E-11	**NF**	
Cucurbitaria berberidis	XP_040790731.1	**SQGTLETQWDDLLFPT**	4.46E-14	**PANARAAKFVPY**	5.37E-10	**NNGGYGAVMT**	8.03E-14	**IPVVVAGDGQDK**	1.67E-10	**NF**	
Clohesyomyces aquaticus	ORY16921.1	**TQGTVTVDYTELLIPT**	5.24E-19	**GANVRVLDYFSY**	1.14E-10	**SNAGFGTYLH**	2.09E-17	**VPMVLAGTKLDK**	1.01E-10	**RAEYCGIAIDLK**	1.37E-11
Dendryphion nanum	KAH7135333.1	**SQGTVAPVLSEIVLPT**	2.12E-12	**PENARVAKFIPY**	1.01E-11	**SNGGYGAVQH**	2.32E-17	**IPMVVSGIGQDK**	1.96E-10	**IVQWSGVGLNLE**	1.99E-9
Lophiostoma macrostomum	KAF2648898.1	**SQGTAETNLEHLVLPT**	1.76E-14	**PGNVRVAKFVPY**	7.65E-11	**SNGGYGAVLH**	9.40E-17	**IPMVVAGMGQEK**	2.68E-10	**IIEWSGVGINLK**	1.98E-11
Lophiotrema nucula	KAF2113514.1	**SQGTVETNPEDLVLPT**	7.92E-17	**PANTRVAKFVPY**	1.67E-11	**SNGGYGAVQH**	4.72E-17	**IPMVVSGLSQDK**	3.13E-10	**NF**	
Melanomma pulvis-pyrius	KAF2786108.1	**TQGTVTVDYSELLIPA**	1.42E-17	**GANVRVLDYFSY**	1.14E-10	**TNAGFGSYLH**	1.32E-18	**VPMVLAGTKLDK**	1.01E-10	**RAEYCGIAIDLK**	1.37E-11
Polyplosphaeria fusca	KAF2731913.1	**SQGTVGLDMNDLLLPT**	5.49E-15	**PANTRVEKFVPY**	4.35E-11	**SNGGYGAVQQ**	4.21E-16	**IPMVVGGIVQDK**	2.48E-10	**LIQYTGVGIDLR**	4.13E-9
Trematosphaeria pertusa	XP_033681098.1	**SQGTVEVRPEDLVLPT**	1.94E-15	**PKNARVAKFVPY**	1.89E-11	**SNGGYGAVQH**	2.32E-17	**IPMVVAGEGQDK**	5.75E-12	**LVDFTGVGIDLK**	8.97E-9
Periconia macrospinosa	PVH96591.1	**TQGTVSVDYSELLIPA**	1.61E-17	**GANVRVLDYFSS**	8.09E-8	**TNAGFGSYLH**	1.32E-18	**VPMVLAGTKLDK**	1.01E-10	**RAEYCGIAIDLK**	1.37E-11
Botryosphaeria dothidea	KAF4305872.1	**SQGTLDVDLQQLVLPT**	2.94E-14	**PHNVRVEGWIPY**	3.64E-10	**TNGGYGSLQH**	6.66E-20	**VPLVIAGGSMDK**	4.58E-11	**RAEWAGLGVNLR**	6.27E-12
Neofusicoccum parvum	EOD53031.1	**SQGTVDVDLSQLVLPT**	4.16E-15	**PRNARVEGWIAY**	3.59E-9	**TNGGYGSLQA**	1.25E-16	**VPLVLAGGSIDK**	1.54E-10	**RAEWAGLGVNLR**	6.27E-12
Macrophomina phaseolina	EKG11414.1	**SQGTIDVDLHQLVLPT**	6.02E-15	**PANARVEGWIPY**	3.87E-11	**TNGGYGSLSQ**	7.01E-17	**VPLVVAGGSIDK**	2.48E-10	**RAEWAGLAVNLR**	1.37E-11
Aureobasidium melanogenum	KAG9597102.1	**NF**		**PANARIASYLPF**	3.64E-10	**TNGGYNSVNQ**	7.23E-16	**IPIVAAGVTEDK**	9.97E-10	**RIAWSGAGINLR**	5.45E-12
Mytilinidion resinicola	XP_033568734.1	**TAGSIDNNPEDLILPT**	5.43E-13	**PANARMAKFIPY**	3.87E-11	**SNGGYGTVQQ**	4.27E-17	**VPMVLAGVGQDK**	2.24E-12	**NF**	
Lophium mytilinum	KAF2497128.1	**TSGSIDNNPEDLILPT**	1.49E-13	**PVNARMAKFIPY**	3.30E-10	**SNGGYGTVQQ**	2.57E-17	**VPMVLSGVGQDK**	1.91E-11	**VAAWAGVAIDLQ**	4.13E-9
Glonium stellatum	OCL02246.1	**SSGSVDNNPEDLIIPT**	1.48E-14	**PSNARIAKFVPF**	2.20E-10	**SNGGYGTVQQ**	7.01E-17	**VPMVLAGVGQDK**	2.24E-12	**IADWAGVAINLA**	1.20E-10
Zopfia rhizophila	KAF2191997.1	**SQGTVEANPKDLTLPT**	1.35E-14	**PKNARIAKFVPY**	6.84E-11	**SNGGYGAVQH**	2.85E-17	**IPMVVSGIGQDK**	1.96E-10	**LVEWSGVGINLK**	2.83E-11

Aspergillus awamori	GCB23152.1	TQGTVKIHYKDLLIPT	4.93E-17	PDNAQVIDYLSY	4.35E-11	TNGGYGSFMH	3.42E-24	VPMVLAGTVQDK	3.24E-12	RGEWAGIGINLR	2.76E-14
Aspergillus brasiliensis	GKZ22376.1	TQGTIRTYYRELIIPA	1.41E-16	PDNAKVIDYLSY	3.93E-12	TNGGYGSFMH	3.42E-24	VPMVLAGTVQDK	3.24E-12	RGEWAGIGINLR	2.76E-14
Aspergillus carbonarius	OOG00364.1	TQGTFRIDYNELIIPT	1.54E-18	PKNAKVIDYLLY	7.65E-11	TNGGYGSFTH	1.33E-24	VPMVMAGTGQDK	7.60E-13	RVEWAGIGVNLR	7.17E-14
Aspergillus carlsbadensis	KAJ0415219.1	SQGTFQVDYDMLLKPS	2.48E-14	PANTRVVDFLSY	1.19E-12	SNAGYGGFLH	2.65E-21	VPMVLAGSGQDK	4.13E-12	RGEWAGIAVNLK	2.10E-13
Aspergillus costaricensis	XP_025540426.1	TQGTVKTYYKNLLIPT	5.56E-17	PDNAKVIDYLSY	3.93E-12	TNGGYGSFMH	3.42E-24	VPMVLAGTVQDK	3.24E-12	RGEWAGIGINLR	2.76E-14
Aspergillus eucalypticola	XP_025383912.1	TQGTIKTHYKDLVIPT	4.37E-17	PDNAKVIDYLSY	3.93E-12	TNGGYGSFMH	3.42E-24	VPMVLAGTVQDK	3.24E-12	RGEWAGIGINLR	2.76E-14
Aspergillus hancockii	KAF7587272.1	TQGTFRVDHRELLLPT	3.44E-15	PSNAKVIDYLSY	7.19E-13	TNGGYGSFMH	1.42E-22	VPMVMAGTGQDK	7.60E-13	RGEWAGIAVNLQ	5.45E-12
Aspergillus homomorphus	XP_025552456.1	TQGTFILDYRMLLIPT	2.74E-16	PENAKVVDYLSY	1.14E-11	TNGGYGGFLH	1.12E-22	VPMVIAGTGQDK	4.13E-12	RGEWAGIAANLR	3.18E-11
Aspergillus ibericus	XP_025578007.1	TQGTFRIDYNELVIPT	3.42E-17	PSNAKVVDYLLY	4.35E-11	TNGGYGSFTH	1.33E-24	VPMVMAGTGQDK	7.60E-13	RVEWAGIGVNLR	7.17E-14
Aspergillus luchuensis	OJZ81278.1	TQGTDKTYYKNLLIPT	2.13E-15	PNNTKVIDYLSY	1.14E-11	TNGGYGGFMH	3.42E-24	VPMVLAGTVQDK	3.24E-12	RGEWAGIGINLR	2.76E-14
Aspergillus mulundensis	XP_026604105.1	SQGTVMVDYNMLLIPT	2.67E-17	PANAKVVDYLLY	6.12E-11	NNGGYGGFMQ	1.82E-21	VPMVLAGVELDK	3.91E-10	RGEWAGIAVNLR	4.06E-13
Aspergillus neoniger	XP_025484262.1	TQGTVKIYYKNLLIPT	1.12E-16	PDNAKVIDYLSY	3.93E-12	TNGGYGSFMH	3.42E-24	VPMVLAGTVQDK	3.24E-12	RGEWAGIGINLR	2.76E-14
Aspergillus niger	GKZ64237.1	TQGTVKIHYKDLLIPT	4.93E-17	PDNAQVIDYLSY	4.35E-11	TNGGYGSFMH	3.42E-24	VPMVLAGTVQDK	3.24E-12	RGEWAGIGINLR	2.76E-14
Aspergillus piperis	XP_025513870.1	TQGTDKTYYKNLLIPT	2.13E-15	PNNTKVIDYLSY	1.14E-11	TNGGYGGFMH	3.42E-24	VPMVLAGTVQDK	3.24E-12	RGEWAGIGINLR	2.76E-14
Aspergillus sclerotiicarbonarius	PYI02360.1	TQGTFRIDYTELIIPT	7.19E-19	PNNAKVVDYLSY	1.01E-11	TNGGYGSFTH	1.33E-24	VPMVMAGTGQDK	7.60E-13	RVEWAGIGVNLR	7.17E-14
Aspergillus sclerotioniger	XP_025463962.1	TQGTFRIDYKELIIPT	2.36E-17	PSNARVVDYLLY	8.83E-12	TNGGYGSFTH	1.33E-24	VPMVMAGTGQDK	7.60E-13	RVEWAGIGINLR	1.95E-14
Aspergillus tubingensis	GLB04893.1	TQGTVKTYYKNLLIPT	5.56E-17	PDNAKVIDYLSY	3.93E-12	TNGGYGSFMH	3.42E-24	VPMVLAGTVQDK	3.24E-12	RGEWAGVGINLR	1.29E-14
Aspergillus versicolor	UZP48228.1	SQGAVNNDPTDLILPT	6.40E-16	PSNVKLAEFIPF	8.25E-9	SNGGFGTVQM	5.05E-16	VPMVLAGVYLDK	2.48E-10	NF	
Aspergillus welwitschiae	XP_026624638.1	TQGTVKIHYKDLLIPT	7.23E-15	PDNAQVIDYLSY	4.35E-11	TNGGYGSFMH	3.42E-24	VPMVLAGTVQDK	3.24E-12	RGEWAGIGINLR	2.76E-14
Penicilliopsis zonata	XP_022577075.1	TQGTISLDYTALLVPT	3.05E-16	PANARVLDYLAY	1.18E-10	SNGGYGGFLH	1.34E-19	VPMVLAGEGQDK	2.02E-13	RGEWAGIAVDLK	8.67E-13
Penicillium alfredii	XP_056509782.1	TQGTVAIDYDMLLIPA	1.61E-17	PVNAKVVDYLSY	2.71E-11	TNGGYGSFMH	4.73 E-24	VPMVMAGTTQDK	2.02E-13	RGEWAGIAVNLR	4.06E-13
Penicillium bovifimosum	XP_056526610.1	SQGAVNNDPTDLILPT	6.40E-16	PSNVKLADFIPF	3.64E-10	SNGGYGTVQM	1.34E-15	VPMVLAGVYLDK	2.48E-10	NF	
Penicillium macrosclerotiorum	XP_056934836.1	AQGTFILDYRMLLIPT	2.59E-15	PNNAKVIDYFPY	7.71E-12	TNGGYGGFLR	4.36E-19	VPMVIAGTGQDK	4.13E-12	RGEWAGIAANLR	3.18E-11
Penicillium odoratum	XP_057000407.1	SQGSVHNDPEELILPA	3.78E-15	PSNVRIARFIPF	1.79E-10	SNGGFGTVQQ	1.11E-17	VPMVLAGMSADK	3.43E-11	HAAWAGAAINLA	2.55E-9
Talaromyces amestolkiae	XP_040736898.1	TQGTFLLDYNMLLKPT	1.04E-14	PRNTKIADYLPY	3.44E-11	SNAGYGGFLH	2.36E-20	VPMVLAGRGQDK	2.24E-12	RGEWAGIALNLK	7.14E-12
Talaromyces atroroseus	XP_020121501.1	TQGTIAVDYNALLIPT	7.32E-18	PSNAKVVDYLMY	5.47E-11	TNAGYGGFIS	2.29E-19	VPMVWAGRGQDK	3.43E-11	RGEIAGIGVNLE	2.35E-9
Cladophialophora carrionii	XP_008725802.1	SQGTLNINVTDLIIPT	2.74E-16	PDNVRVASYLPF	4.01E-10	SNAGYGTVQQ	4.72E-17	VPMVLAGVGEDK	1.35E-12	RAAWAGVAVNLK	2.67E-12

Cladophialophora chaetospira	KAJ9615145.1	**SQGTLNINVTDLIIPT**	2.74E-16	**PKNAHVARYLPF**	1.46E-10	**SNAGYGTVQQ**	4.72E-17	**VPMVLAGVGEDK**	1.35E-12	**RAAWAGVAINLK**	1.44E-12
Phaeomoniella chlamydospora	KKY20074.1	**NF**		**PSNVLVAKFIPF**	5.91E-10	**NNGGYGTVQT**	1.87E-14	**VPMVLAGQTEDK**	7.46E-15	**RAAWSGAAINLA**	8.58E-11
Botryotinia calthae	TEY37428.1	**SQGTVALNYEDLIIPT**	9.47E-21	**PKNVYVEDFLPF**	1.99E-10	**TNGGYGGFQH**	4.22E-25	**VPMVMGGDTEDK**	2.25E-13	**RCEWIGVGVNLK**	7.23E-13
Botryotinia convoluta	TGO51629.1	**SQGTVALNYGDLIIPT**	3.85E-20	**PKNTYVEDFIPF**	2.20E-10	**TNGGYGGFQH**	4.22E-25	**VPMVMGGDTEDK**	2.25E-13	**RCEWIGVGVNLK**	7.23E-13
Botryotinia globosa	KAF7901251.1	**SQGTVALNYGDLIIPT**	3.85E-20	**PKNAYVEDFIPF**	1.46E-10	**TNGGYGGFQH**	5.72E-26	**VPMVMGGETEDK**	1.46E-13	**RCEWIGAGVNLK**	1.68E-12
Botryotinia narcissicola	TGO56301.1	**SQGTVALNYGDLIIPT**	3.85E-20	**PKNAYVEDFIPF**	1.46E-10	**TNGGYGGFQH**	4.22E-25	**VPMVMGGETEDK**	1.46E-13	**RCEWIGAGVNLK**	1.68E-12
Botrytis aclada	KAF7956861.1	**SQGTVALNYGDLIIPT**	3.85E-20	**PKNAYVEDFLPF**	1.31E-10	**TNGGYGAFQH**	4.41E-22	**IPMVMGGDTEDK**	3.24E-12	**RCEWIGVGVNLK**	7.23E-13
Botrytis byssoidea	XP_038733006.1	**SQGTVALNYADLIIPT**	3.85E-20	**PKNAYVEDFIPF**	1.46E-10	**TNGGYGGFQH**	4.22E-25	**VPMVMGGETEDK**	1.46E-13	**RCEWIGAGVNLK**	1.68E-12
Botrytis cinerea	EMR81961.1	**SQGTVALDYGDLIIPT**	3.17E-20	**PKNAYVEDFLPF**	1.31E-10	**TNGGYGGFQH**	4.22E-25	**VPMVMGGDSEEK**	1.13E-11	**RGEWIGVGVNLK**	1.21E-12
Botrytis deweyae	XP_038811764.1	**SQGTVALNYGDLVIPT**	4.47E-19	**PKNAYIEDFIPF**	4.43E-10	**TNGGYGGFQH**	4.22E-25	**VPMVMGGDTEDK**	2.25E-13	**RCEWIGVGVNLK**	7.23E-13
Botrytis fragariae	XP_037193260.1	**SQGTVALNYGDLIIPT**	3.85E-20	**SKNTYVEDFIPF**	3.29E-9	**TNGGYGGFQH**	5.72E-26	**VPMVMGGDTEDK**	2.25E-13	**RCEWIGVGVNLK**	7.23E-13
Botrytis galanthina	THV45154.1	**SQGTVALNYGDLIIPT**	3.85E-20	**PKNVYVEDFLPF**	1.99E-10	**TNGGYGGFQH**	4.22E-25	**VPMVMGGDTEDK**	2.25E-13	**RCEWIGVGVNLK**	7.23E-13
Botrytis paeoniae	TGO20471.1	**SQGTVALNYGDLIIPT**	3.85E-20	**PKNTYVEDFIPF**	2.20E-10	**TNGGYGGFQH**	4.22E-25	**VPMVMGGDTEDK**	2.25E-13	**RCEWIGVGVNLK**	7.23E-13
Botrytis porri	XP_038768623.1	**SQGTVALNYGDLIIPT**	3.85E-20	**PKNAYVEDFLPF**	1.31E-10	**TNGGYGAFQH**	3.42E-24	**VPMVMGGDTEDK**	2.25E-13	**RCEWIGVGVNLK**	7.23E-13
Botrytis sinoallii	XP_038760845.1	**SQGTVALNYGDLVIPT**	4.47E-19	**PKNAYVEDFIPF**	1.46E-10	**TNGGYGGFQH**	4.22E-25	**VPMVMGGDTEDK**	2.25E-13	**RCEWIGVGVNLK**	7.23E-13
Botrytis tulipae	TGO09160.1	**SQGTVALNYGDLIIPT**	3.85E-20	**PKNAYVEDFIPF**	1.46E-10	**TNGGYGGFQH**	5.72E-26	**VPMVMGGETEDK**	1.46E-13	**RCEWIGAGVNLK**	1.68E-12
Sclerotinia borealis	ESZ95592.1	**SQGTIALNYEDLLIPT**	4.47E-19	**PSNAYVEEFVPF**	5.94E-9	**TNGGYGGFQH**	1.12E-22	**CPMVMGGETEDK**	3.68E-12	**RGEWTGVGIDLK**	7.23E-13
Sclerotinia nivalis	KAJ8071588.1	**SQGTVAVKYEELIIPT**	8.40E-19	**PSNVRVEDFIPF**	1.47E-11	**TNGGYGAFRH**	1.72E-20	**VPMVMGGDAQEK**	1.67E-10	**RCEWAGVGINLR**	3.28E-15
Sclerotinia sclerotiorum	XP_001589362.1	**SQGTVAVKYEELIIPT**	3.19E-18	**PSNVHVEDFIPY**	5.89E-12	**TNGGYGSFRH**	1.53E-19	**VPMVIGGDTQEK**	2.83E-11	**RCEWAGAGVNMR**	6.27E-12
Sclerotinia trifoliorum	CAD6453695.1	**SQGTIAVKYEELIIPT**	7.32E-18	**PSNARVEDFIPF**	8.83E-12	**TNGGYGAFRH**	2.97E-19	**VPMVIGGGTQEK**	5.96E-11	**RCEWAGAGINMR**	3.58E-12
Monilinia laxa	KAB8303359.1	**SQGTVALKYEDLLIPT**	1.24E-17	**PKNAFVEDFIPF**	1.95E-9	**TNGGYGAFQH**	3.55E-22	**CPMVMGGDTEDK**	5.23E-12	**RCQWTGVGINLK**	2.65E-13
Stromatinia cepivora	KAF7872547.1	**SQGTVALKYEELLIPT**	3.03E-17	**PSNAYVEDFVPF**	2.44E-10	**TNGGYGAYQH**	6.43E-21	**VPMIMAGDTQEK**	6.49E-12	**RCEWLGTGINLR**	5.43E-10
Rhexocercosporidium sp. MPI-PUGE-AT-0058	KAH7346361.1	**SQGTATTMYEKLILPT**	3.13E-15	**PQNARVGDFIPF**	1.25E-9	**TNGGYGGFQH**	3.18E-21	**NF**		**RVEWAGLGVNMR**	4.48E-11
Pseudogymnoascus destructans	XP_024325152.1	**SQGTLNLNYTELIIPT**	3.80E-19	**PENARVVDFIPF**	2.71E-11	**TNGGYGAVQH**	1.02E-19	**VPMIIAGATEDK**	1.39E-11	**RAEWAGVAINLR**	4.06E-13
Pseudogymnoascus verrucosus	XP_018126712.1	**SQGTLNLNYAELIIPT**	6.39E-18	**PENARVVDFIPF**	2.71E-11	**TNGGYGAVQH**	1.04E-18	**IPMIIAGATEDK**	8.53E-11	**RAEWAGVAINLR**	4.06E-13
Claussenomyces sp. TS43310	KAI9732059.1	**CQGTLAMNYSDLVIPT**	8.09E-20	**PSNSRVADYIPF**	5.47E-11	**TNGGYGGFQH**	1.72E-20	**VPLVIGGSTEDK**	7.14E-11	**RAAWAGVGVNLQ**	1.55E-11
Alectoria fallacina	CAF9943470.1	**NF**		**PANVRVERFIPH**	2.44E-10	**TNAGFNGVLA**	9.42E-16	**VPLICAGISEDK**	4.67E-12	**NF**	

Alectoria sarmentosa	CAD6566374.1	**NF**		**PANVRVERFIPH**	2.44E-10	**TNAGFNGVLA**	9.42E-16	**VPLICAGISEDK**	4.67E-12	**NF**	
Lasallia pustulata	KAA6413478.1	**SQGTLSNEPEDLTIPA**	4.85E-14	**PSNVKIAQWIPF**	3.29E-9	**NNGGYGTINL**	3.77E-14	**VPLVLAGLSEDK**	2.12E-11	**RTAWTGAAIDLA**	2.76E-9
Pycnora praestabilis	KAI9822666.1	**SQGTASFDLNLLVVPT**	3.22E-13	**PANAKVAKFIPF**	9.51E-11	**SNGGYGTIQQ**	4.27E-17	**VPMVLAGMTEDK**	4.95E-13	**RTAWTGAAINLA**	1.55E-9
Xylona heveae	XP_018185306.1	**TQGTLIAEPSALILPA**	5.84E-13	**PPNVRIEPFIPH**	3.91E-9	**STGGYNGVKM**	1.12E-12	**NF**		**RISWAGAAINLR**	8.16E-12

Accession number: Unique identifier assigned to each biological sequence within the NCBI database. **p-value:** Probability that a random sequence (of the same length) would match the test motif with a score equal to or higher than the highest found in the test sequence. **NF**: Not found.

Figure S2. Motifs location types in putative Rht2 sequences.

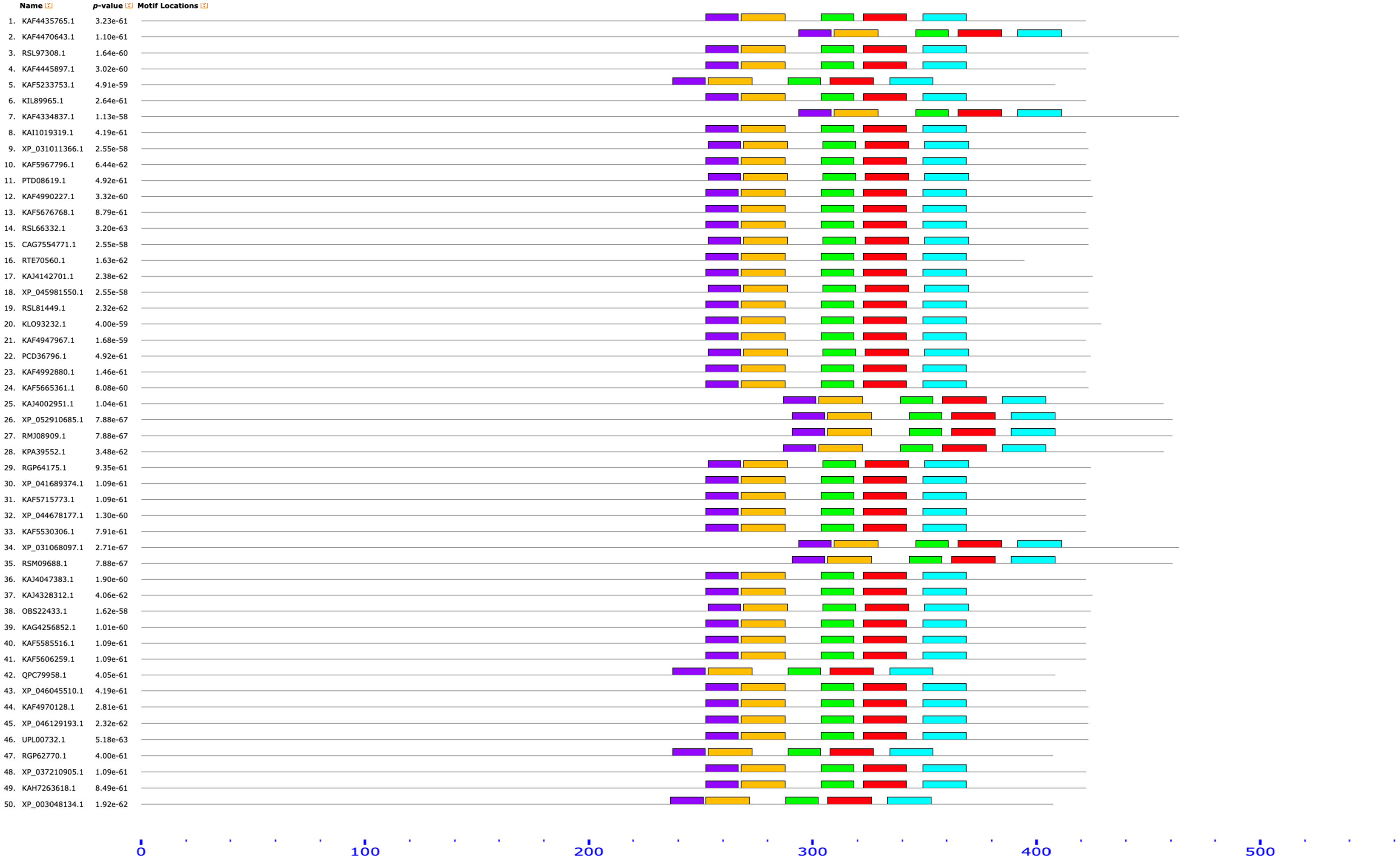

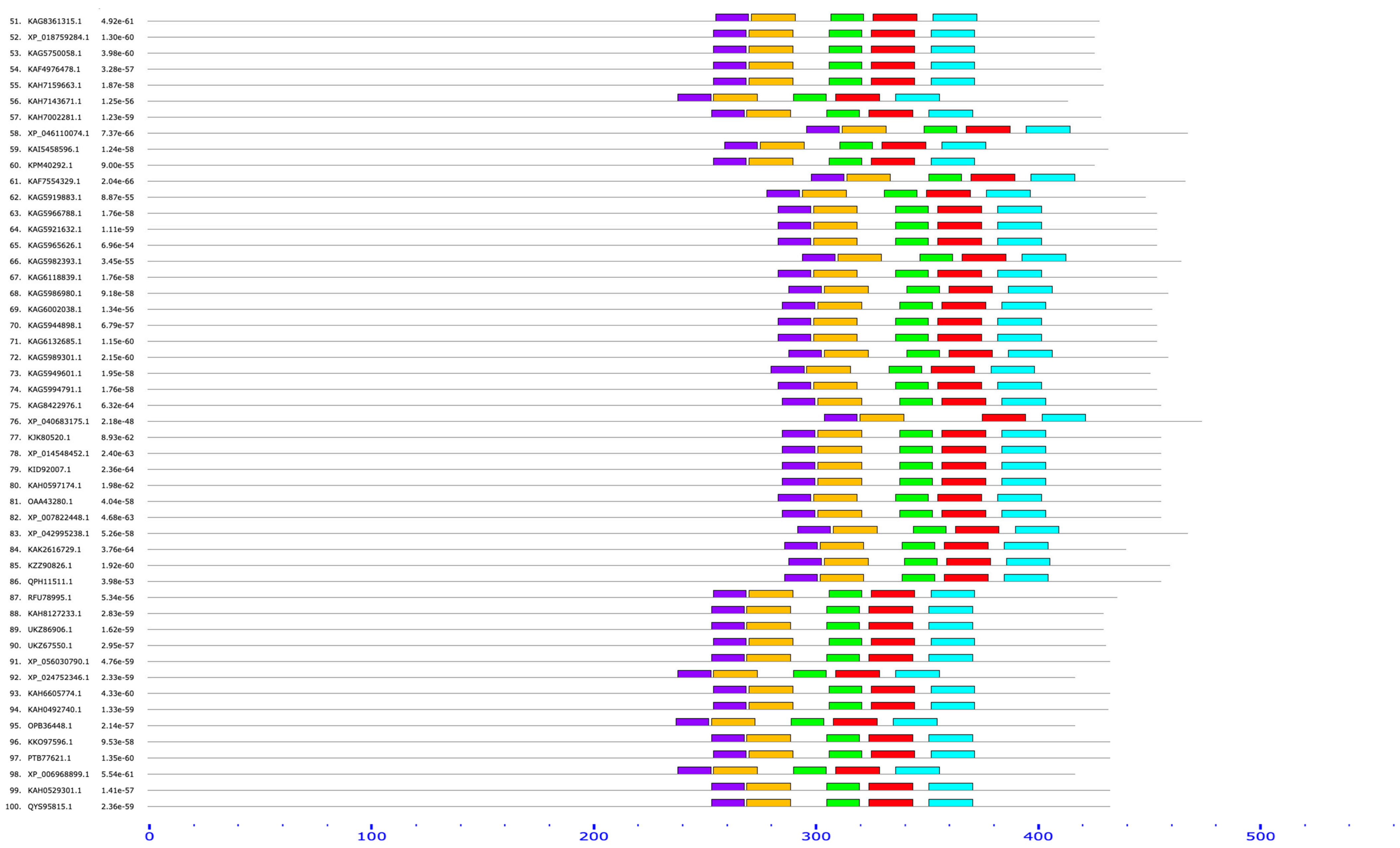
51. KAG8361315.1 4.92e-61
52. XP_018759284.1 1.30e-60
53. KAG5750058.1 3.98e-60
54. KAF4976478.1 3.28e-57
55. KAH7159663.1 1.87e-58
56. KAH7143671.1 1.25e-56
57. KAH7002281.1 1.23e-59
58. XP_046110074.1 7.37e-66
59. KAI5458596.1 1.24e-58
60. KPM40292.1 9.00e-55
61. KAF7554329.1 2.04e-66
62. KAG5919883.1 8.87e-55
63. KAG5966788.1 1.76e-58
64. KAG5921632.1 1.11e-59
65. KAG5965626.1 6.96e-54
66. KAG5982393.1 3.45e-55
67. KAG6118839.1 1.76e-58
68. KAG5986980.1 9.18e-58
69. KAG6002038.1 1.34e-56
70. KAG5944898.1 6.79e-57
71. KAG6132685.1 1.15e-60
72. KAG5989301.1 2.15e-60
73. KAG5949601.1 1.95e-58
74. KAG5994791.1 1.76e-58
75. KAG8422976.1 6.32e-64
76. XP_040683175.1 2.18e-48
77. KJK80520.1 8.93e-62
78. XP_014548452.1 2.40e-63
79. KID92007.1 2.36e-64
80. KAH0597174.1 1.98e-62
81. OAA43280.1 4.04e-58
82. XP_007822448.1 4.68e-63
83. XP_042995238.1 5.26e-58
84. KAK2616729.1 3.76e-64
85. KZZ90826.1 1.92e-60
86. QPH11511.1 3.98e-53
87. RFU78995.1 5.34e-56
88. KAH8127233.1 2.83e-59
89. UKZ86906.1 1.62e-59
90. UKZ67550.1 2.95e-57
91. XP_056030790.1 4.76e-59
92. XP_024752346.1 2.33e-59
93. KAH6605774.1 4.33e-60
94. KAH0492740.1 1.33e-59
95. OPB36448.1 2.14e-57
96. KKO97596.1 9.53e-58
97. PTB77621.1 1.35e-60
98. XP_006968899.1 5.54e-61
99. KAH0529301.1 1.41e-57
100. QYS95815.1 2.36e-59
0
100
200
300
400
500

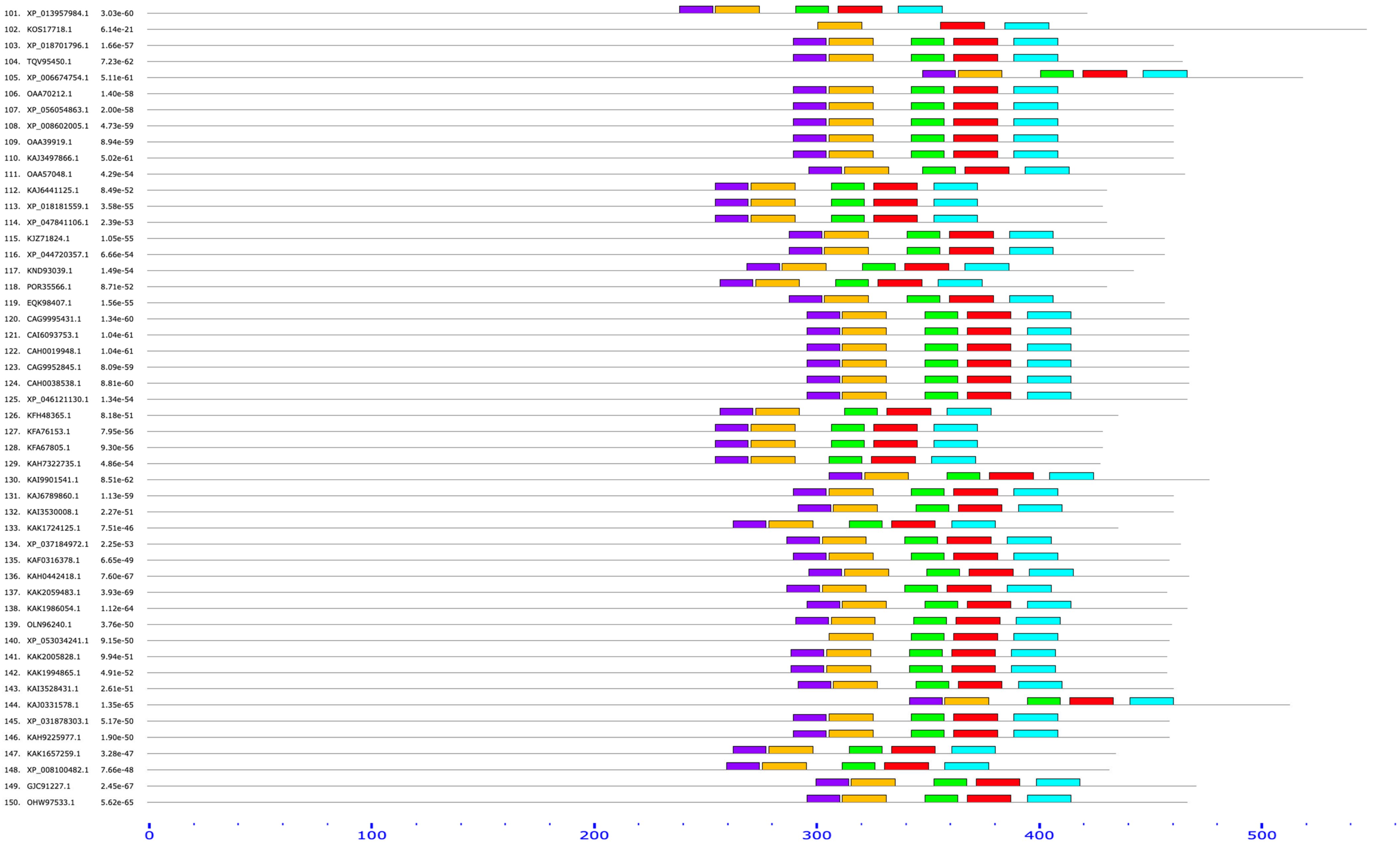

101. XP_013957984.1 3.03e-60
102. KOS17718.1 6.14e-21
103. XP_018701796.1 1.66e-57
104. TQV95450.1 7.23e-62
105. XP_006674754.1 5.11e-61
106. OAA70212.1 1.40e-58
107. XP_056054863.1 2.00e-58
108. XP_008602005.1 4.73e-59
109. OAA39919.1 8.94e-59
110. KAJ3497866.1 5.02e-61
111. OAA57048.1 4.29e-54
112. KAJ6441125.1 8.49e-52
113. XP_018181559.1 3.58e-55
114. XP_047841106.1 2.39e-53
115. KJZ71824.1 1.05e-55
116. XP_044720357.1 6.66e-54
117. KND93039.1 1.49e-54
118. POR35566.1 8.71e-52
119. EQK98407.1 1.56e-55
120. CAG9995431.1 1.34e-60
121. CAI6093753.1 1.04e-61
122. CAH0019948.1 1.04e-61
123. CAG9952845.1 8.09e-59
124. CAH0038538.1 8.81e-60
125. XP_046121130.1 1.34e-54
126. KFH48365.1 8.18e-51
127. KFA76153.1 7.95e-56
128. KFA67805.1 9.30e-56
129. KAH7322735.1 4.86e-54
130. KAI9901541.1 8.51e-62
131. KAJ6789860.1 1.13e-59
132. KAI3530008.1 2.27e-51
133. KAK1724125.1 7.51e-46
134. XP_037184972.1 2.25e-53
135. KAF0316378.1 6.65e-49
136. KAH0442418.1 7.60e-67
137. KAK2059483.1 3.93e-69
138. KAK1986054.1 1.12e-64
139. OLN96240.1 3.76e-50
140. XP_053034241.1 9.15e-50
141. KAK2005828.1 9.94e-51
142. KAK1994865.1 4.91e-52
143. KAI3528431.1 2.61e-51
144. KAJ0331578.1 1.35e-65
145. XP_031878303.1 5.17e-50
146. KAH9225977.1 1.90e-50
147. KAK1657259.1 3.28e-47
148. XP_008100482.1 7.66e-48
149. GJC91227.1 2.45e-67
150. OHW97533.1 5.62e-65
0
100
200
300
400
500

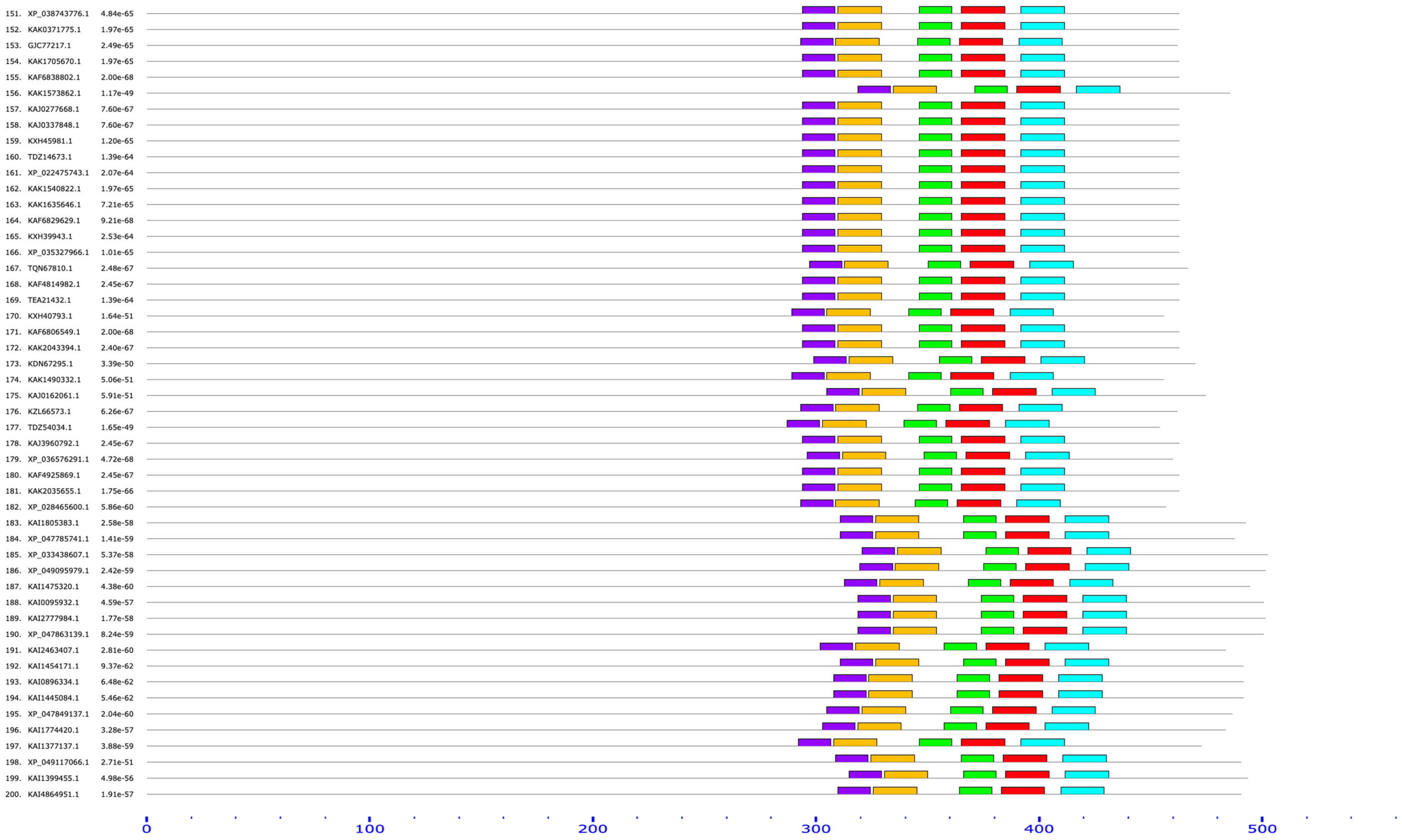
151. XP_038743776.1 4.84e-65
152. KAK0371775.1 1.97e-65
153. GJC77217.1 2.49e-65
154. KAK1705670.1 1.97e-65
155. KAF6838802.1 2.00e-68
156. KAK1573862.1 1.17e-49
157. KAJ0277668.1 7.60e-67
158. KAJ0337848.1 7.60e-67
159. KXH45981.1 1.20e-65
160. TDZ14673.1 1.39e-64
161. XP_022475743.1 2.07e-64
162. KAK1540822.1 1.97e-65
163. KAK1635646.1 7.21e-65
164. KAF6829629.1 9.21e-68
165. KXH39943.1 2.53e-64
166. XP_035327966.1 1.01e-65
167. TQN67810.1 2.48e-67
168. KAF4814982.1 2.45e-67
169. TEA21432.1 1.39e-64
170. KXH40793.1 1.64e-51
171. KAF6806549.1 2.00e-68
172. KAK2043394.1 2.40e-67
173. KDN67295.1 3.39e-50
174. KAK1490332.1 5.06e-51
175. KAJ0162061.1 5.91e-51
176. KZL66573.1 6.26e-67
177. TDZ54034.1 1.65e-49
178. KAJ3960792.1 2.45e-67
179. XP_036576291.1 4.72e-68
180. KAF4925869.1 2.45e-67
181. KAK2035655.1 1.75e-66
182. XP_028465600.1 5.86e-60
183. KAI1805383.1 2.58e-58
184. XP_047785741.1 1.41e-59
185. XP_033438607.1 5.37e-58
186. XP_049095979.1 2.42e-59
187. KAI1475320.1 4.38e-60
188. KAI0095932.1 4.59e-57
189. KAI2777984.1 1.77e-58
190. XP_047863139.1 8.24e-59
191. KAI2463407.1 2.81e-60
192. KAI1454171.1 9.37e-62
193. KAI0896334.1 6.48e-62
194. KAI1445084.1 5.46e-62
195. XP_047849137.1 2.04e-60
196. KAI1774420.1 3.28e-57
197. KAI1377137.1 3.88e-59
198. XP_049117066.1 2.71e-51
199. KAI1399455.1 4.98e-56
200. KAI4864951.1 1.91e-57
0
100
200
300
400
500

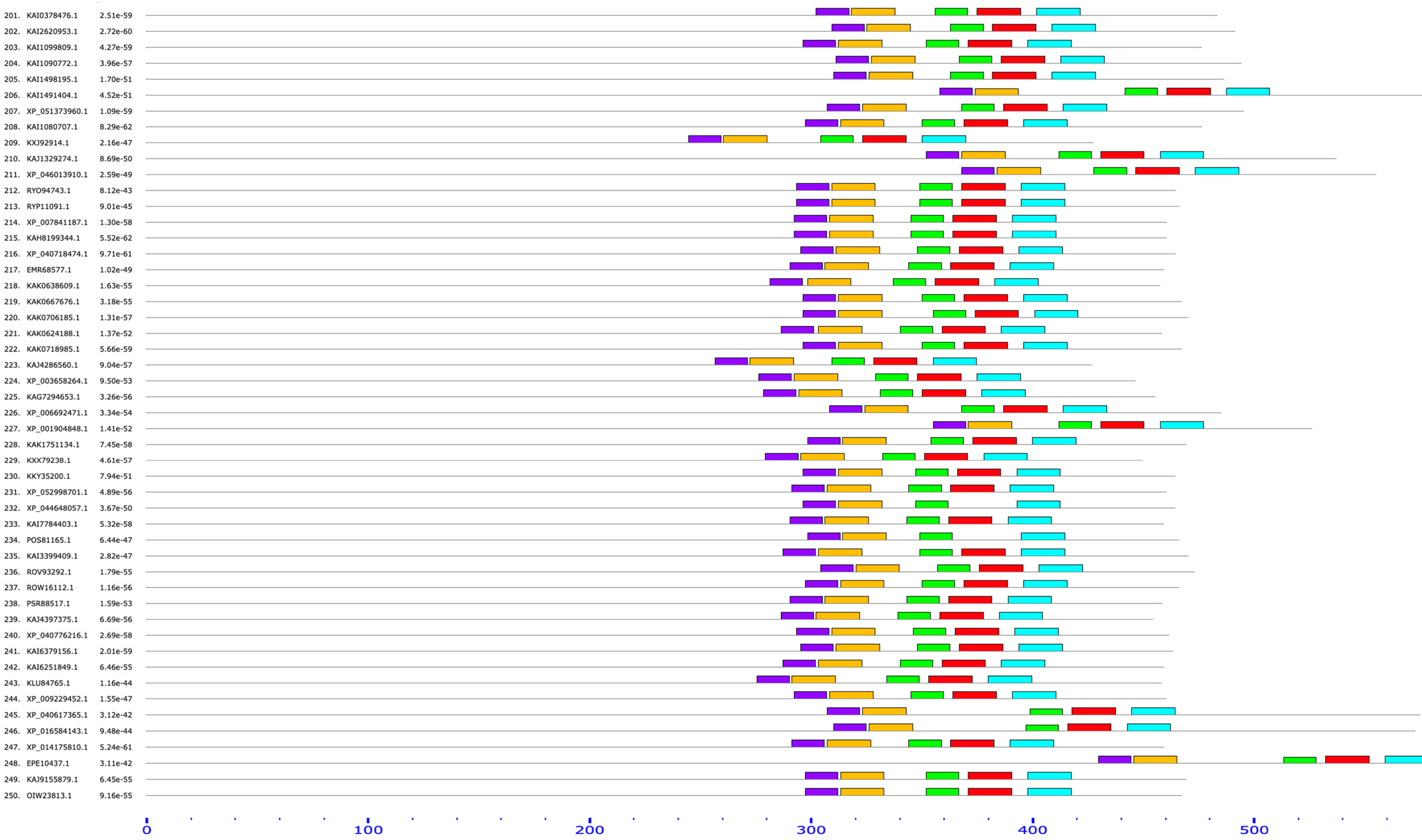

201. KAI0378476.1 2.51e-59
202. KAI2620953.1 2.72e-60
203. KAI1099809.1 4.27e-59
204. KAI1090772.1 3.96e-57
205. KAI1498195.1 1.70e-51
206. KAI1491404.1 4.52e-51
207. XP_051373960.1 1.09e-59
208. KAI1080707.1 8.29e-62
209. KXJ92914.1 2.16e-47
210. KAJ1329274.1 8.69e-50
211. XP_046013910.1 2.59e-49
212. RYO94743.1 8.12e-43
213. RYP11091.1 9.01e-45
214. XP_007841187.1 1.30e-58
215. KAH8199344.1 5.52e-62
216. XP_040718474.1 9.71e-61
217. EMR68577.1 1.02e-49
218. KAK0638609.1 1.63e-55
219. KAK0667676.1 3.18e-55
220. KAK0706185.1 1.31e-57
221. KAK0624188.1 1.37e-52
222. KAK0718985.1 5.66e-59
223. KAJ4286560.1 9.04e-57
224. XP_003658264.1 9.50e-53
225. KAG7294653.1 3.26e-56
226. XP_006692471.1 3.34e-54
227. XP_001904848.1 1.41e-52
228. KAK1751134.1 7.45e-58
229. KXX79238.1 4.61e-57
230. KKY35200.1 7.94e-51
231. XP_052998701.1 4.89e-56
232. XP_044648057.1 3.67e-50
233. KAI7784403.1 5.32e-58
234. POS81165.1 6.44e-47
235. KAI3399409.1 2.82e-47
236. ROV93292.1 1.79e-55
237. ROW16112.1 1.16e-56
238. PSR88517.1 1.59e-53
239. KAJ4397375.1 6.69e-56
240. XP_040776216.1 2.69e-58
241. KAI6379156.1 2.01e-59
242. KAI6251849.1 6.46e-55
243. KLU84765.1 1.16e-44
244. XP_009229452.1 1.55e-47
245. XP_040617365.1 3.12e-42
246. XP_016584143.1 9.48e-44
247. XP_014175810.1 5.24e-61
248. EPE10437.1 3.11e-42
249. KAJ9155879.1 6.45e-55
250. OIW23813.1 9.16e-55
0
100
200
300
400
500

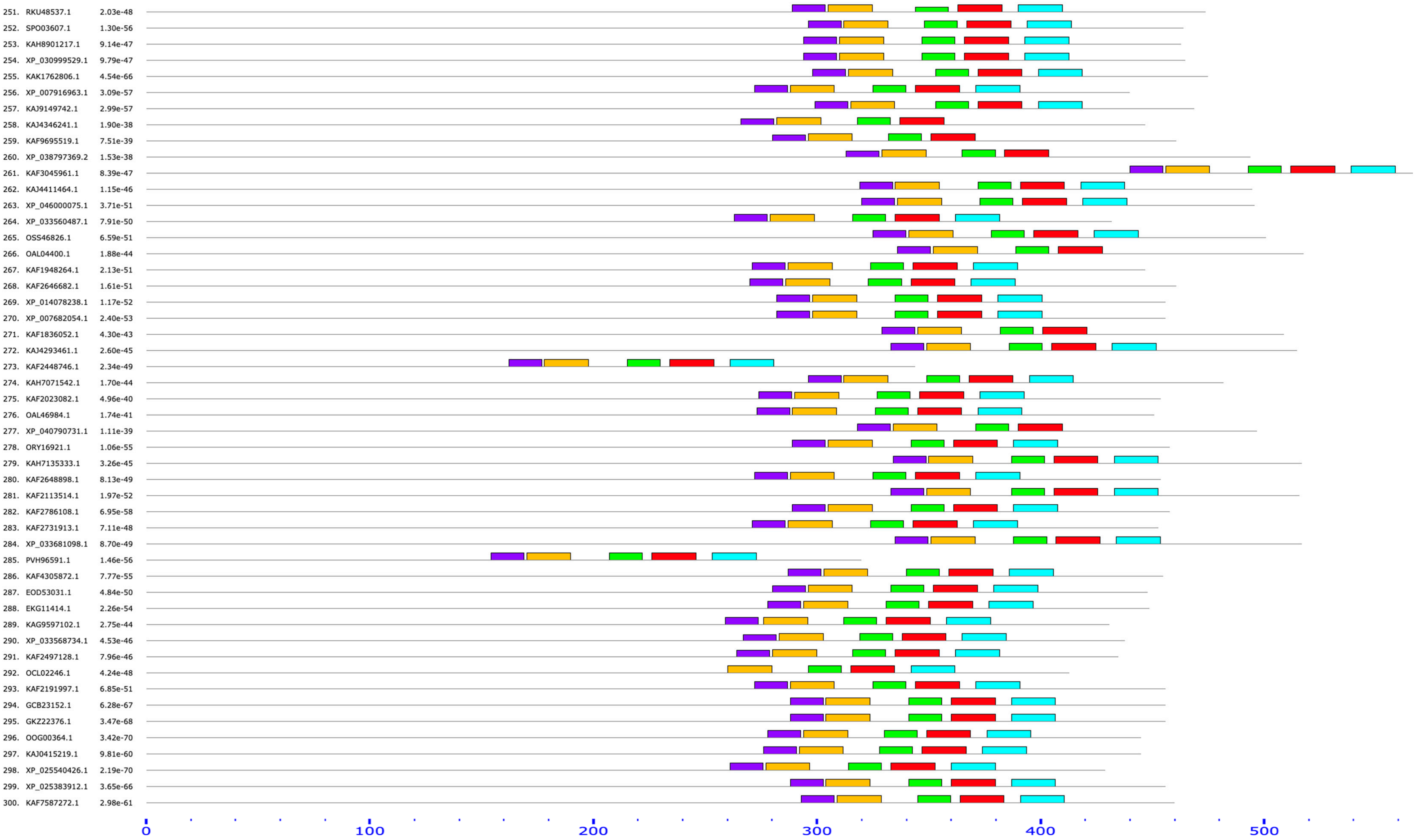
251. RKU48537.1 2.03e-48
252. SPO03607.1 1.30e-56
253. KAH8901217.1 9.14e-47
254. XP_030999529.1 9.79e-47
255. KAK1762806.1 4.54e-66
256. XP_007916963.1 3.09e-57
257. KAJ9149742.1 2.99e-57
258. KAJ4346241.1 1.90e-38
259. KAF9695519.1 7.51e-39
260. XP_038797369.2 1.53e-38
261. KAF3045961.1 8.39e-47
262. KAJ4411464.1 1.15e-46
263. XP_046000075.1 3.71e-51
264. XP_033560487.1 7.91e-50
265. OSS46826.1 6.59e-51
266. OAL04400.1 1.88e-44
267. KAF1948264.1 2.13e-51
268. KAF2646682.1 1.61e-51
269. XP_014078238.1 1.17e-52
270. XP_007682054.1 2.40e-53
271. KAF1836052.1 4.30e-43
272. KAJ4293461.1 2.60e-45
273. KAF2448746.1 2.34e-49
274. KAH7071542.1 1.70e-44
275. KAF2023082.1 4.96e-40
276. OAL46984.1 1.74e-41
277. XP_040790731.1 1.11e-39
278. ORY16921.1 1.06e-55
279. KAH7135333.1 3.26e-45
280. KAF2648898.1 8.13e-49
281. KAF2113514.1 1.97e-52
282. KAF2786108.1 6.95e-58
283. KAF2731913.1 7.11e-48
284. XP_033681098.1 8.70e-49
285. PVH96591.1 1.46e-56
286. KAF4305872.1 7.77e-55
287. EOD53031.1 4.84e-50
288. EKG11414.1 2.26e-54
289. KAG9597102.1 2.75e-44
290. XP_033568734.1 4.53e-46
291. KAF2497128.1 7.96e-46
292. OCL02246.1 4.24e-48
293. KAF2191997.1 6.85e-51
294. GCB23152.1 6.28e-67
295. GKZ22376.1 3.47e-68
296. OOG00364.1 3.42e-70
297. KAJ0415219.1 9.81e-60
298. XP_025540426.1 2.19e-70
299. XP_025383912.1 3.65e-66
300. KAF7587272.1 2.98e-61
0
100
200
300
400
500

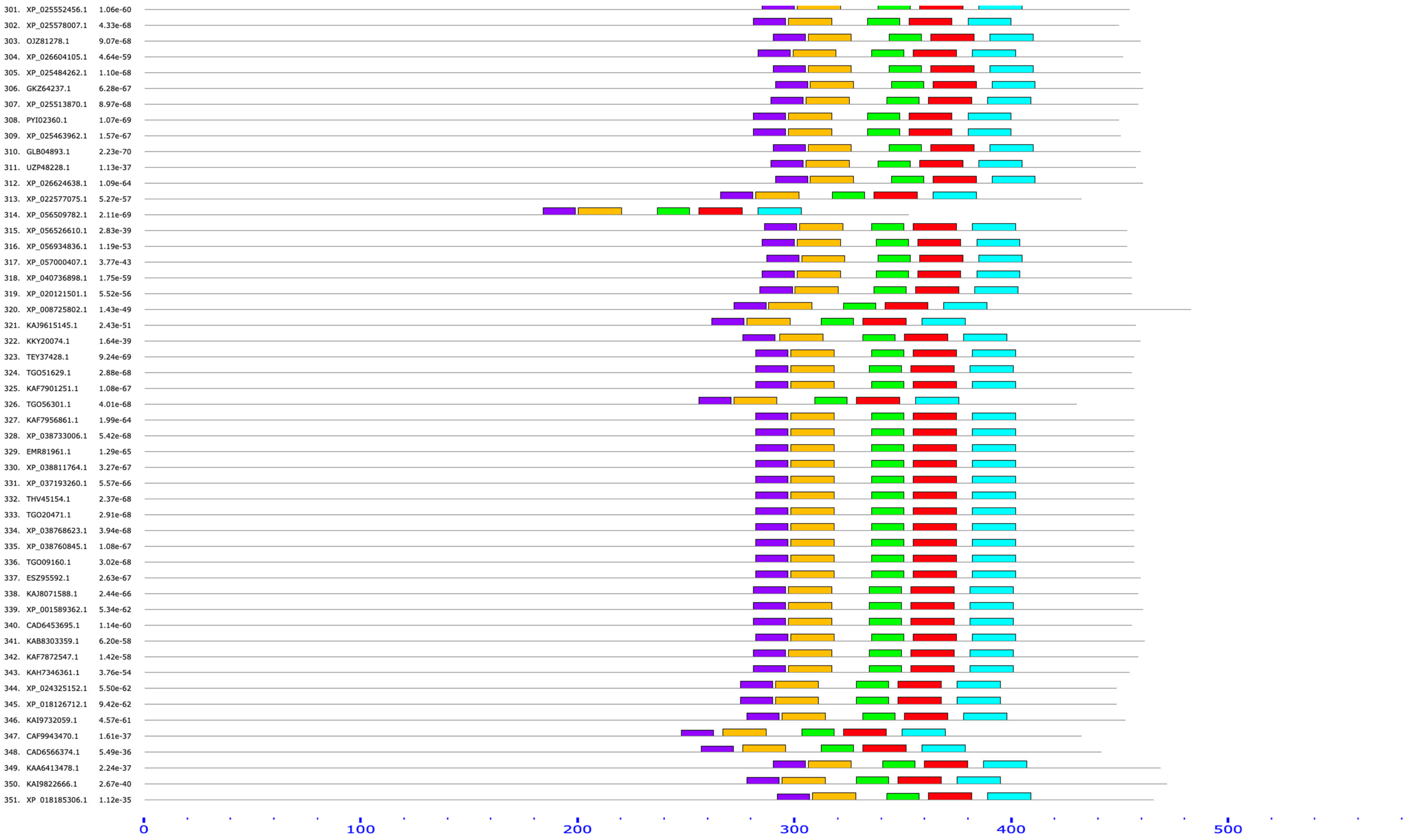
301. XP_025552456.1 1.06e-60
302. XP_025578007.1 4.33e-68
303. OJZ81278.1 9.07e-68
304. XP_026604105.1 4.64e-59
305. XP_025484262.1 1.10e-68
306. GKZ64237.1 6.28e-67
307. XP_025513870.1 8.97e-68
308. PYI02360.1 1.07e-69
309. XP_025463962.1 1.57e-67
310. GLB04893.1 2.23e-70
311. UZP48228.1 1.13e-37
312. XP_026624638.1 1.09e-64
313. XP_022577075.1 5.27e-57
314. XP_056509782.1 2.11e-69
315. XP_056526610.1 2.83e-39
316. XP_056934836.1 1.19e-53
317. XP_057000407.1 3.77e-43
318. XP_040736898.1 1.75e-59
319. XP_020121501.1 5.52e-56
320. XP_008725802.1 1.43e-49
321. KAJ9615145.1 2.43e-51
322. KKY20074.1 1.64e-39
323. TEY37428.1 9.24e-69
324. TGO51629.1 2.88e-68
325. KAF7901251.1 1.08e-67
326. TGO56301.1 4.01e-68
327. KAF7956861.1 1.99e-64
328. XP_038733006.1 5.42e-68
329. EMR81961.1 1.29e-65
330. XP_038811764.1 3.27e-67
331. XP_037193260.1 5.57e-66
332. THV45154.1 2.37e-68
333. TGO20471.1 2.91e-68
334. XP_038768623.1 3.94e-68
335. XP_038760845.1 1.08e-67
336. TGO09160.1 3.02e-68
337. ESZ95592.1 2.63e-67
338. KAJ8071588.1 2.44e-66
339. XP_001589362.1 5.34e-62
340. CAD6453695.1 1.14e-60
341. KAB8303359.1 6.20e-58
342. KAF7872547.1 1.42e-58
343. KAH7346361.1 3.76e-54
344. XP_024325152.1 5.50e-62
345. XP_018126712.1 9.42e-62
346. KAI9732059.1 4.57e-61
347. CAF9943470.1 1.61e-37
348. CAD6566374.1 5.49e-36
349. KAA6413478.1 2.24e-37
350. KAI9822666.1 2.67e-40
351. XP_018185306.1 1.12e-35
0
100
200
300
400
500
